# A feedforward circuit shaped by ECT2 and USP7 contributes to breast carcinogenesis

**DOI:** 10.7150/thno.46878

**Published:** 2020-08-29

**Authors:** Qi Zhang, Cheng Cao, Wenchen Gong, Kaiwen Bao, Qian Wang, Yuejiao Wang, Liyuan Bi, Shuai Ma, Jiao Zhao, Ling Liu, Shanshan Tian, Kai Zhang, Jie Yang, Zhi Yao, Nan Song, Lei Shi

**Affiliations:** 1 State Key Laboratory of Experimental Hematology, 2011 Collaborative Innovation Center of Tianjin for Medical Epigenetics, Key Laboratory of Immune Microenvironment and Disease (Ministry of Education), Key Laboratory of Breast Cancer Prevention and Therapy (Ministry of Education), Tianjin Medical University Cancer Institute and Hospital, Tianjin Medical University General Hospital, Department of Biochemistry and Molecular Biology, School of Basic Medical Sciences, Tianjin Medical University, Tianjin 300070, China.; 2 Qingdao Haici Medical Treatment Group, Qingdao, 266000, China.

**Keywords:** Guanine nucleotide exchange factors, Deubiquitination, Deubiquitinase, Protein stability, Breast cancer

## Abstract

**Rationale:** A number of guanine nucleotide exchange factors (GEFs) including epithelial cell transforming factor ECT2 are believed to drive carcinogenesis through activating distinct oncogenic GTPases. Yet, whether GEF-independent activity of ECT2 also plays a role in tumorigenesis remains unclear.

**Methods:** Immunohistochemical (IHC) staining, colony formation and xenograft assays were used to examine the role of ECT2 in breast carcinogenesis. Co-immunoprecipitation, immunofluorescent stainings, *in vivo* deubiquitination and *in vitro* deubiquitination experiments were performed to examine the physical and functional interaction between ECT2 and ubiquitin-specific protease USP7. High-throughput RNA sequencing, quantitative reverse transcription-PCR and Western blotting were employed to investigate the biological significance of the interplay between ECT2 and USP7.

**Results:** We report that ECT2 plays a tumor-promoting role in breast cancer, and GEF activity-deficient ECT2 is able to alleviate ECT2 depletion associated growth defects in breast cancer cells. Mechanistically, we demonstrated that ECT2 physically interacts with ubiquitin-specific protease USP7 and functionally facilitates USP7 intermolecular self-association, -deubiquitination and -stabilization in a GEF activity-independent manner. USP7 in turn, deubiquitinates and stabilizes ECT2, resulting in a feedforward regulatory circuit that ultimately sustains the expression of oncogenic protein MDM2.

**Conclusion:** Our study uncovers a GEF-independent role of ECT2 in promoting survival of breast cancer cells, provides a molecular insight for the reciprocal regulation of ECT2 and USP7, and supports the pursuit of ECT2/USP7 as potential targets for breast cancer intervention.

## Introduction

The Ras superfamily of small GTPases are the founding members of a large superfamily of monomeric small (20-25 kDa) GTPases, aberrant activity of which is believed to play a causal role in multiple types of human cancers 1, 2. The Ras superfamily comprises more than 150 members in humans and regulates diverse cellular processes, including cell motility, polarity, growth, and survival 3, 4. A common feature of GTPase deregulation in cancer is the deregulated expression and/or activity of their regulatory proteins, which together with GTPases to constitute a trimeric form of machinery that acts as cellular GDP/GTP-regulated binary switches 2. In particular, guanine nucleotide exchange factors (GEFs) accelerate the exchange of GDP for GTP thus formation of the active GTP-bound state of GTPases, while GTPase-activating proteins (GAPs) terminate the active state by stimulating the intrinsic GTP hydrolysis activity of GTPases 5.

A major branch of the Ras GTPases is the Ras homologous (Rho) family proteins 6, among which RhoA, Rac family small GTPase 1 (RAC1) and cell division cycle 42 (CDC42) are the most extensively studied and characterized 7. Epithelial cell transforming sequence 2 (ECT2), also known as ARHGEF31, is a RhoGEF activator primarily of RhoA, also of RAC1 and CDC42 1, 7, 8. The N-terminus of ECT2 contains two tandem BRCA1 C-terminal (BRCT) domains and the central catalytic portion in the C-terminus of ECT2 consists of Dbl homology (DH) catalytic and pleckstrin homology (PH) regulatory domain 1, 5, 9. Unlike other known RhoGEFs, ECT2 exhibits diverse and dynamic subcellular localization during the cell cycle. It predominantly localizes in nucleus in interphase, disperses throughout the cytoplasm in prometaphase, and then accumulates in the midbody during cytokinesis 10. By means of temporospatial distribution and association with distinct Rho GTPases, ECT2 regulates diverse biochemical and physiological processes. Specifically, ECT2 regulates RhoA in non-transformed cells during cytokinesis 11, while it coordinates with the protein kinase Cι (PKCι)-PAR6 complex to drive transformation growth by activating RAC1 12, 13. During interphase of the cell cycle, ECT2 on one side activates nuclear CDC42 to keep surveillance and stabilize newly incorporated histone variant CENP-A at centromeres 14, while on the other side, it recruits nuclear RAC1 and nucleophosmin (NPM) to activate rRNA synthesis 15.

ECT2 is initially identified as an oncogene in an NIH 3T3 focus formation assay using a cDNA library from the Balb/MK mouse keratinocyte cell line 16, 17. Nowadays ECT2 has been implicated in multiple disorders including distinct types of cancers 18, 19. Dysregulated expression of ECT2 has been reported in glioblastoma 20 and carcinomas of lung 15, 21, esophageal 19, oral squamous cell 22, colorectal 23, and others 8, and the tumor-promoting effect of ECT2 is largely attributed to its GEF activity towards distinct Rho GTPases in a context-dependent manner 12, 24, 25. Early observations suggested that ECT2 is auto-inhibited in the nucleus by its N-terminal BRCT domains 26, 27, whereas a recent study suggested that the mechanisms that account for ECT2 in driving cancer progression also involve its nuclear GEF activity 15, implying that the active and inactive pool of nuclear ECT2 possibly exists simultaneously or switches dynamically.

Adding further complexity to the contribution of RhoGEF activators to the biology of cancer, recent observations suggest that a fraction of RhoGEFs can also trigger GTPase-independent pro- or anti-tumorigenic functions 28. Examples include the catalysis-independent stimulation of the RAS/MAPK pathway by ARHGEF2 29, the RAS/PI3K/AKT axis by PREX2 30, the YAP/TAZ complex by ARHGEF7 31, 32, the nuclear factor of activated T cells by VAV1, and the androgen receptor by VAV3 33. These non-canonical roles are probably more widespread, given that some RhoGEFs (e.g. VAV3) generate splicing isoforms lacking the catalytic domains 28 and some of them (e.g. TRIO and SOS1) contain functional domains other than DH-PH 7. Although elevated expression of ECT2 in breast cancer has been reported 34 and ECT2 is linked to dissemination and metastasis of breast cancer cells via controlling RhoA and CDC42 24, 35, it remains obscure whether nuclear GEF-independent activity of ECT2 also plays a role in the progression and development of breast cancer.

The ubiquitin system controls protein turnover through providing proteasomal targeting signals, while the deubiquitinating enzymes (DUBs, also known as deubiquitinases) maintain protein stability by releasing conjugated ubiquitins from targeted substrates 36-38. Thus, the balance between ubiquitination and deubiquitination is tightly coupled to the regulation of protein levels. The ubiquitin-specific protease USP7 is able to cleave multiple types of lysine (K)-conjugated ubiquitin chains such as K48-linked and K63-linked ubiquitin moieties 39, 40. In this manner, it acts on a large number of targets to regulate diverse cellular activities, ranging from DNA damage response to immune response, and drives pathological processes including multiple malignancies 41-43. USP7 consists of an N-terminal meprin and tumor necrosis factor-receptor associated factor (TRAF) homology (MATH) domain, a middle catalytic deubiquitinase (CD) domain, and five consecutive ubiquitin-like (UBL) domains on the C-terminal side. Structure analysis revealed that the C-terminal UBL domain of USP7 positively regulates its catalytic activity and this effect can be further allosterically enhanced by the metabolic enzyme GMP-synthetase (GMPS) 44, 45. Proteins like DNMT1 46, ICP0 47 and MDC1 48 also interact primarily with the UBL domain of USP7, while MATH domain is critical for the recruitment of its targets like p53, MDM2 49, 50 or PHF8 42. Interestingly, USP7 is proposed to exist in an oligomeric form like other deubiquitinases 51, 52, but the rationality and related regulatory mechanisms are still unknown.

In this study, we uncovered a nuclear GEF-independent role of ECT2 in promoting survival of breast cancer cells. Mechanistically, ECT2 together with USP7, forms a positive feedback loop to control the expression of each other, and the reciprocal stabilization of the two molecules fuels oncogenic MDM2 in breast cancer.

## Methods

### Antibodies and Reagents

The sources of antibodies against the following proteins were as follows: β-actin (A1978) and FLAG (F3165) from Sigma; ECT2 (07-1364) from Millipore; PHF8 (A301-772A) and USP7 (A300-033A, for WB, IF, IP and IHC) from Bethyl Lab; USP7 (05-1946 for WB) from Sigma; RNF168 (21393-1-AP), USP11 (10244-1-AP), RAD18 (18333-1-AP), UHRF1 (21402-1-AP), and His (66005-1-Ig) from Proteintech; Myc (M047-3) from MBL; MDM2 (ab38618, WB and IHC), CDC42 (ab187642), RAC1 (ab33186) from Abcam; GFP (YM3124) from Immunoway; and ubiquitin (OM294553) from OmnimAbs. Anti-FLAG M2 affinity gel (A2220), 3 × FLAG peptide (F4799), MG132 (SML1135), neomycin (N1142), blasticidin (15205), puromycin (P8833) and doxycycline (D9891) were purchased from Sigma. Ni-NTA Purification System (K950-01) was purchased from Thermo Fisher. K48-linked tetra-ubiquitin chains (UC-210B) and K63-linked tetra-ubiquitin chains (UC-310B) were purchased from Boston Biochem. CHX and HBX 41,108 were purchased from TOCRIS. GNE-6640 was purchased from Glixx Laboratories.

### Plasmids

The FLAG or Myc tagged USP7/wt carried by pLVX-Tight-Puro, pLenti-hygro vector were amplified from USP7 cDNA kindly provided by Dr. Yang Shi (Harvard Medical School, Boston) and Dr. Ruaidhri J. Carmody (University of Glasgow, Scotland, UK), while FLAG tagged USP7 truncations were generated by PCR cloning and carried by pLenti vector. The FLAG, Myc or GFP tagged USP7/C223S were generated by quick change point mutation assay. GFP tagged USP7/wt and USP7/C233S was carried by pLVX-Tight-Puro vector, while His tagged USP7/wt and USP7/C223S were carried by pFastBac-HTA vector. The full length of USP7 and ECT2 were PCR amplified and integrated to mCherry-LacI vector (Addgene plasmid # 18985), which was a gift from Dr. Mirek Dundr (Rosalind Franklin University of Medicine and Science). The Myc-ECT2 or FLAG-ECT2 was amplified from ECT2 cDNA (Open Biosystem) and cloned into pLenti-hygro or pLVX-Tight-Puro vector, respectively, while ECT2/GEF^mt^ were generated by quick change point mutation assay. FLAG tagged ECT2 truncations were generated by PCR cloning and carried by pLenti vector. The FLAG tagged MDM2 was amplified from pCMV6-MDM2 (Origene) and carried by pLenti-hygro vector. The Rluc and EYFP kindly provided by Dr. Ding Ai (Tianjin Medical University, Tianjin, China) were PCR amplified and individually integrated into the C-terminal of USP7 in pcDNA3.1 vector. His tagged Ub/wt, Ub/mt and K48R in pcDNA3.1 vector were gifts from Dr. Ping Wang (Tongji University, Shanghai, China). His tagged Ub/K48 only was chemically synthesized and integrated into pcDNA3.1 vector (General Bio). CRISPR/Cas9 constructs lentiCas9-Blast (Addgene plasmid # 52962) and lentiGuide-Puro (Addgene plasmid # 52963) were gifts from Dr. Feng Zhang (Broad Institute, Cambridge).

### Cell Culture

MCF-7, HeLa, HEK293T, ZR-75-1, MDA-MB-468 and Sf9 cells were got from the American Type Culture Collection (Manassas, VA) and cultured under the manufacturer's instructions. The LacO-LacI U2OS cells were kindly provide by Dr. Roger Greenberg (University of Pennsylvania, Pennsylvania). Cells that allow protein expression under doxycycline treatment were created in two steps. First, cells were infected with lentivirus carrying rtTA and subjected to neomycin selection. Subsequently, the established rtTA cells were infected with virus carrying pLVX-Tight-Puro vector that encodes USP7 or ECT2, followed by puromycin selection. All of the cells integrated with rtTA were cultured in Tet Approved FBS and medium from Clontech. All of the cells were authenticated by examination of morphology and growth characteristics, and were confirmed to be mycoplasma-free.

### Western Blotting

Whole cell lysates were harvested from treated cells followed by re-suspending in 5 × SDS-PAGE loading buffer. The boiled protein samples were then subjected to SDS-PAGE followed by immunoblotting with appropriately primary antibodies and secondary antibodies. The uncropped blots related to Figures 1-6 and Supplemental Figures 1-5 have been provided in Figure S6.

### Immunopurification and Silver Staining

Lysates from MCF-7 cells stably expressing FLAG-ECT2 were prepared by incubating the cells in lysis buffer containing protease inhibitor Cocktail (Roche). Anti-FLAG immunoaffinity columns were prepared using anti-FLAG M2 affinity gel (Sigma) following the manufacturer's suggestions. Cell lysates were obtained from about 5 × 10^8^ cells and applied to an equilibrated FLAG column of 1 mL bed volume to allow for adsorption of the protein complex to the column resin. After binding, the column was washed with cold PBS plus 0.2% Nonidet P-40. FLAG peptide (Sigma) was applied to the column to elute the FLAG protein complex as described by the vendor. The eluents were collected and visualized on NuPAGE 4-12% Bis-Tris gel (Thermo Fisher) followed by silver staining with silver staining kit (Pierce). The distinct protein bands were retrieved and analyzed by LC-MS/MS.

### Nano-HPLC-MS/MS Analysis of ECT2-Containing Protein Complex

To identify proteins associated with FLAG-ECT2, LC-MS/MS analysis was performed using a Thermo Finnigan LTQ linear ion trap mass spectrometer in line with a Thermo Finnigan Surveyor MS Pump Plus HPLC system. Tryptic peptides generated were loaded onto a trap column (300SB-C18, 5 × 0.3 mm, 5 μm particle; Agilent Technologies, Santa Clara CA) which was connected through a zero dead volume union to the self-packed analytical column (C18, 100 μm i.d × 100 mm, 3 μm particle; SunChrom, Germany). The peptides were then eluted over a gradient (0-45% B in 55 min, 45-100% B in 10 min, where B = 80% Acetonitrile, 0.1% formic acid) at a flow rate of 500 nL min^-1^ and introduced online into the linear ion trap mass spectrometer (Thermo Fisher Corporation, San Jose, CA) using nano electrospray ionization (ESI). Data dependent scanning was incorporated to select the five most abundant ions (one microscan per spectra; precursor isolation width 1.0 m/z, 35% collision energy, 30 ms ion activation, exclusion duration: 90 s; repeat count: 1) from a full-scan mass spectrum for fragmentation by collision induced dissociation (CID). MS data were analysed using SEQUEST (v. 28) against NCBI human protein database (Dec, 14, 2011 downloaded, 33,256 entries), and results were filtered, sorted, and displayed using the Bioworks 3.2. Peptides (individual spectra) with Preliminary Score (Sp) ≥ 500; Rank of Sp (RSp) ≤ 5; and peptides with + 1, + 2, or + 3 charge states were accepted if they were fully enzymatic and had a cross correlation (Xcorr) of 1.90, > 2.75, and > 3.50, respectively. The following residue modifications were allowed in the search: carbamidomethylation on cysteine as fix modification and oxidation on methionine as variable modification. Peptide sequences were searched using trypsin specificity and allowing a maximum of two missed cleavages. Sequest was searched with a peptide tolerance of 3.0 Da and a fragment ion tolerance of 1.0 Da.

### Immunoprecipitation

Cellular lysates were prepared by incubating the cells in NETN buffer (50 mM Tris-HCl, pH 8.0, 150 mM NaCl, 0.2% Nonidet P-40, 2 mM EDTA) in the presence of protease inhibitor Cocktails (Roche) for 20 min at 4 °C followed by centrifugation at 14,000 g for 15 min at 4 °C. For immunoprecipitation, about 500 μg of protein was incubated with control or specific antibodies (1-2 μg) for 12 h at 4 °C with constant rotation; 50 μL of 50% protein G magnetic beads (Thermo Fisher) was then added and the incubation was continued for an additional 2 h. Beads were then washed five times using the lysis buffer. Between washes, the beads were collected by magnetic stand (Thermo Fisher) at 4 °C. The precipitated proteins were eluted from the beads by re-suspending the beads in 2 × SDS-PAGE loading buffer and boiling for 5 min. The boiled immune complexes were subjected to SDS-PAGE followed by immunoblotting with appropriate antibodies.

### Recombinant Protein Purification

Recombinant baculovirus carrying USP7/wt, USP7/C223S or deletion mutants was generated with the Bac-to-Bac System (Thermo Fisher). Infected Sf9 cells were grown in spinner culture for 48 to 96 h at 27 °C and lysed by ultrasonicator in Equilibration buffer (50 mM sodium phosphate, 0.3 M sodium chloride, 10 mM imidazole, and 10 mM Tris-HCl, pH 8.0). His-tagged proteins were purified using Ni-NTA agarose (Thermo Fisher) according to standard procedures.

### *In vivo* Deubiquitination Assay

Cells under different treatments were lysed in buffer A (6 M guanidine-HCl, 0.1 M Na2HPO4/NaH2PO4, 10 mM imidazole, pH 8.0) in the presence of protease inhibitors at 4 °C for 30 min with rotation, and centrifuged at 20,000 g for 15 min. The ubiquitinated proteins were purified using Ni-NTA beads (Thermo Fisher) for 2 h, then the beads were washed sequentially with Buffer A, Buffer B (8 M urea, 0.1 M Na2HPO4/NaH2PO4, pH 8.0, 0.01 M Tris-HCl, pH 8.0, 10 mM β-mercaptoethanol), Buffer C+100 (Buffer C containing 0.2% Triton X-100), and Buffer C (8 M urea, 0.1 M Na2HPO4/NaH2PO4, pH 6.3, 0.01 M Tris-HCl, pH 6.3, 10 mM β-mercaptoethanol). The washed beads were incubated in 40 μL elution buffer (200 mM imidazole, 0.15 M Tris-HCl, pH 6.7, 30% glycerol, 5% SDS, 0.72 M β-mercaptoethanol) at room temperature for 30 min, then boiled in SDS loading buffer and subjected to SDS-PAGE followed by immunoblotting.

### *In vitro* Deubiquitination Assay

HeLa cells expressing His-ubiquitin and FLAG tagged USP7/C223S or ECT2 were collected and the lysate was incubated with anti-FLAG affinity gel for 2 h and the beads were then washed five times with RIPA Buffer (300 mM NaCl, 0.5% sodium deoxycholate, 0.1% SDS, 1% Nonidet P-40, and 50 mM Tris-HCl, pH 8.0), eluted with 3 × FLAG peptide and then subjected to Ni-NTA affinity beads to enrich His-Ub conjugated proteins. The beads were then washed sequentially with Buffer A, Buffer B, Buffer C+100 and Buffer C. The washed beads were incubated in 60 μL elution buffer (200 mM imidazole, 0.15 M Tris-HCl, pH 6.7, 30% glycerol, 5% SDS, 0.72 M β-mercaptoethanol) at room temperature for 30 min. Recombinant USP7 and USP7/C223S-Ub or ECT2-Ub were incubated in DUB buffer (50 mM HEPES, pH 7.5, 10 mM β-mercaptoethanol and 0.5 mM EDTA) at 37 ºC for 4 h. The reactions were stopped by boiling for 5 min in 5 × SDS-PAGE loading buffer followed by Western blotting. Analogously, recombinant USP7 was incubated with different types of homogenous ubiquitin linkages in DUB buffer followed by Western blotting analysis.

### Blot Quantitation

For endogenous proteins, then intensity of USP7 was quantified by Image J software, and the intensity of the USP7 relative to that of β-actin was further normalized to the ratio in the control treatment. For ubiquitinated proteins from *in vivo* deubiquitination assays, the intensity of ubiquitinated USP7 or ECT2 was quantified by Image J software, and the intensity of the ubiquitinated proteins relative to the intensity of the total precipitated ubiquitins was further normalized to the ratio in the control treatment. For ubiquitinated proteins from *in vitro* deubiquitination assays, the intensity of ubiquitinated USP7 or ECT2 was quantified by Image J software and normalized to that in the control treatment.

### Knockout Cell Generation

USP7 or p53 knockout MCF-7 cells were generated by co-transfection of plasmid encoding FLAG-Cas9 (lentiCas9-Blast) and sgRNA plasmid (lentiGuide-Puro) targeting USP7 (AATCAGATTCAGCATTGCAC) or p53 (ACTTCCTGAAAACAACGTTC). 48 h after transfection, cells were selected by blasticidin (5 μg/mL) and puromycin (1 μg/mL) for 2 days. Pooled clones were used in the corresponding experiments.

### RNA Interference

All siRNA transfections were performed using Lipofectamine RNAiMAX (Thermo Fisher) following the manufacturer's recommendations. The final concentration of the siRNA molecules is 10 nM and cells were harvested 72 or 96 h later according to the purposes of the experiments. Control siRNA (ON-TARGETplus Non-Targeting Pool, D-001810-10), USP7 siRNA (ON-TARGETplus, L-006097-00-0005) and ECT2 siRNA (ON-TARGETplus, LQ-006450-00-0020) were got from Dharmacon in a smart pool manner, while the individual siRNAs against USP7, ECT2, USP11, UHRF1, RAD18, MDM2 or RNF168 were chemically synthesized by Sigma (Shanghai, China). The shRNAs against USP7 or ECT2 were purchased from Sigma. The sequences of siRNAs and shRNAs are provided in Supplementary File 2.

### qRT-PCR

Total cellular RNA was isolated with TRIzol reagent (Thermo Fisher) and used for first strand cDNA synthesis with the Reverse Transcription System (Roche). Quantitation of all gene transcripts was done by qPCR using a Power SYBR Green PCR Master Mix (Roche) and a Lightcycler 480 sequence detection system (Roche) with the expression of *GAPDH* as the internal control. The primers used are listed in Supplementary File 2.

### RNA Sequencing

Total RNA was extracted using TRIzol reagent. The quality of total RNA was checked using the NanoDrop Spectrometer (ND-1000 Spectrophotometer, Peqlab). High quality RNA samples (20 μg each) were sent to Beijing Genomics Institute (BGI, Shenzhen) for cDNA libraries construction and sequencing. The total RNA samples were first treated with DNase I to degrade any possible DNA contamination. Then the mRNA was enriched by using the oligo (dT) magnetic beads followed by fragmentation (about 200 bp). The first strand of cDNA was synthesized by using random hexamer-primer followed by addition of buffer, dNTPs, RNase H and DNA polymerase I to synthesize the second strand. The double-strand cDNA was purified with magnetic beads. End reparation and 3'-end single nucleotide A (adenine) addition was then performed. Finally, sequencing adaptors were ligated to the fragments. The fragments were enriched by PCR amplification. During the QC step, Agilent 2100 Bioanaylzer and ABI Step-One-Plus Real-Time PCR System were used to qualify and quantify of the sample library. The library was loaded onto the channels of an Illumina HiSeq™ 2000 instrument for sequencing. The transcriptome datasets are available at the NCBI Sequence Read Archive (SRA) with accession number SRP181180, the analyzed results with cut off (*p* value ≤ 10^-5^, FDR ≤0.001 and |log_2_ratio| ≥ 1) are provided in Supplementary File 3.

### Lentiviral Production

The shRNAs targeting USP7 or ECT2 or vectors encoding rtTA, USP7 and ECT2, as well as three assistant vectors: pMDLg/pRRE, pRSV-REV, and pVSVG, were transiently transfected into HEK293T cells. Viral supernatants were collected 48 h later, clarified by filtration, and concentrated by ultracentrifugation.

### Immunofluorescence

Cells on glass coverslips (BD Biosciences) were fixed with 4% paraformaldehyde and permeabilized with 0.2% Triton X-100 in PBS. Samples were then blocked in 5% donkey serum in the presence of 0.1% Triton X-100 and stained with the appropriate primary and secondary antibodies coupled to AlexaFluor 488 or AlexaFluor 594 (Thermo Fisher). Confocal images were captured on FluoView1000 Olympus using a × 100 oil objective. To avoid bleed-through effects in double-staining experiments, each dye was scanned independently in a multi-tracking mode.

### Soft Agar Assay

A growth medium solution was prepared by mixing 1.2% agar (Beijing Solarbio Science & Technology) solution with equal volume of 2 × complete DMEM containing 20% serum and added to each well of a 6-well plate to form a basic agar layer. Then, DMEM medium containing transfected cells (usually seeding 20,000 cells in each well of 6-well plate) was mixed 1:1 with 0.6% agar medium solution as the upper agar layer. After 7 days incubation at 37 ºC, the numbers of anchorage-independent tumor cell colonies growing in the soft agar were counted using a phase contrast microscope.

### Nuclei/Cytoplasm Fractionation

MCF-7 cells were harvested with trypsin-EDTA and then centrifuged at 500 g for 5 min. The cell pellet was suspended with ice-cold PBS containing protease inhibitors, and a nuclear/cytosol separation kit (Thermo Scientific) was used to separate the nuclear fraction from the cytoplasmic fraction according to the manufacturer's instructions. The resulting cellular lysates were then boiled in SDS loading buffer and subjected to SDS-PAGE followed by immunoblotting.

### Bioluminescence Resonance Energy Transfer

HEK293T cells were first transfected with control vector and FLAG-ECT2. After 24 h, cells were co-transfected with a constant amount of Rluc-USP7 and increasing amounts of EYFP-USP7. About 48 h after transfection, cells were washed twice with PBS and resuspended in PBS plus 0.1% (w/v) glucose at room temperature. Cells were then distributed in a 96-well microplate and coelenterazine h (Promega) was added at a final concentration of 5 μM. Light emission was collected in a 96-well microplate luminometer for 10 s at 475 nm (Rluc signal) and 535 nm (EYFP signal). The BRET_net_ was calculated based on the formula of BRET_net_ = IA/ID - BRETbkg, where IA represents the intensity of light emission at 535 nm, ID represents the intensity of light emission at 475 nm, and BRETbkg represents the background BRET ratio characterizing Rluc emission in the absence of EYFP.

### Colony Formation Assay

Cells stably expressing indicated genes or/and shRNAs were maintained in culture media for 14 days. After 14 days, the cells were washed with PBS, fixed with methyl alcohol for 10 min and stained with crystal violet (0.5% wt/vol) for 20 min. The number of colonies per well was counted.

### Tissue Specimens

The samples of carcinomas and the adjacent normal tissues were obtained from surgical specimens from patients with breast cancer or others. Samples were frozen in liquid nitrogen immediately after surgical removal and maintained at -80 °C. Prepared tissues were incubated with antibodies against ECT2, USP7 or MDM2 and processed for immunohistochemistry with standard DAB staining protocols. Representative images for normal and malignant breast tumor samples were collected under microscopy. The image quality was evaluated and the background with uneven illumination was corrected with Image-Pro Plus software. Then, the mammary ductal or lobular cells or carcinoma cells were selected as region of interest (ROI) according to morphology features of the tissue or cells. The scores of the stained sections were determined by evaluating the extent and intensity of immunopositivity by Image-pro Plus software. All studies were approved by the Ethics Committee of the Tianjin Medical University, and informed consent was obtained from all patients.

### Tumor Xenografts

MCF-7 cells were plated and infected *in vitro* with lentiviruses carrying control shRNA or ECT2 shRNAs. Then, 4 × 10^6^ viable MCF-7 cells in 200 μL PBS were injected into the mammary fat pads of 6- to 8-week-old athymic female mice (BALB/c; Charles River, Beijing, China). Six animals randomly assigned per group were used in each experiment. Sample size estimate was based on xenograft assays from literatures. 17-β-estradiol (E2) pellets (0.72 mg per pellet, 60 day release; Innovative Research of America, Sarasota, FL) were implanted one day before the tumor cell injection. Similar strategies were used to conduct xenograft experiments with MDA-MB-468 cells, but without pretreatment with 17-β-estradiol (E2) pellets. All animals were sacrificed at the end of the experiment and included into the analysis. The study was approved by the Animal Care Committee of Tianjin Medical University.

### Statistical Analysis

Data from biological triplicate experiments are presented with error bar as mean ± S.D. Two-tailed unpaired Student's t-test was used for comparing two groups of data. Analysis of variance (ANOVA) with Bonferroni's correction was used to compare multiple groups of data. Values that are less than or equal to the first quartile minus 1.5 times the interquartile range, or are greater than or equal to the third quartile plus 1.5 times the interquartile range are defined as outlier ones and indicated with a circle. A *P* value of less than 0.05 was considered significant. All of the statistical testing results were determined by SPSS 20.0 software. Before statistical analysis, variation within each group of data and the assumptions of the tests were checked.

### Data Availability

All relevant data are available from the authors on request. The transcriptome datasets are available at the NCBI Sequence Read Archive (SRA) with accession number SRP181180.

### Study Approval

All procedures involving animals were approved by the Ethics Committee of the Tianjin Medical University and followed the NIH Guide for the Care and Use of Laboratory Animals (8th ed. The National Academies Press. 2011.). All studies associated with patient samples were approved by the Ethics Committee of the Tianjin Medical University, and informed consent was obtained from all patients.

## Results

### A GEF-independent role of ECT2 in breast carcinogenesis

To investigate that whether, and to what extent, ECT2 is involved in breast carcinogenesis, we first analyze the expression of ECT2 in breast cancer with public datasets. Disease analysis from Oncomine displays that the mRNA expression level of *ECT2* is upregulated in breast carcinoma from 12 out of 43 analyses in 4 of 10 datasets 53, and *ECT2* is highly expressed in distinct histological breast cancer samples (Figure S1A). Meanwhile, we showed the expression level of ECT2 appears to be identical among different molecular subtypes of breast carcinoma, especially for luminal B, Her2-enriched and basal like ones, with datasets from Gene Expression Omnibus (GEO) (Figure S1B). Consistent with previous report 34, these results suggest that ECT2 is implicated in breast cancer. However, it appears that dysregulation of ECT2 is commonly involved in the development of breast cancer without histological or molecular subtype preference.

To confirm the involvement of ECT2 in breast carcinogenesis, we next analyzed, by immunohistochemical (IHC) staining, the expression profiles of ECT2 with samples from different histologic grade breast carcinoma and histologically normal mammary tissues in tumor adjacent regions. Quantitative analysis of the stainings showed that ECT2 is highly expressed in breast carcinoma and the level of ECT2 expression largely correlates with tumor grade (Figure S1C). In order to gain further support of the role of ECT2 in breast cancer progression and to extend our observations to a clinicopathologically relevant setting, we analyzed the expression of ECT2 and its correlation with clinical behaviors of breast cancer patients. Kaplan-Meier survival analysis from GEO datasets indicates that elevated *ECT2* expression predicts poor overall survival (OS), relapse free survival (RFS), distant metastasis free survival (DMFS), and post progression survival (PPS) in patients suffered from breast cancer (Figure S1D). Together, these findings point to a tumor-promoting role for ECT2 in breast cancer.

**Figure 1:**
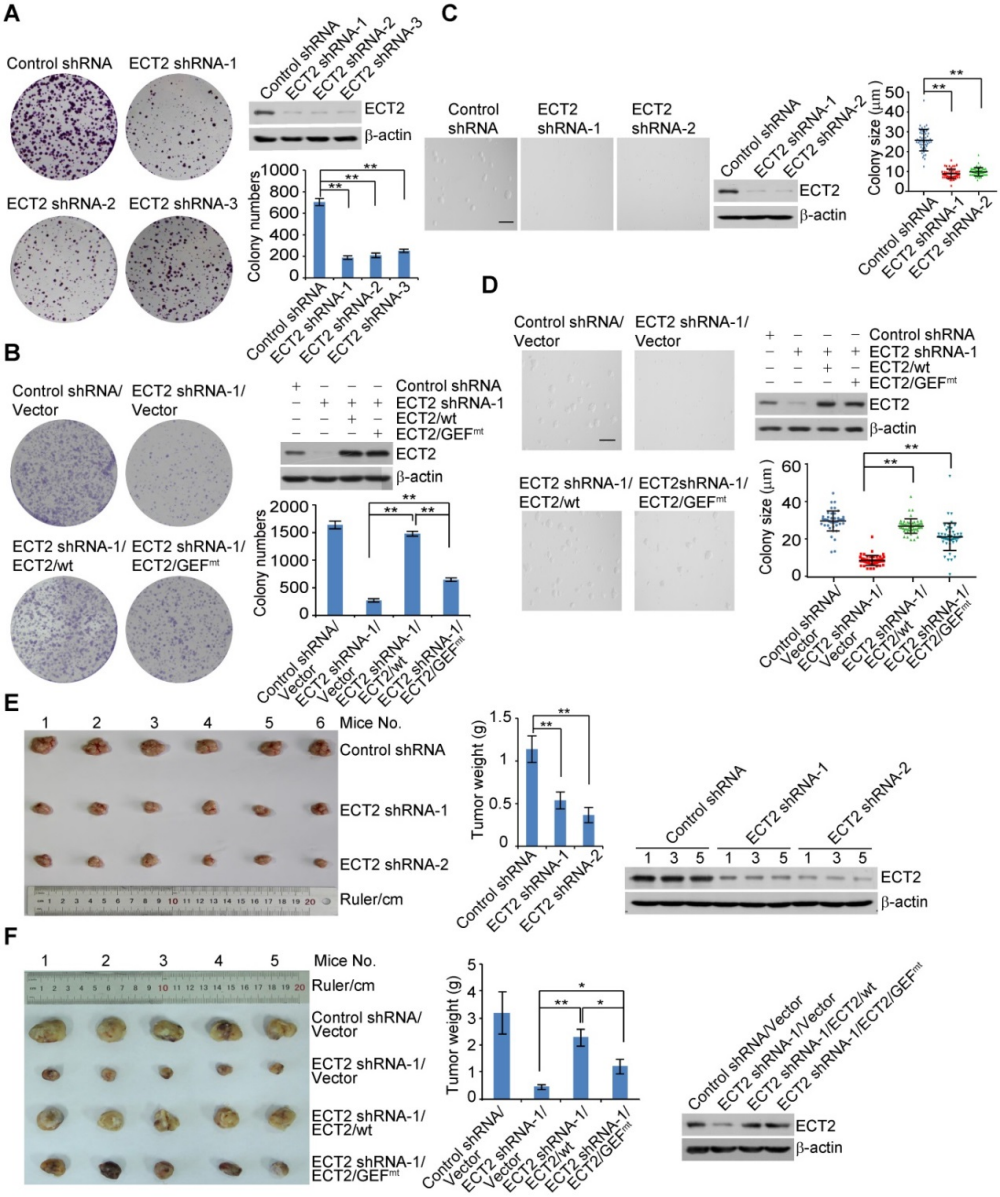
** ECT2 Deregulation Contributes to Breast Carcinogenesis.** (A) Colony formation assays were conducted with MCF-7 cells stably expressing the shRNA targeting 5'UTR (shRNA-1) or CDS (shRNA-2 and shRNA-3) region of ECT2. Representative images from biological triplicate experiments are shown. Colony numbers were counted and statistically analyzed. Each bar represents the mean ± S.D. **P < 0.01, one-way ANOVA. The expression of ECT2 was examined by Western blotting. (B) Colony formation assays were conducted with MCF-7 cells stably expressing the shRNA targeting 5'UTR (shRNA-1) and FLAG tagged wild type ECT2 (ECT2/wt) or GEF activity-deficient ECT2 mutant (ECT2/GEF^mt^). Representative images from biological triplicate experiments are shown. Colony numbers were counted and statistically analyzed. Each bar represents the mean ± S.D. **P < 0.01, one-way ANOVA. The expression of ECT2 was examined by Western blotting. (C) Soft agar assays to assess anchorage-independent growth were conducted with MCF-7 cells stably expressing the indicated shRNAs. Representative images from biological triplicate experiments are shown. Colon size (diameter) was counted and statistically analyzed. Each bar represents the mean ± S.D. **P < 0.01, one-way ANOVA. The expression of ECT2 was examined by Western blotting. Scale bar, 100 μm. (D) Soft agar assays to assess anchorage-independent growth were conducted with MCF-7 cells stably expressing the indicated shRNAs and genes. Representative images from biological triplicate experiments are shown. Colon size (diameter) was counted and statistically analyzed. Each bar represents the mean ± S.D. **P < 0.01, one-way ANOVA. The expression of ECT2 was examined by Western blotting. Scale bar, 100 μm. (E) Control or ECT2-deficient MCF-7 tumors were transplanted onto athymic mice and tumors were harvested 8 weeks later. Each bar represents the mean ± S.D. for tumor weight measurements (n = 6, in each group). ^**^P < 0.01*,* one-way ANOVA. The levels of indicated proteins in the representative tumors were examined by Western blotting. (F) Distinct MDA-MB-468 tumors as indicated were transplanted onto athymic mice and tumors were harvested 8 weeks later. Each bar represents the mean ± S.D. for tumor weight measurements (n = 5, in each group). ^*^P < 0.05, ^**^P < 0.01*,* one-way ANOVA. The levels of indicated proteins in the representative tumors were examined by Western blotting.

To understand the role of ECT2 in breast carcinogenesis, we first analyzed the effect of ECT2 depletion on breast cancer cell survival. Colony formation assays showed that knockdown of ECT2 severely impeded the colony size and numbers of breast cancer cells including MCF-7 cells, ZR-75-1 cells, and MDA-MB-468 cells (Figure 1A, 1B, S1E, and S1F). To investigate whether GEF activity of ECT2 is essential for tumor survival, control shRNA or shRNA targeting the 5'UTR region of *ECT2* was stably integrated into breast cancer cells stably expressing wild type ECT2 (ECT2/wt) or GEF activity-deficient ECT2 mutant (ECT2/GEF^mt^, E428A and N608A within the DH domain) 15, 54. Colony formation assays demonstrated that ECT2/GEF^mt^ could, to a measurable extent, offset the growth defect induced by ECT2 depletion (Figure 1B, S1E, and S1F). Similar results were obtained when anchorage-independent growth assays were performed with MCF-7 cells (Figure 1C and 1D). These results suggest that activities other than GEF likely play a role in ECT2-promoted tumor cell survival.

To consolidate the role of ECT2 in breast carcinogenesis, we transplanted control or ECT2-deficient MCF-7 cells onto the mammary fat pads of athymic mice (BALB/c; Charles River Laboratories). Tumor growth and mouse weight were monitored over 8 weeks. Notably, the tumor growth was greatly suppressed in athymic mice that received tumor transplants with ECT2-depleted cells (Figure 1E). The expression of ECT2 was verified by Western blotting in the harvested tumors (Figure 1E). To further investigate whether GEF-independent activity of ECT2 contributes to breast tumor growth, MDA-MB-468 cells stably expressing ECT2 shRNA and ECT2/wt or ECT2/GEF^mt^ were created, and the tumor formation of these cells were examined via xenograft model. We found that tumor growth retardation resulted from ECT2 depletion could be largely reverted by ECT2/wt, while ECT2/GEF^mt^ also showed a detectable compensation effect, albeit not as effective as ECT2/wt (Figure 1F). Collectively, these results support the notion that a GEF-independent role of ECT2 is involved in breast carcinogenesis.

### ECT2 is physically associated with the Ubiquitin-Specific Protease USP7

**Figure 2:**
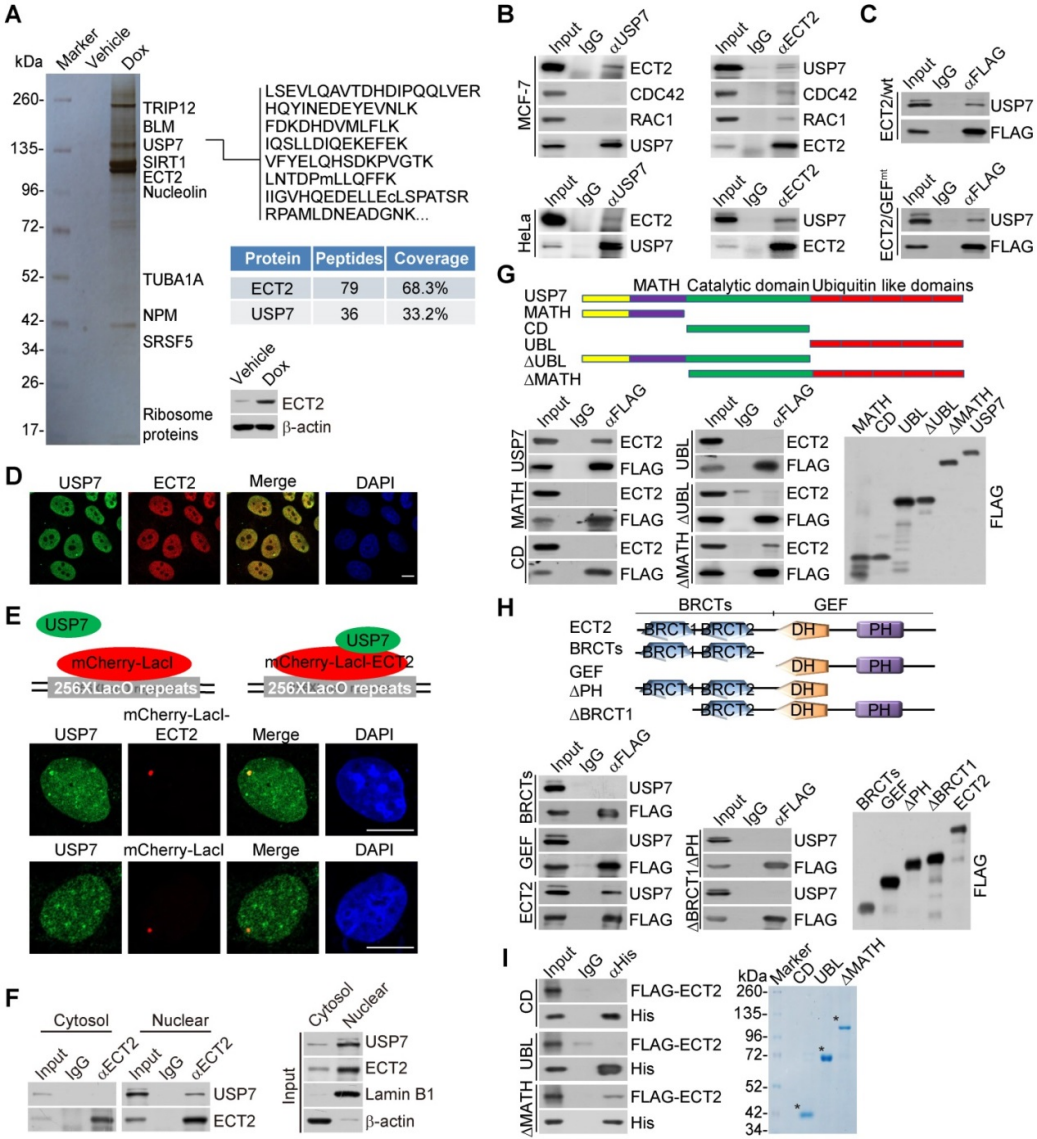
** ECT2 Is Physically Associated with USP7.** (A) Immunoaffinity purification of ECT2-containing protein complexes. Whole-cell extracts from MCF-7 cells with doxycycline (Dox)-inducible expression of stably integrated FLAG-ECT2 were purified with an anti-FLAG affinity column. After extensive washing, the bound proteins were eluted with excess FLAG peptides, resolved, and visualized by silver staining on SDS-PAGE. The protein bands on the gel were recovered and analyzed by mass spectrometry. Representative peptide fragments of USP7 and peptide coverage of the indicated proteins are shown. Detailed results from the mass spectrometric analysis are provided as Supplementary File 1. The expression of ECT2 in these cells was examined by Western blotting. (B) Co-immunoprecipitation analysis of the association between ECT2 and USP7. Whole cell lysates from HeLa cells and MCF-7 cells were immunoprecipitated (IP) and then immunoblotted (IB) with antibodies against the indicated proteins. (C) Whole cell lysates from HeLa cells transfected with wild type ECT2 (ECT2/wt) or mutant ECT2 carrying E428A and N608A (ECT2/GEF^mt^) were immunoprecipitated and then immunoblotted with antibodies against the indicated proteins. (D) MCF-7 cells were fixed and immunostained with antibodies against ECT2 and USP7 followed by confocal microscopy analysis. Scale bar, 10 μm. (E) U2OS cells carrying an array of LacO operators were co-transfected with mCherry-LacI-ECT2 or mCherry-LacI. Cells were immunostained with USP7 antibody and visualized using confocal microscopy. Scale bar, 10 μm. (F) Co-immunoprecipitation analysis of the interaction between ECT2 and USP7 with cellular lysates from different cellular compartments of MCF-7 cells. (G) Co-immunoprecipitation analysis of the association between ECT2 and USP7 or USP7 mutants. FLAG-tagged deletion mutants of USP7 were transfected into HeLa cells followed by co-immunoprecipitation analysis. MATH, the meprin and tumor necrosis factor-receptor associated factor (TRAF) homology domain; CD, catalytic domain; UBL, ubiquitin like domain. (H) Co-immunoprecipitation analysis of the association between USP7 and ECT2 or ECT2 mutants. FLAG-tagged deletion mutants of ECT2 were transfected into HeLa cells followed by co-immunoprecipitation analysis. BRCT, BRCA1 C-terminal; GEF (containing DH and PH domain), guanine nucleotide exchange factor; DH, Dbl homology; PH, pleckstrin homology. (I) His-pull down assays with deletion mutants of USP7 purified from Sf9 cells and *in vitro* transcribed/translated ECT2/wt. The asterisk indicates the recombinant protein stained by Commassie Blue.

To understand the molecular mechanisms of how ECT2 contributes to breast carcinogenesis, we then employed affinity purification and mass spectrometry to interrogate ECT2 interactome *in vivo*. We generated a mammary carcinoma MCF-7 cell line that allows doxycycline (Dox)-inducible expression of stably integrated FLAG-ECT2 (Figure 2A). Mass spectrometry analysis of FLAG-ECT2 containing protein complex revealed that ECT2 was associated with a number of proteins, including SIRT1, TRIP12, and NPM (Figure 2A and Supplementary File 1). Interestingly, USP7, a member of the protein deubiquitinases with oncogenic activity 42, 48, 55-57, was also identified (Figure 2A).

To confirm the *in vivo* association of ECT2 with USP7, co-immunoprecipitation experiments were performed with cellular extracts from MCF-7 cells. Immunoprecipitation (IP) with antibodies against USP7 followed by immunoblotting (IB) with antibodies against ECT2 demonstrated that ECT2 was efficiently co-immunoprecipitated with USP7 (Figure 2B). Reciprocally, IP with antibodies against ECT2 and IB with antibodies against USP7 also revealed that USP7 was efficiently co-immunoprecipitated with ECT2 (Figure 2B). Similar results were obtained when the association between USP7 and ECT2 was examined with cellular extracts from HeLa cells (Figure 2B). Interestingly, we found that RAC1 and CDC42 could interact with ECT2, but not USP7 (Figure 2B), implying that ECT2 and USP7 form a distinct complex in the absence of these Rho-GTPases. Consistently, we also revealed that USP7 could be co-immunoprecipitated with ECT2/GEF^mt^, and this mutant exhibited similar binding affinity to USP7 as wild type ECT2 (ECT2/wt) (Figure 2C).

Next, immunofluorescent staining followed by confocal microscopy analysis revealed that ECT2 is co-localized with USP7 (Figure 2D). Furthermore, we tested whether USP7 co-localized with ECT2 using the LacO-LacI targeting system. In this system, multiple LacO repeats are stably integrated into genome 58, and LacI fusion proteins could be efficiently recruited and concentrated to LacO arrays on targeted chromatin. We observed that endogenous USP7 formed evident foci that co-localized with ECT2-mCherry-LacI, whereas USP7 did not form foci in cells expressing mCherry-LacI (Figure 2E). In addition, co-immunoprecipitation analysis with protein fractionations from different cellular compartments indicated that USP7 could be co-immunoprecipitated with nuclear, but not cytosolic, ECT2 (Figure 2F). These results suggested that nuclear ECT2 is physically associated with USP7, and possibly the molecular behavior of ECT2 in this protein complex is irrelevant to the canonical GEF-GTPase signal.

To further consolidate the interaction between ECT2 and USP7 and to gain insights into the molecular detail involved in the interaction between these two proteins, FLAG-tagged domain deletion mutants of USP7 were generated and transfected into HeLa cells. Co-immunoprecipitation analysis demonstrated that neither the separately expressed MATH domain in the N terminus, nor CD domain in the middle, or ubiquitin like (UBL) domain in the C terminus of USP7 was responsible for the interaction of USP7 with ECT2 (Figure 2G). Interestingly, only MATH domain deficient USP7 (USP7/ΔMATH) containing both CD and UBL domains, was able to interact with ECT2, suggesting that higher structure formed by CD and UBL domains as a whole is required for the molecular interface connection between USP7 and ECT2 (Figure 2G). Similarly, domain mapping of the molecular interface of ECT2 required for USP7 binding revealed that USP7 could be only co-immunoprecipitated with the full length ECT2, but not the N-terminus BRCT domains or the C-terminus GEF (containing DH and PH) domain alone (Figure 2H). Furthermore, *in vitro* pull-down experiments with Sf9 cells-purified His-tagged USP7 truncation mutants and ECT2 that was transcribed/translated *in vitro* demonstrated that ECT2 was capable of interacting with USP7/ΔMATH (Figure 2I). Collectively, these results indicated that the binding of ECT2 to USP7 is dependent on the higher-ordered structure of these proteins.

### ECT2 antagonizes USP7 polyubiquitination and promotes its stabilization

**Figure 3:**
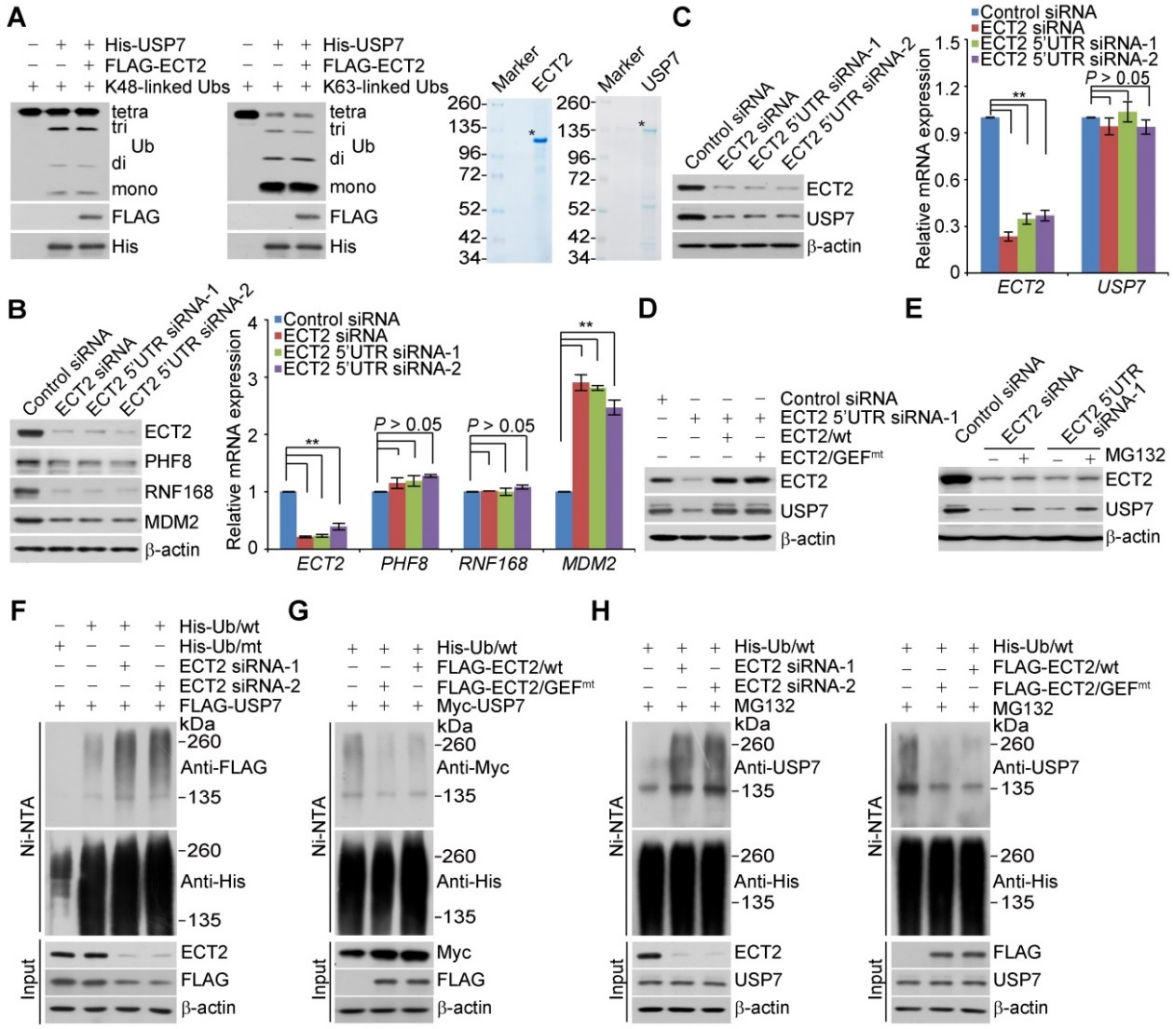
** ECT2 Prevents USP7 Degradation through Opposing Its Polyubiquitination.** (A) *In vitro* deubiquitination assays with K48- or K63-linked tetra-ubiquitins (0.5 μg) and Sf9 cells-purified recombinant USP7 (1.5 μg) in the presence or absence of ECT2 purified from HeLa cells with high salt and detergent buffer. After 4 h of incubation, the cleavage effect was examined by Western blotting with antibody against ubiquitin. The asterisk indicates the recombinant protein stained by Commassie Blue. (B) MCF-7 cells were transfected with control siRNA or different sets of ECT2 siRNAs. Cellular extracts and total RNA were prepared and analyzed by Western blotting and qRT (quantitative reverse transcription)-PCR, respectively. Each bar represents the mean ± S.D. for biological triplicate experiments.^ **^P < 0.01*,* one-way ANOVA. (C) MCF-7 cells were transfected with control siRNA or different sets of ECT2 siRNAs. Cellular extracts and total RNA were prepared and analyzed by Western blotting and qRT-PCR, respectively. Each bar represents the mean ± S.D. for biological triplicate experiments. ^**^P < 0.01, one-way ANOVA. (D) MCF-7 cells were transfected with siRNAs targeting 5'UTR of ECT2 and ECT2/wt or ECT2/GEF^mt^. Cellular extracts were prepared and analyzed by Western blotting. (E) MCF-7 cells were transfected with control siRNA or ECT2 siRNAs followed by treatment with DMSO or proteasome inhibitor MG132 (10 μM for 4 h). Cellular extracts were prepared and analyzed by Western blotting. (F) HeLa cells stably expressing FLAG-USP7 were co-transfected with His-tagged wild-type ubiquitin (Ub/wt) or a ubiquitin mutant (Ub/mt) with all lysine residues replaced by arginine and control siRNA or different sets of ECT2 siRNAs. Cellular extracts were prepared for affinity-based precipitation assays with Ni-NTA agarose beads under the denature condition followed by immunoblotting analysis. (G) HeLa cells stably expressing Myc-USP7 were then co-transfected with His-tagged Ub/wt and FLAG-ECT2/wt or ECT2/GEF^mt^. Cellular extracts were prepared for affinity-based precipitation assays with Ni-NTA agarose beads followed by immunoblotting analysis. (H) MCF-7 cells were co-transfected with siRNAs or/and genes as indicated. Cells were pretreated with MG132 (10 μM) for 10 h before collection, and cellular extracts were prepared for affinity-based precipitation assays with Ni-NTA agarose beads followed by immunoblotting analysis.

The C-terminal UBL domain of USP7 is essential for turning on the catalytic activity of USP7 and this effect can be further allosterically potentiated by GMPS 45, 59. Considering that the association of USP7 with ECT2 also requires the UBL domain, we wondered whether ECT2 could allosterically enhance the deubiquitinase activity of USP7. To test this hypothesis, *in vitro* deubiquitination assays were performed with K48- or K63- linked ubiquitin linkages and insect cells expressed USP7, in the absence or presence of ECT2 purified from mammalian cells. The results showed that recombinant USP7 is capable of cleaving ubiquitin chains with an evident accumulation of lower molecular weight of ubiquitin conjugates, while addition of ECT2 has marginal effect on USP7-catalyzed cleavage of ubiquitin chains (Figure 3A).

Although ECT2 fails to activate the enzymatic activity of USP7, we surprisingly found that ECT2 depletion resulted in markedly downregulation of several USP7 substrates including PHF8 42, RNF168 60, and MDM2 50 at protein, but not mRNA level (Figure 3B). This prompted us to further examine whether the expression of USP7 itself is controlled by ECT2. Interestingly, we found that the protein level of USP7 was significantly reduced in ECT2-deficient MCF-7 (Figure 3C), ZR-75-1 (Figure S2A) and MDA-MB-468 cells (Figure S2B), whereas USP7 mRNA level was essentially unchanged (Figure 3C, S2A and S2B). Next, MCF-7 cells were transfected with FLAG-ECT2 and siRNA targeting 5'UTR of ECT2. Western blotting analysis revealed that gain of expression of ECT2 was able to restore the expression of USP7 in ECT2 deficient cells (Figure 3D). Meanwhile, overexpression of ECT2/GEF^mt^ also resulted in an elevated expression of USP7 (Figure 3D). Moreover, we found that loss of CDC42 or RAC1, either individually or together, had no effect on the expression of USP7 (Figure S2C). These observations implied that ECT2 controls USP7 stabilization in a GEF activity-independent manner. Next, addition of MG132, a proteasome specific inhibitor, effectively blocked the reduction of USP7 associated with ECT2 depletion, suggesting that this effect was probably through a proteasome-mediated protein degradation mechanism (Figure 3E). Furthermore, we measured the half-life of USP7 in the presence and absence of ECT2 by cycloheximide chase assays (CHX). Western blotting analysis revealed that USP7 showed a decreased half-life when ECT2 was deficient (Figures S2D). Collectively, these results suggested that ECT2 controls the stability of USP7 and the nuclear GEF/GTPase signaling is not engaged into this process.

To test whether ECT2-promoted USP7 stabilization is through opposing ubiquitination-mediated proteasome degradation, we next examined whether ECT2 functions to antagonize USP7 ubiquitination. HeLa cells stably expressing FLAG-USP7 were co-transfected with His-tagged wild-type ubiquitin (Ub/wt) or a ubiquitin mutant (Ub/mt) with all lysine residues replaced by arginine and control siRNA or different sets of ECT2 siRNAs. IP of the cellular lysates with Ni-NTA agarose beads followed by IB with anti-FLAG showed that knockdown of ECT2 resulted in an increase in the level of ubiquitinated USP7 species (Figure 3F and S2E). Next, immunoprecipitation analysis revealed that the level of ubiquitinated Myc-USP7 species was evidently decreased in ECT2 highly expressing cells (Figure 3G and S2F). Consistently, we demonstrated that the ubiquitination level of endogenous USP7 was elevated in ECT2-deficient cells and reduced in ECT2-proficient cells (Figure 3H and S2G). Furthermore, we revealed that ECT2/GEF^mt^ promotes USP7 deubiquitination as efficiently as ECT2/wt does (Figure 3G and 3H; Figure S2F and S2G). Taken together, these results suggested that ECT2 counteracts the polyubiquitination of USP7 thus promotes USP7 stabilization in a GEF activity-independent manner.

### ECT2 promotes USP7 intermolecular self-association, -deubiquitination and -stabilization

Since ECT2 could not allosterically activate USP7 and it possesses neither E3 ligase nor deubiquitinase activity, we hypothesized that ECT2-promoted USP7 stabilization is likely through enhancing the association of USP7 with other deubiquitinase(s) or protecting it from ubiquitination by E3 ligase(s). Indeed, many interactors of USP7 act as ubiquitin processing enzymes, such as USP11 61, UHRF1 62-64, RAD18 65, MDM2 50 and RNF168 60. However, individual depletion of these enzymes had little effect on the expression of USP7 (Figure S3A). These observations implied that ECT2 may take advantage of other molecular approaches to control the stabilization of USP7.

**Figure 4:**
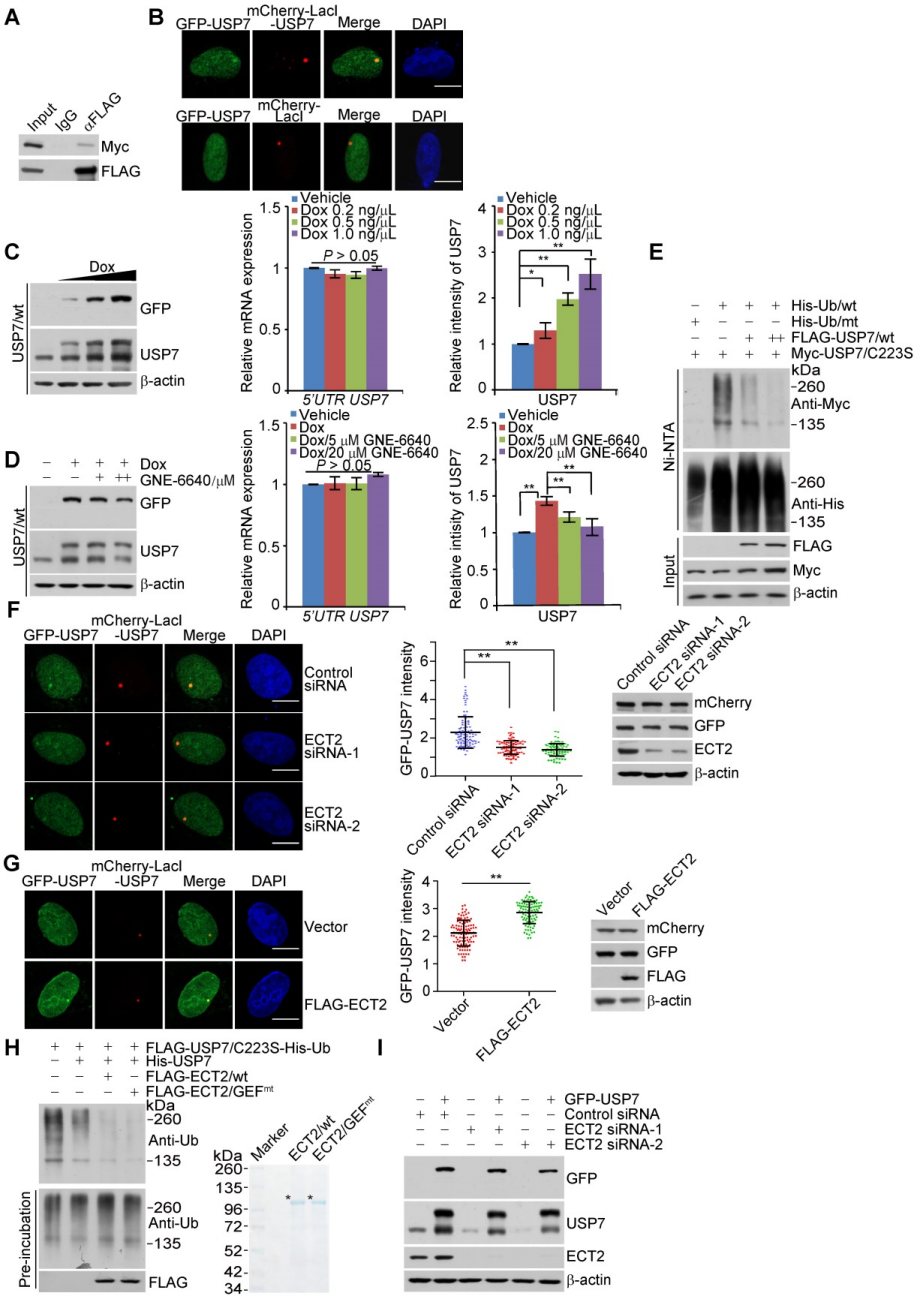
** ECT2 Facilitates USP7 Intermolecular Self-association, -Deubiquitination and -Stabilization.** (A) Co-immunoprecipitation analysis with cellular extracts from HeLa cells expressing Myc-USP7 and FLAG-USP7. (B) U2OS cells carrying an array of LacO operators were co-transfected with GFP-USP7 and mCherry-LacI-USP7 or mCherry-LacI. Scale bar, 10 μm. (C) MCF-7 cells allowing Dox-inducible expression of stably integrated GFP-USP7/wt were cultured in the presence of increasing amounts of doxycycline. Cellular extracts and total RNA were prepared and analyzed by Western blotting and qRT-PCR, respectively. For USP7 bands, the higher one with larger molecular weight represents GFP-tagged USP7, while the lower one indicates endogenous USP7. The quantitation of endogenous USP7 was shown. Each bar represents the mean ± S.D. for biological triplicate experiments.^ *^P < 0.05, ^**^P < 0.01, one-way ANOVA. (D) MCF-7 cells allowing Dox-inducible expression of stably integrated GFP-USP7/wt were cultured in the absence or presence of USP7 inhibitor GNE-6640 for 24 h. Cellular extracts and total RNA were prepared and analyzed by Western blotting and qRT-PCR, respectively. For USP7 bands, the higher one with larger molecular weight represents GFP-tagged USP7, while the lower one indicates endogenous USP7. The quantitation of endogenous USP7 was shown. Each bar represents the mean ± S.D. for biological triplicate experiments.^ **^P < 0.01, one-way ANOVA. (E) HeLa cells stably expressing Myc-USP7/C223S were co-transfected with control vector or FLAG-USP7/wt and His-Ub/wt or His-Ub/mt. Cellular extracts were prepared for affinity-based precipitation assays with Ni-NTA agarose beads followed by immunoblotting analysis. (F) LacO cells expressing GFP-USP7 and mCherry-LacI-USP7 were transfected with control siRNA or ECT2 5'UTR siRNA. The intensity of GFP-USP7 foci was quantified and normalized against nuclear dispersed GFP-USP7, and more than 50 nuclei from biological triplicate experiments were used for quantification. Scale bar, 10 μm. ^**^P < 0.01*,* one-way ANOVA. The expression of indicated protein was examined by Western blotting. (G) LacO cells expressing GFP-USP7 and mCherry-LacI-USP7 were transfected with control vector or FLAG-ECT2. The intensity of GFP-USP7 foci was quantified and normalized against nuclear dispersed GFP-USP7, and more than 50 nuclei from biological triplicate experiments were used for quantification. Scale bar, 10 μm. ^**^P < 0.01*,* two-tailed unpaired Student's t-test. The expression of indicated protein was examined by Western blotting. (H) *In vitro* deubiquitination assays with Sf9 cells-purified USP7 (2 μg) and His-Ub conjugated USP7/C223S purified from HeLa cells by high salt and denature buffer with Ni-NTA agarose beads in the absence or presence of HeLa cells-purified ECT2/wt or ECT2/GEF^mt^ (2 μg) The asterisk indicates the recombinant protein stained by Commassie Blue. (I) MCF-7 cells allowing Dox-inducible expression of stably integrated GFP-USP7 were transfected with control siRNA or ECT2 siRNAs and then cultured in the absence or presence of doxycycline. Cellular extracts were prepared and analyzed by Western blotting.

As deubiquitinases USP4 66, 67, USP19 68, and USP7 itself 51, 69 are reported to exist in a dimeric or oligomeric state and USP19 stabilizes itself through self-association 68, we wondered whether USP7 acts in the same manner as USP19, and, importantly, whether ECT2 plays a role in this process. Consistent with previous reports 51, 69, we first verified that USP7 is able to interact with itself, manifested as that Myc-USP7 could be efficiently immunoprecipitated by FLAG-USP7 (Figure 4A). Meanwhile, we revealed that a fraction of GFP-USP7 is recruited to mCherry-LacI-USP7, but not mCherry-LacI, nucleated chromatin regions in LacO cells, implying a self-association of USP7 (Figure 4B).

To test the functional significance of this dimeric/oligomeric form of USP7, we created MCF-7 cells that allow Dox-inducible expression of stably integrated GFP-USP7/wt or GFP-USP7/C223S, a catalytic mutant of USP7, the band of which could be discernably separated from endogenous USP7 on SDS-PAGE gel (Figure 4C, 4D, S3B and S3C). Western blotting analysis indicated that Dox treatment resulted in a dose-dependent increase at the protein level of endogenous USP7 in GFP-USP7/wt stably expressing cells, while the mRNA expression of endogenous USP7 was not altered as examined by qRT-PCR with primers covering the UTR region of USP7 (Figure 4C). Importantly, treatment of cells for 24 h with USP7 inhibitor GNE-6640, which selectively compete with K48-linked ubiquitin chains for binding to USP7 56, significantly impaired this effect (Figure 4D). In contrast, USP7/C223S overexpression has little effect on the expression of endogenous USP7, although GNE-6640 treatment still led to downregulation of USP7 (Figure S3B and S3C). These results suggested that USP7 interacts with and stabilizes itself. Next, we examined whether USP7 controls its own ubiquitination. Indeed, the catalytic mutant of USP7 (USP7/C223S) displayed a higher level of polyubiquitination than wild type USP7 (USP7/wt) (Figure S3D). Moreover, forced expression of USP7/wt dramatically decreased the level of ubiquitinated USP7/C223S species (Figure 4E and S3E). Therefore, USP7 likely removes polyubiquitin conjugates from itself to maintain its protein abundance. The activity of USP7 towards its polyubiquinated species is consistent with the observation that it is capable of cleaving monoubiquitination on itself 70.

To investigate the role of ECT2 in USP7 self-regulation, we first transfected control siRNA or ECT2 siRNA into LacO cells expressing GFP-USP7 and mCherry-LacI-USP7. Confocal microscopy analysis demonstrated that ECT2 depletion significantly compromised the co-localization of GFP-USP7 and mCherry-LacI-USP7 (Figure 4F). Additionally, we showed ECT2 overexpression promotes this effect (Figure 4G). Next, energy transfer analysis with cells expressing Rluc-USP7 and EYFP-USP7 monitored by BRET (Bioluminescence Resonance Energy Transfer) assay further suggested that ECT2 could prompt oligomerization of USP7, manifested by an increase of energy transfer from Rluc-USP7 to EYFP in the presence of over-expressed ECT2 (Figure S3F). These results indicated that ECT2 plays a role of importance in promoting USP7 self-association. Consistent with the results from *in vivo* deubiquitination assay (Figure 3G and 3H), *in vitro* deubiquitination assay with Sf9 cells-purified USP7 and His-Ub conjugated USP7/C223S purified from HeLa cells by high salt and denature buffer demonstrated that ECT2 addition markedly promoted USP7 self-deubiquitination (Figure 4H and S3G). Meanwhile, we revealed that ECT2/GEF^mt^ behaves similarly as ECT2/wt in modulating USP7 self-deubiquitination (Figure 4H and S3G). Furthermore, we revealed that ECT2 depletion impaired the upregulation of endogenous USP7 induced by forced expression of GFP-USP7 (Figure 4I and S3H). In order to confirm this observation, we used another commercial antibody against USP7 to detect the alteration of USP7 expression (Figure S3I). Collectively, these results suggested that ECT2 could promote USP7 intermolecular self-association, -deubiquitination and -stabilization, and these effects are independent of ECT2 GEF activity.

### USP7 stabilizes ECT2

**Figure 5:**
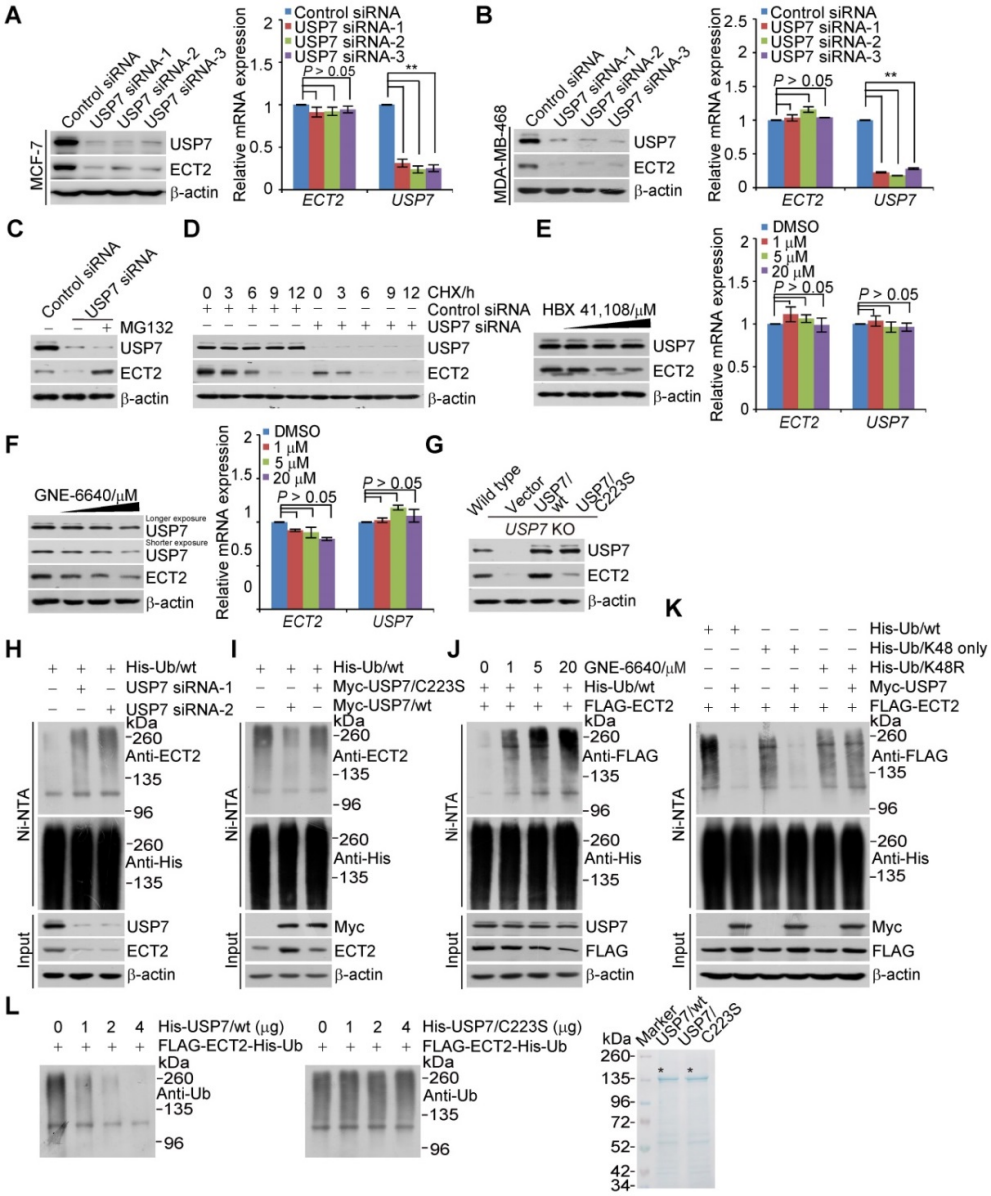
** USP7 Promotes ECT2 Stabilization and Deubiquitination.** (A) MCF-7 cells were transfected with control siRNA or different sets of USP7 siRNAs. Cellular extracts and total RNA were prepared and analyzed by Western blotting and qRT-PCR, respectively. Each bar represents the mean ± S.D. for biological triplicate experiments.^ **^P < 0.01, one-way ANOVA. (B) MDA-MB-468 cells were transfected with control siRNA or different sets of USP7 siRNAs. Cellular extracts and total RNA were prepared and analyzed by Western blotting and qRT-PCR, respectively. Each bar represents the mean ± S.D. for biological triplicate experiments.^ **^P < 0.01, one-way ANOVA. (C) MCF-7 cells were transfected with control siRNA or USP7 siRNA followed by treatment with DMSO or proteasome inhibitor MG132 (10 μM, 4 h). Cellular extracts were prepared and analyzed by Western blotting. (D) MCF-7 cells transfected with control siRNA or USP7 siRNA were treated with 50 μg/mL cycloheximide (CHX) and harvested at the indicated time followed by Western blotting analysis. (E) MCF-7 cells were cultured in the absence or presence of HBX 41,108 for 4 h, as indicated. Cellular extracts and total RNA were collected and analyzed by Western blotting and qRT-PCR, respectively. Each bar represents the mean ± S.D. for biological triplicate experiments. *P* values were determined by one-way ANOVA. (F) MCF-7 cells were cultured in the absence or presence of GNE-6640 for 24 h, as indicated. Cellular extracts and total RNA were collected and analyzed by Western blotting and qRT-PCR, respectively. Each bar represents the mean ± S.D. for biological triplicate experiments. *P* values were determined by one-way ANOVA. (G) CRISPR/Cas9 generated* USP7* knockout MCF-7 cells were transfected with control vector, USP7/wt, or USP7/C223S. Cellular extracts were prepared and analyzed by Western blotting. (H) MCF-7 cells were co-transfected with USP7 siRNAs and Ub/wt. Cellular extracts were prepared for affinity-based precipitation assays with Ni-NTA agarose beads followed by immunoblotting analysis. (I) MCF-7 cells stably expressing USP7/wt or USP7/C223S were transfected with Ub/wt. Cellular extracts were prepared for affinity-based precipitation assays with Ni-NTA agarose beads followed by immunoblotting analysis. (J) HeLa cells stably expressing FLAG-ECT2 were transfected with His-Ub/wt and cultured in the absence or presence of GNE-6640 for 4 h. Cellular extracts were prepared for affinity-based precipitation assays with Ni-NTA agarose beads followed by immunoblotting analysis. (K) HeLa cells stably expressing FLAG-ECT2 were co-transfected with His-Ub/K48-only or His-Ub/K48R and Myc-USP7 followed by affinity-based precipitation and immunoblotting analysis. (L) *In vitro* deubiquitination assays with His-Ub conjugated ECT2 purified from HeLa cells using high salt and denature buffer and USP7/wt or USP7/C223S purified from Sf9 cells with indicated amounts. The asterisk indicates the recombinant protein stained by Commassie Blue.

To further address the functional significance of the physical interaction between USP7 and ECT2, we examined the effect of USP7 on the expression of ECT2. Western blotting analysis revealed that the level of ECT2 was significantly reduced in USP7-depleted MCF-7 cells and MDA-MB-468 cells (Figure 5A and 5B), whereas the mRNA expression level of ECT2 was not affected upon USP7 depletion (Figure 5A and 5B). Moreover, the reduction in ECT2 protein level associated with USP7 depletion was probably a result of proteasome-mediated protein degradation, as the effect could be effectively blocked by MG132 (Figure 5C). To further support this deduction, the potential of USP7 to modulate the steady-state level of ECT2 protein was assessed by cycloheximide (CHX) chase assays. In these experiments, MCF-7 cells transfected with control siRNA or USP7 siRNA were incubated with CHX and harvested at different time points. Western blotting analysis revealed that USP7 depletion was clearly associated with a decreased half-life of ECT2 (Figure 5D). These observations indicated that ECT2 is a potential substrate of USP7.

To gain molecular insights into the functional connection between USP7 and ECT2, we examined whether USP7-promoted ECT2 stabilization is dependent on its ubiquitin-specific protease activity. First, treatment of MCF-7 cells with HBX 41,108 for 4 h, a cyanoindenopyrazine-derived deubiquitinase inhibitor known to inhibit catalytic activity of USP7, resulted in a dose-dependent reduction in the protein level of ECT2, while it had no effect on its mRNA level (Figure 5E). Similar results were obtained with USP7 inhibitor GNE-6640. The abundance of USP7 itself was also downregulated, when cells were cultured with this inhibitor for 24 h (Figure 5F). Furthermore, the downregulation of ECT2 in USP7-knockout cells could be fully reverted by forced expression of USP7/wt, but not USP7/C223S (Figure 5G). Together, these results support the argument that USP7 regulates the stability of ECT2 through USP7 deubiquitinase activity.

Given that ECT2 is an ubiquitinated protein 24, 71 and USP7 promotes the stabilization of ECT2, we next asked the question whether USP7 functions to deubiquitinate ECT2. First, we showed that knockdown of USP7 resulted in an increase in the level of ubiquitinated ECT2 species (Figure 5H and S4A). Then, we found that USP7 overexpression was associated with a decreased level of ubiquitinated ECT2 species (Figure 5I and S4B). Interestingly, *in vivo* deubiquitination assays showed that USP7/C223S was also able to cleave ubiquitinated ECT2 species, albeit less profoundly than USP7/wt (Figure 5I and S4B). This effect could be largely due to heterogeneous oligomerization of the exogenous USP7/C223S and endogenous USP7, which possibly in turn favors ECT2 deubiquitination, thus stabilization. Consistent with the observation that enzymatic inhibition of USP7 resulted in ECT2 destabilization, treatment of HeLa cells with USP7 inhibitor GNE-6640 for 4 h resulted in a marked increase in the level of ubiquitinated ECT2 species and a decrease of total ECT2 (Figure 5J and S4C). Yet, USP7 inhibitor treatment for 4 h had marginal effect on the level of USP7 (Figure 5E and 5J). This is possibly due to the half-life time of USP7 is extremely longer than that of ECT2 (Figure 5D and Figure S2D).

Next, we utilized ubiquitin mutant with all lysine residues replaced by arginine except K48 (K48-only) and K48R, in which lysine 48 was substituted with an arginine, to differentiate the ubiquitin linkage types opposed by USP7 on polyubiquitinated ECT2. The results indicated that K48-linked ubiquitin species are the major forms cleaved by USP7 (Figure 5K and S4D). Furthermore, *in vitro* deubiquitination assays with His-Ub conjugated FLAG-ECT2 purified from HeLa cells under high salt and denature condition and USP7/wt or USP7/C223S purified from Sf9 cells revealed that USP7/wt was capable of deubiquitinating ECT2, whereas USP7/C223S was not (Figure 5L and S4E). These results indicated that USP7 targets ECT2 for deubiquitination, indicating that ECT2 is a *bona fide* substrate of USP7. Collectively, these findings together with the observation that ECT2 promotes USP7 intermolecular self-association and self-deubiquitination, support the notion that ECT2 and USP7 form a feedforward circuit in controlling the stabilization of each other.

### MDM2 Is a Key Downstream Effector of ECT2/USP7 Circuit

**Figure 6:**
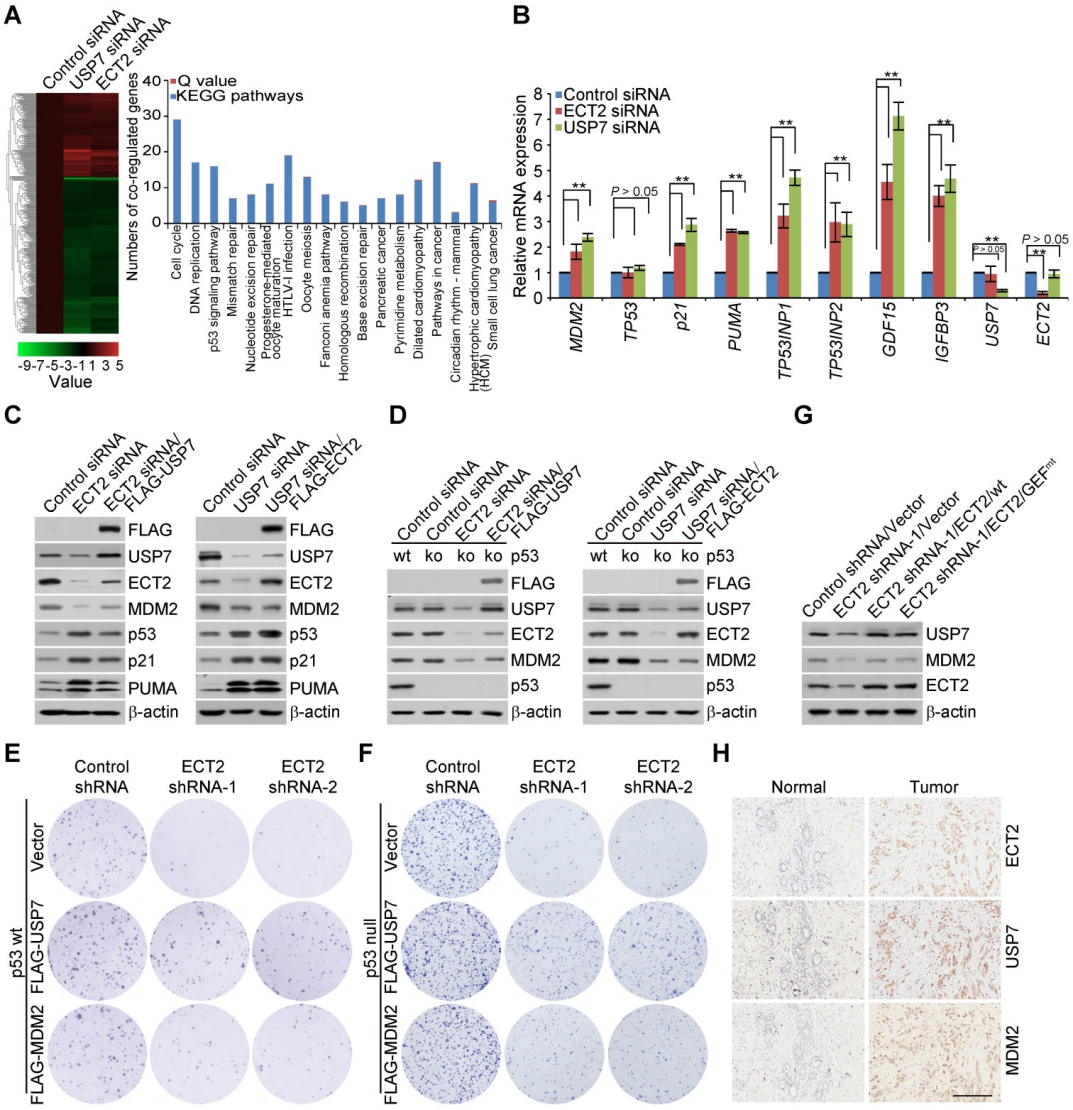
** ECT2/USP7 Circuit Is Implicated in Breast Carcinogenesis through Controlling MDM2 Abundance**. (A) MCF-7 cells were transfected with control siRNA or ECT2 siRNA followed by RNA extraction and deep sequencing. Deep sequencing of RNAs and RNA-seq analysis by Illumina HiSeq^TM^ 2000 with a stringent cut-off (*P* ≤ 10^-5^) identified 747 genes whose expressions were altered upon ECT2 depletion. Cross-analysis of these data with the transcriptomes from USP7-deficient cells by the same sequencing platform (NCBI Sequence Read Archive with accession number SRP066280) were clustered as indicated, and color key and histogram indicating the up- (red) or down-regulation (green) of the targeted genes are shown (left panel). Co-regulated genes were grouped and statistically analyzed according to KEGG pathways with a Q value cut-off of 0.05 and a *P* value cut-off of 0.05 (right panel). (B) MCF-7 cells were transfected with the indicated siRNAs followed by RNA extraction and qRT-PCR analysis of the expression of the indicated genes. Each bar represents the mean ± S.D. for biological triplicate experiments.^ **^P < 0.01*,* one-way ANOVA. (C) FLAG-USP7 was re-introduced into ECT2-deficient MCF-7 cells and cellular extracts were collected followed by Western blotting analysis (left panel). FLAG-ECT2 was re-introduced into USP7-deficient MCF-7 cells and cellular extracts were collected followed by Western blotting analysis (right panel). (D) Experiments analogous to (C) were performed with p53 null MCF-7 cells, in which p53 was knocked out by CRISPR/Cas9 strategy. (E) Colony formation assays with MCF-7 cells stably expressing the indicated shRNAs or genes. Representative images from biological triplicate experiments are shown. (F) Colony formation assays with p53 null MCF-7 cells stably expressing the indicated shRNAs or genes. Representative images from biological triplicate experiments are shown. (G) Western blotting analysis of protein expression with MDA-MB-468 xenograft tumor samples. (H) Immunohistochemistry analysis of the expression levels of ECT2, USP7, and MDM2 in invasive ductal breast tumors and adjacent normal mammary tissues. Representative images from these samples are shown. Scale bar, 200 μm.

Since a majority of USP7 substrates including DNMT1 46, UHRF1 62-64, MDM2 50 and PHF8 42, are involved in chromatin modification or/and gene transcriptional regulation, we hypothesized that disruption of ECT2-USP7 feedforward circuit may transcriptionally alter gene expression programs. Thereby, we employed an unbiased approach, RNA-seq, to characterize the candidate effectors, or effectors associated signaling pathways that could possibly contribute to ECT2-promoted breast carcinogenesis. We identified 365 genes whose expression was altered in both ECT2- and USP7-depleted cells, and these genes were considered as the targets that were co-regulated by ECT2 and USP7 (Figure 6A). Notably, nearly all of these genes were clustered as targets that are transcriptionally regulated in the same direction (up- or down-regulated) by ECT2 and USP7 (Figure 6A), supporting a notion that the function of ECT2 and USP7 is inextricably linked with each other. The genes that were co-regulated by ECT2 and USP7 were then classified into various signaling pathways (Figure 6A). These include cell cycle, DNA replication, and p53 signalling pathway that are critically involved in cell growth and survival (Figure 6A).

Since dysregulation of p53 and its downstream targets, MDM2 in particular, are tightly linked to tumorigenesis including breast cancer, we next chose several representative genes and validated their expressions in MCF-7 cells by qRT-PCR. The results indicated that the mRNA level of *MDM2*, *p21*, *PUMA*, *TP53INP1*, *TP53INP2*, *GDF15*, and *IGFBP3*, is elevated upon knockdown of either ECT2 or USP7, albeit to variable extents, while the expression of *TP53* is unchanged (Figure 6B). These results suggested that p53 signalling pathway is activated in ECT2- or USP7-deficient cells. Furthermore, Western blotting analysis with cell lysates from these cells revealed that the protein abundance of MDM2 is down-regulated, while the expression of p53 and its target genes p21 and PUMA is up-regulated (Figure S5A). The increased expression of MDM2 at mRNA level but decreased in its protein abundance, is likely derived from a negative feedback regulation between MDM2 and p53 at both transcriptional and post-transcriptional level. Our observations are consistent with the understanding that oncogenic protein MDM2, but not tumor suppressor p53, is the most relevant target of USP7 in tumorigenesis 55, 56, 72. Since ECT2 depletion phenocopies the effect of USP7 knockdown, we propose that ECT2/USP7 circuit is potentially involved in controlling the MDM2/p53 signalling pathway.

As p53 is the most frequently mutated gene in human cancer and one common type of these mutations affecting p53 are loss of function 73, we wondered whether p53 is required for ECT2/USP7 circuit-regulated downstream effects. To test this idea, we first created a p53 null MCF-7 cell with CRISPR/Cas9 system. Then, USP7 was re-introduced into ECT2-deficient cells with intact or null p53 and the results indicated that ECT2 depletion associated MDM2 destabilization could be reverted by forced expression of USP7 in each type of these cells (Figure 6C and 6D). Interestingly, ECT2 deficiency associated USP7 reduction is largely overcome by forced expression of USP7 (Figure 6C and 6D), possibly due to the fact that the exogenously expressed USP7 remains, albeit not efficiently, undergoing self-deubiquitination thus stabilization. This deduction is supported by the finding that purified USP7, without ECT2 addition, is still able to remove ubiquitin chains from USP7/C223S as demonstrated in the *in vitro* deubiquitination assays (Figure 4H). However, overexpression of ECT2 in USP7 depleted cells, in which the protein level of overexpressed ECT2 was higher than the endogenous one in control cells, failed to rescue USP7 deficiency associated effects (Figure 6C and 6D). In cells expressing USP7 siRNA, although endogenous ECT2 is subject to polyubiquitination thus proteasome degradation, a large amount of transfected ECT2 molecules may succeed in escaping the control of its potential E3 ligases thus degradation. Collectively, these results indicated that the involvement of ECT2 in MDM2 stabilization is dependent on USP7, and support the notion that ECT2/USP7 circuit plays a critical role in promoting the stabilization of MDM2 in both p53-proficient and p53-deficient cells.

To further investigate the biological significance of this circuit, colony formation assay was performed. The results showed that ECT2 deletion associated growth retardation of breast cancer cells could be reverted, to certain extent, by forced expression of MDM2 or USP7/wt (Figure 6E and 6F; Figure S5B and S5C), but not USP7/C223S (Figure S5D and S5E). The effect is conserved from the parental MCF-7 cells (Figure 6E and S5D) to its isogenic p53 knockout cells (Figure 6F and S5E). Then, we examined the expression of MDM2 and USP7 in MDA-MB-468 xenograft tumor samples. The results indicated that ECT2 depletion are associated with downregulation of MDM2 and USP7, and this effect could be largely reverted in tumors expressing either wild type ECT2 or GEF activity-deficient ECT2 (Figure 6G). These observations not only underline the importance of ECT2 and USP7 formed feedforward loop in the survival of breast cancer cells, but also favor the argument that ECT2, with the help of USP7 and MDM2, promotes breast cancer cell survival regardless of the presence or absence of p53.

To extend our observations to a clinicopathologically-relevant context, we then analyzed the protein expression levels of ECT2, USP7, and MDM2 with breast carcinoma samples and histologically normal mammary tissues. We found that, when staining was scored according to the mean intensity extent of immunopositivity, ECT2 and USP7 together with MDM2 were highly expressed in breast carcinoma samples (Figure 6H and S5F), and the levels of their expression correlated with each other (Figure S5G). Collectively, these results suggested that ECT2/USP7 circuit is potentially linked to breast carcinogenesis, and ECT2 coordinates with USP7 to promote breast cancer cell survival, at least, via activating oncogenic MDM2 (Figure S5H).

## Discussion

In this study, we revealed a GEF activity-independent role of ECT2 in promoting breast carcinogenesis. Specifically, we uncovered that ECT2 and USP7 form a positive feedback loop, in which ECT2 promotes USP7 intermolecular self-association, -deubiquitination and -stabilization, and reciprocally, USP7 deubiquitinates and stabilizes ECT2. This circuit eventually promotes breast cancer cell survival, at least, through sustaining the expression of oncogenic protein MDM2.

To date, nearly all studies are focusing on investigating the molecular or biological functions of the GEF activity of ECT2, but whether ECT2 has a GEF-independent role is rarely studied. Consistent with the argument that the role of ECT2 in transformation and cytokinesis is separated and distinct 12, 13, it is reported that nuclear ECT2 contributes to transformed growth but not cytokinesis in tumor cells 15, 54. In particular, ECT2 acts as a nuclear GEF for RAC1 and drives tumor initiation of non-small-cell lung carcinoma through regulating rRNA synthesis 15. Here, we uncovered a GEF activity-independent role of nuclear ECT2 in facilitating anchorage-independent growth and survival of breast cancer cells, indicating that the GEF activity is not a prerequisite for ECT2-promoted tumorigenesis. In support of this, we found that ECT2 and USP7 form a distinct complex in the absence of nuclear Rho-GTPase CDC42 and RAC1, and depletion either or both of these Rho-GTPases had minor effect on the expression of USP7. Interestingly, fluorescent microscopy and BRET approach demonstrated that ECT2 acts as a scaffolding protein to facilitate USP7 oligomerization. In this manner, ECT2 controls the abundance of USP7 via promoting USP7 intermolecular self-deubiquitination. Also, we showed that the GEF activity-deficient ECT2 (ECT2/GEF^mt^) behaves similarly as wild type ECT2 in controlling protein-protein interaction and the molecular behaviors of USP7 towards itself. Finally, we revealed that ECT2-deficiency induced growth retardation could be reverted, to certain extent, by forced expression of USP7 with colony formation assays or GEF activity-deficient ECT2 via xenograft experiments. These evidences support the notion that USP7 is likely an essential downstream effector of GEF activity-deficient ECT2 in breast carcinogenesis.

USP7 is one of the most extensively studied deubiquitinating enzymes, and it requires careful regulation of the catalytic activity to function properly. The first layer of regulation is intrinsic in USP7's multi-domain architectures. For instance, full activity of USP7 demands the C-terminal UBL domain folding back onto the catalytic domain, to allow the switch of the active site to a catalytically competent state by the very C-terminal peptide 45, 59. A second layer of regulation is the ability of USP7 to form complex with other proteins that allosterically modulate its catalytic activity 44, 45. Here, we failed to identify ECT2 as a modulator in controlling the enzymatic activity of USP7 as revealed by *in vitro* deubiquitinaiton assays with pure ubiquitin linkages, albeit the UBL domain is required for the association of USP7 with ECT2. Surprisingly, we uncovered that ECT2 promotes intermolecular self-association and self-deubiquitination of USP7. Since ECT2 is also reported to oligomerize *in vivo* 26, it is likely that the association of ECT2 with USP7 requires oligomeric formation of each protein. Although the molecular details regarding how the assemble of the ECT2/USP7 protein complex is spatially and temporally regulated remain to be determined, our study provides a new molecular insight in controlling the action of USP7. Also, we found that ECT2 is a *bona fide* substrate of USP7, indicating that these two proteins reciprocally regulate each other. If this is the case, the importance of this positive feedback regulation, cannot be overlooked. Similar to the phenotype associated with ECT2 knockout, mice homozygous for a null allele of USP7 also show embryonic growth arrest and die between E6.5 and E7.5 74. This observation further implies a functional link between ECT2 and USP7, despite it is still unknown whether the developmental defects associated with genetic knockout of ECT2 or USP7 could be attributed to the impairment or disruption of the feedforward circuit shaped by ECT2 and USP7.

Taking into account that ECT2 is regulated during the cell cycle by APC^Cdh1^ 71 and USP7 has a critical role in S phase progression 75, 76, USP7 downregulation or prolonged inhibition may lead to alterations of cell cycle re-distribution thus an indirect effect on the ubiquitination and degradation of ECT2. Although we could not exclude this possibility, from the following observations we believe that ECT2 is directly targeted by USP7 for deubiquitination and stabilization: 1) ECT2 is physically associated with USP7 *in vitro* and *in vivo*, as revealed by pull down assays, immunoprecipitation analysis and fluorescent stainings followed by confocal microscopy; 2) recombinant USP7 from insect cells is able to de-ubiquitinate ECT2 *in vitro*; 3) recombinant ECT2 from mammalian cells promotes USP7 self-deubiquitination* in vitro*, suggesting ECT2 and USP7 are physically connected and functionally linked; and 4) when cells were treated with USP7 inhibitors HBX41,108 and GNE-6640 in a shorter time window (4 h), the protein abundance of ECT2 is decreased and its ubiquitination level is upregulated. Additionally, it has been reported that both ECT2 and USP7 are required for G_1_-S transition of the cell cycle 77, further pointing that these two molecules may act in coordination. Interestingly, we found E3 ligase thyroid hormone receptor interactor 12 (TRIP12) could be also pulled down by ECT2 with immunopurification followed by mass spectrometry analysis. TRIP12 has been reported to interact with and act as an E3 ubiquitin ligase for USP7 78, 79. Reciprocally, USP7 functions to stabilize TRIP12 by its deubiquitination activity 80. Dysregulated TRIP12 has been implicated in several types of cancer, such as breast, pancreatic, and liver cancer 80, 81. In the future work, we will try to investigate whether TRIP12 could ubiquitinate and destabilize ECT2 and impact on the feed-forward circuit shaped by ECT2 and USP7 in tumorigenesis.

Targeting ECT2, USP7 or their interaction molecular interface may, thus, abrogate the positive feedback circuit forming by these two molecules, and provide an effective treatment of breast cancer patients. Indeed, specific small-molecule antagonists toward the catalytic activity of USP7 72, 82 or attenuating ubiquitin binding to USP7 55, 56 with high affinity and specificity have been developed recently, and we revealed that GNE-6640 56, which preferentially disrupts the binding of USP7 to K48-linked ubiquitin conjugates, destabilizes ECT2 and impairs USP7 self-stabilization. Since USP7 could be only co-immunoprecipitated with the full length ECT2 and the catalytic domain and UBL domain as a whole is required for the molecular interface connection between USP7 and ECT2, we propose that the interaction of ECT2 with USP7 may depend on the formation of higher structure of these two molecules. Thus, it seems impossible to inhibit the USP7-ECT2 interaction by using a dominant negative mutant or small molecular inhibitors. Next, we found that both ECT2 and USP7 are significantly overexpressed in a subset of breast cancer patients and the levels of these two factors positively correlate with each other. In a way, our study provides a rationale for validating USP7/ECT2 as a viable therapeutic target for breast cancer. Although highly expressed USP7 has been linked to breast carcinogenesis 42, it is less defined what determines the upregulation of USP7 in breast cancer. Here, we propose that transcriptionally upregulated ECT2 may enhance USP7 self-deubiquitination thus stabilization in breast cancer. The *ECT2* gene resides on chromosome 3q26, a region frequently amplified in lung squamous cell carcinomas (LSCC) 13, esophageal squamous cell carcinomas (ESCCs) 83, 84, and ovarian tumors 85. Many tumors, including lung adenocarcinomas (LACs), do not harbor amplification, but exhibit elevated expression of ECT2 13. These results suggest that mechanisms other than amplification are involved in controlling ECT2 expression in human tumors. Interestingly, it is reported that *ECT2* is transcriptionally repressed by p53 77. Considering that MDM2/p53 signaling axis is one of the key pathways regulated by ECT2/USP7 circuit as shown in our study, we envisioned that in tumors with null or lower expressed p53, this circuit is helpful in maintenance of highly expressed ECT2 and USP7, and will be supportive in activating MDM2-dependent oncogenic pathway.

Here, we found that ECT2 coordinates with USP7 to form a feedforward circuit and promote breast cancer cell survival in a GEF activity-independent manner. These observations not only broad the biological function of ECT2, but also provide a new mechanistic insight for ECT2 in promoting tumorigenesis. Due to the complex biological heterogeneity of breast cancer, it will be important to determine to what extent the ECT2/USP7 circuit affects breast cancer cell survival across genetically distinct subgroups of breast cancer. Since the expression of ECT2 in different subgroups with distinct histological or molecular traits is all elevated, we hypothesized that ECT2 might play a conserved role in survival promotion of all subtypes of breast carcinoma even though its GEF activity is absent or insufficient. Also, it remains to be tested whether the positive feedback regulation of ECT2 and USP7 is applicable for other types of cancer. Nevertheless, our study identifies ECT2 as an essential regulatory scaffolding protein in controlling the function of USP7, and indicates that ECT2/USP7 circuit is critically implicated in breast carcinogenesis.

## Supplementary Material

Supplementary figures and tables.
