# Supplementary material for: A feedforward circuit shaped by ECT2 and USP7 contributes to breast carcinogenesis
Source: Theranostics. 2020 Aug 29;10(23):10769–90. doi: 10.7150/thno.46878 (PMC7482815; doi:10.7150/thno.46878)
Supplement: Supplementary file 1 — Supplementary figures and tables. [file thnov10p10769s1.pdf]

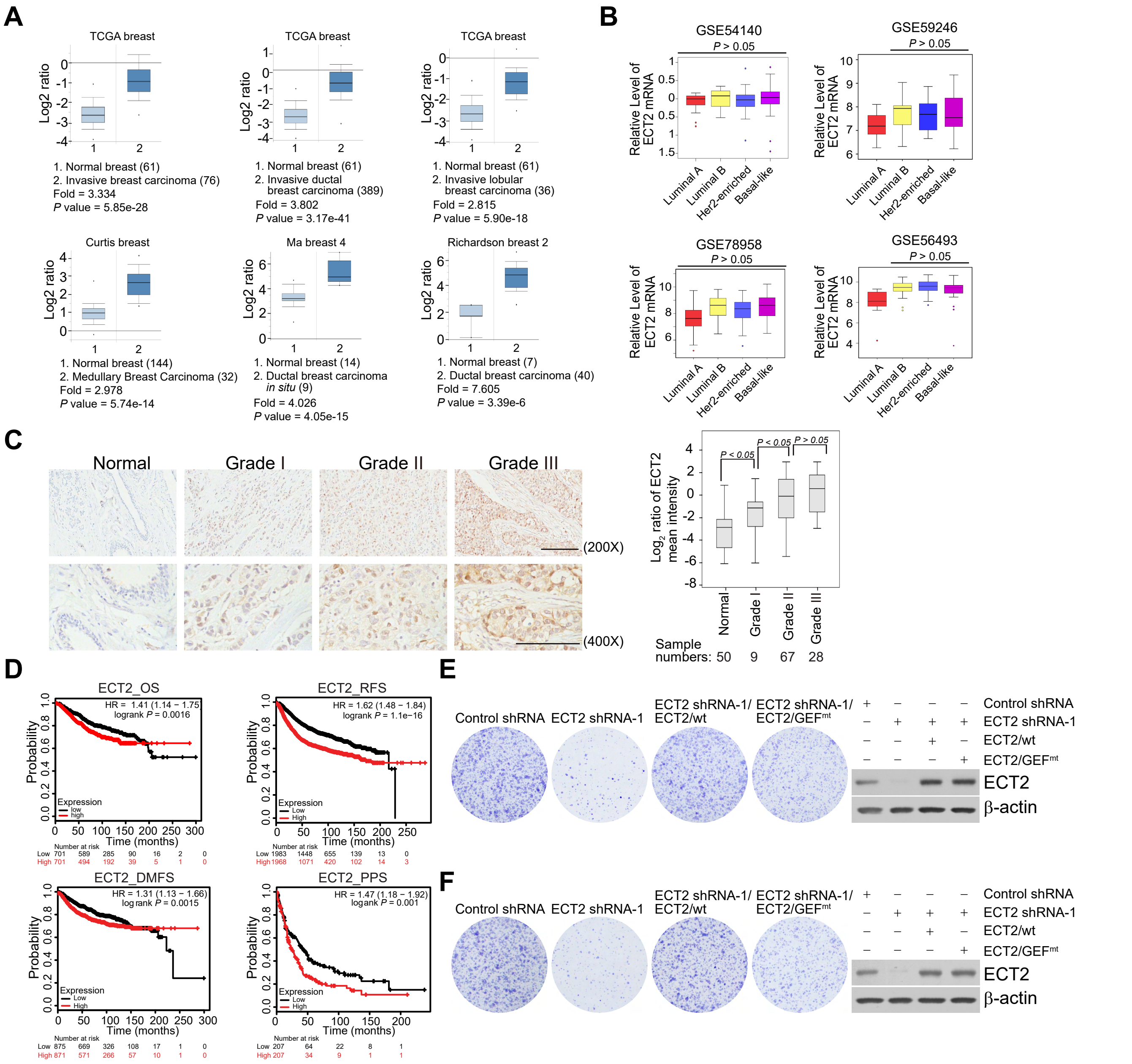

Figure S1 (Figure 1 continued). ECT2 Deregulation Contributes to Breast Carcinogenesis. (A) Box plots of ECT2 transcript levels in normal human mammary tissues and distinct histological breast carcinoma samples based on six independent analyses from Oncomine. (B) Box plots of ECT2 transcript levels in breast carcinoma samples with distinct molecular traits based on four independent datasets from GEO. *P* value was determined by one-way analysis of variance (ANOVA). (C) Immunohistochemistry analysis of the expression levels of ECT2 in different histologic types of invasive ductal breast tumors and adjacent normal mammary tissues. Representative images (200 × and 400 × magnification as indicated) from these samples are shown (left panel). Scale bar, 200 µm. Scores of the stained sections were determined by evaluating the nuclear intensity of immunopositivity by Image-pro Plus software and are presented with box plots (right panel). *P* values were determined by one-way ANOVA. (D) Kaplan-Meier survival analysis for the relationship between survival time of breast cancer patients and the mRNA expression level of ECT2 with survival packages from K-M plotter database. Sample size is shown as indicated. OS, overall survival; RFS, relapse free survival; DMFS, distant metastasis free survival; and PPS, post progression survival. (E) Colony formation assays were conducted with ZR-75-1 cells stably expressing the indicated shRNAs or genes. Representative images from biological triplicate experiments are shown. The expression of ECT2 was examined by Western blotting. (F) Colony formation assays were conducted with MDA-MB-468 cells stably expressing the indicated shRNAs or genes. Representative imag es from biological triplicate experiments are shown. The expression of ECT2 was examined by Western blotting.

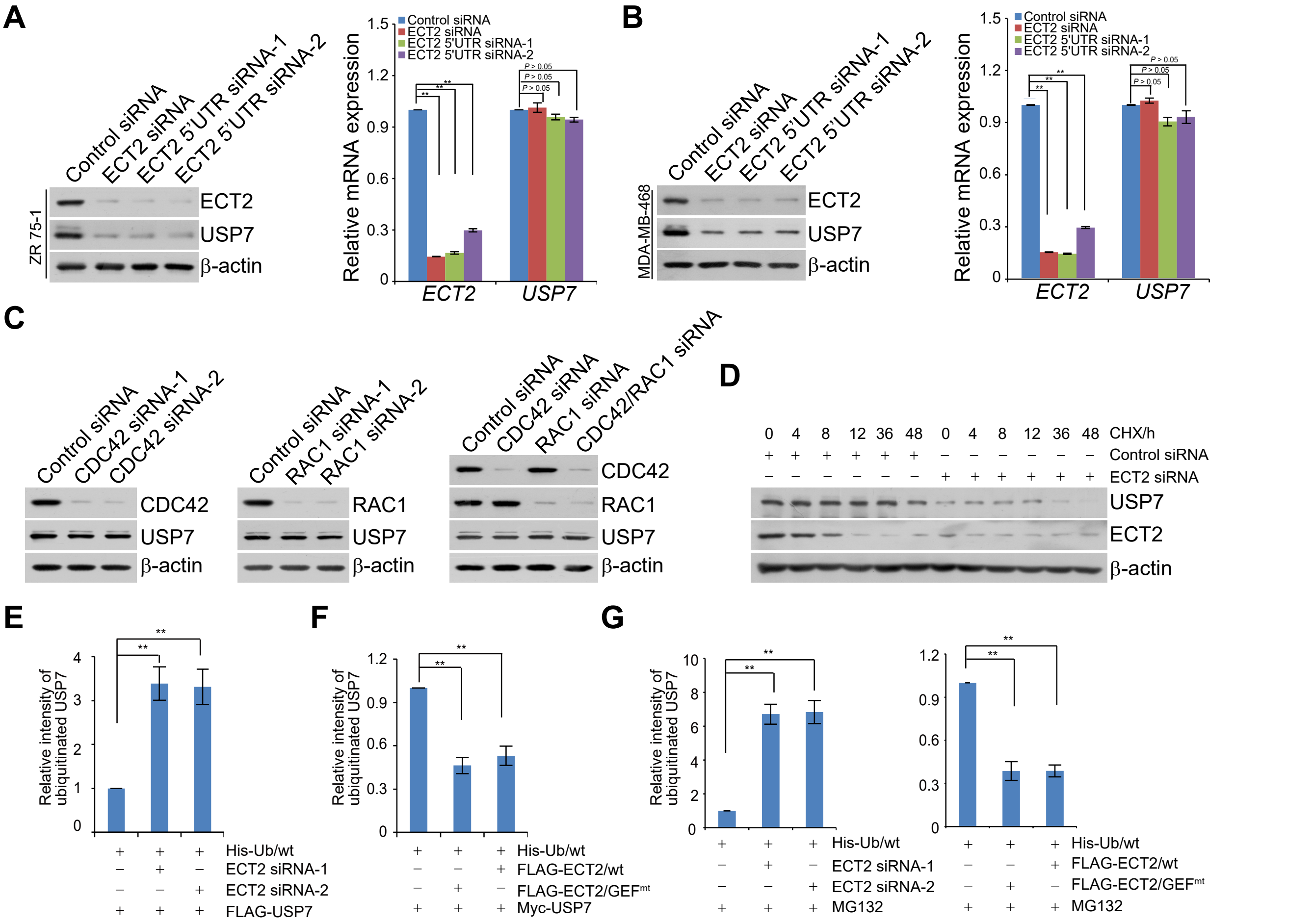

Figure S2 (Figure 3 continued). ECT2 Prevents USP7 Degradation through Opposing Its Polyubiquitination. (A) ZR-75-1 cells were transfected with control siRNA or different sets of ECT2 siRNAs. Cellular extracts and total RNA were prepared and analyzed by Western blotting and qRT-PCR, respectively. Each bar represents the mean ± S.D. for biological triplicate experiments. $^{**}P < 0.01$, one-way ANOVA (B) Experiments analogous to (A) were performed with MDA-MB-468 cells. Each bar represents the mean ± S.D. for biological triplicate experiments. $^{**}P < 0.01$, one-way ANOVA. (C) MCF-7 cells were transfected with control siRNA, CDC42 siRNA, RAC1 siRNA, or CDC42 and RAC1 siRNA in combination. Cellular extracts were collected and analyzed by Western blotting with antibodies against the indicated proteins. (D) MCF-7 cells transfected with control siRNA or ECT2 siRNA were treated with 50 μg/mL cycloheximide (CHX) and harvested at the indicated time followed by Western blotting analysis. (E) Quantitation and statistical analysis of ubiquitinated USP7 from experiments in Figure 3F. Each bar represents the mean ± S.D. for biological triplicate experiments. $^{**}P < 0.01$, one-way ANOVA. (F) Quantitation and statistical analysis of ubiquitinated USP7 from experiments in Figure 3G. Each bar represents the mean ± S.D. for biological triplicate experiments. $^{**}P < 0.01$, one-way ANOVA. (G) Quantitation and statistical analysis of ubiquitinated USP7 from experiments in Figure 3H. Each bar represents the mean ± S.D. for biological triplicate experiments. $^{**}P < 0.01$, one-way ANOVA.

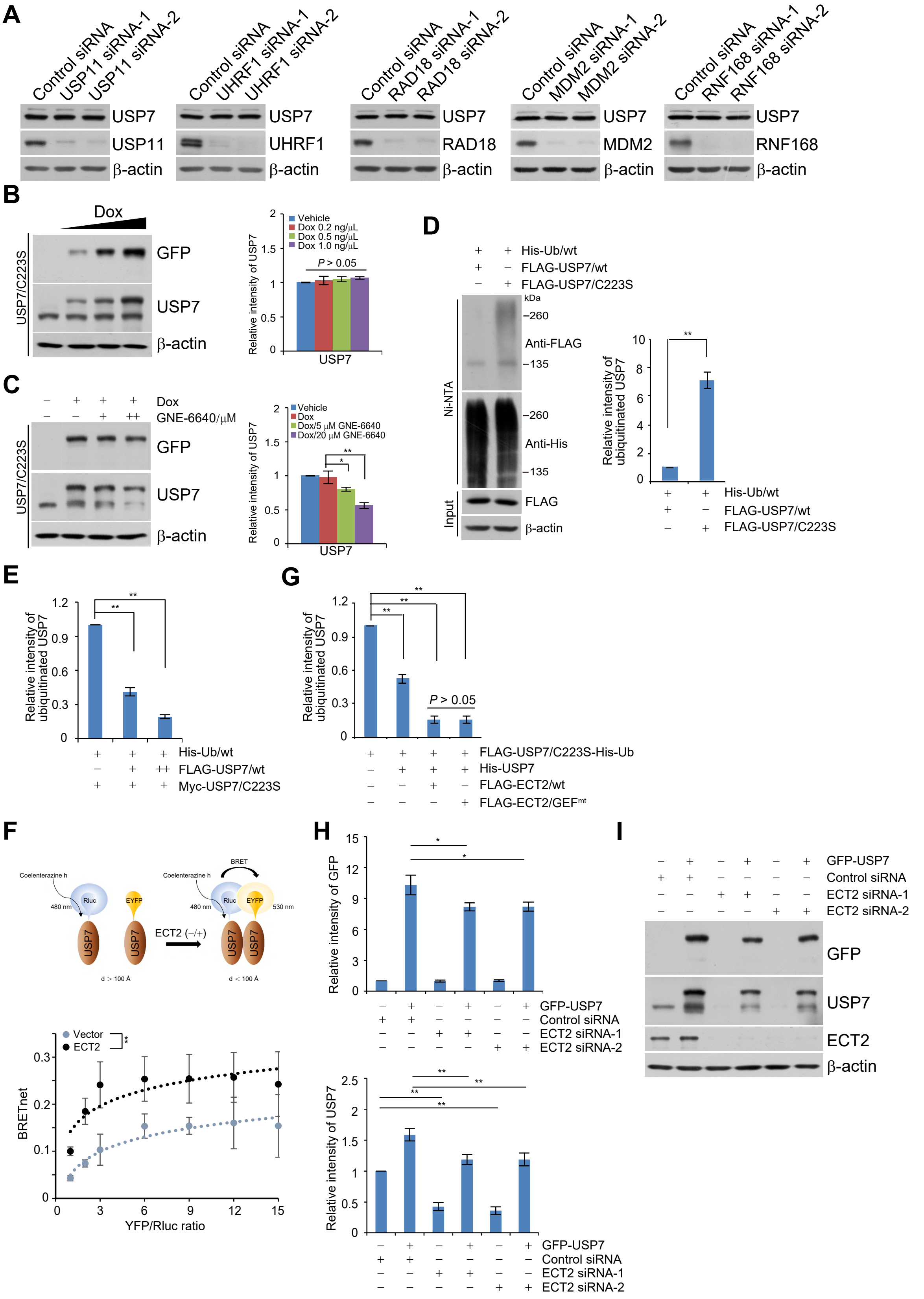

Figure S3 (Figure 4 continued). ECT2 Facilitates USP7 Intermolecular Self-association, -Deubiquitination and -Stabilization. (A) MCF-7 cells were transfected with control siRNA or siRNAs targeting USP11, UHRF1, RAD18, MDM2 or RNF168. Cellular extracts were prepared and analyzed by Western blotting. (B) MCF-7 cells allowing Dox-inducible expression of stably integrated GFP-USP7/C223S were cultured in the presence of increasing amounts of doxycycline. Cellular extracts were prepared and analyzed by Western blotting. For USP7 bands, the higher one with larger molecular weight represents GFP-tagged USP7/C223S, while the lower one indicates endogenous USP7. The quantitation of endogenous USP7 was shown. Each bar represents the mean ± S.D. for biological triplicate experiments. *P* value was determined by one-way ANOVA. (C) MCF-7 cells allowing Dox-inducible expression of stably integrated GFP-USP7/C223S were cultured in the absence or presence of USP7 inhibitor GNE-6640 for 24 h. Cellular extracts were prepared and analyzed by Western blotting. For USP7 bands, the higher one with larger molecular weight represents GFP-tagged USP7/C223S, while the lower one indicates endogenous USP7. The quantitation of endogenous USP7 was shown. Each bar represents the mean ± S.D. for biological triplicate experiments. $*P < 0.05$，$**P < 0.01$, one-way ANOVA. (D) Cellular extracts from HeLa cells expressing USP7/wt or USP7/C223S and His-Ub/wt were prepared for affinity-based precipitation assays via Ni-NTA agarose beads. The quantitation of ubiquitinated USP7 was shown. Each bar represents the mean ± S.D. for biological triplicate experiments. $**P < 0.01$, one-way ANOVA. (E) The quantitation of ubiquitinated USP7 in experiments from Figure 4E was shown. Each bar represents the mean ± S.D. for biological triplicate experiments. $**P < 0.01$, one-way ANOVA. (F) Transfer energy between EYFP-USP7 and Rluc-USP7 in the absence or presence of ECT2 was examined by BRET assay. Control cells or HEK293T cells stably expressing FLAG-ECT2 were co-transfected with a constant amount of Rluc-USP7 plasmid and increasing amounts of EYFP-USP7. Then, fresh coelenterazine h (5 μM) was added to cells and Bioluminescence emission profiles were measured. Each bar represents the mean ± S.D. for biological triplicate experiments. $**P < 0.01$，two-way ANOVA. (G) The quantitation of ubiquitinated USP7 in experiments from Figure 4H was shown. Each bar represents the mean ± S.D. for biological triplicate experiments. $**P < 0.01$, one-way ANOVA. (H) The quantitation of GFP and endogenous USP7 in experiments from Figure 4I was shown. Each bar represents the mean ± S.D. for biological triplicate experiments. *P* value was determined by one-way ANOVA. $*P < 0.05$, $**P < 0.01$. (I) Experiments analogous to Figure 4I were performed with USP7 antibody from Sigma (05-1946).

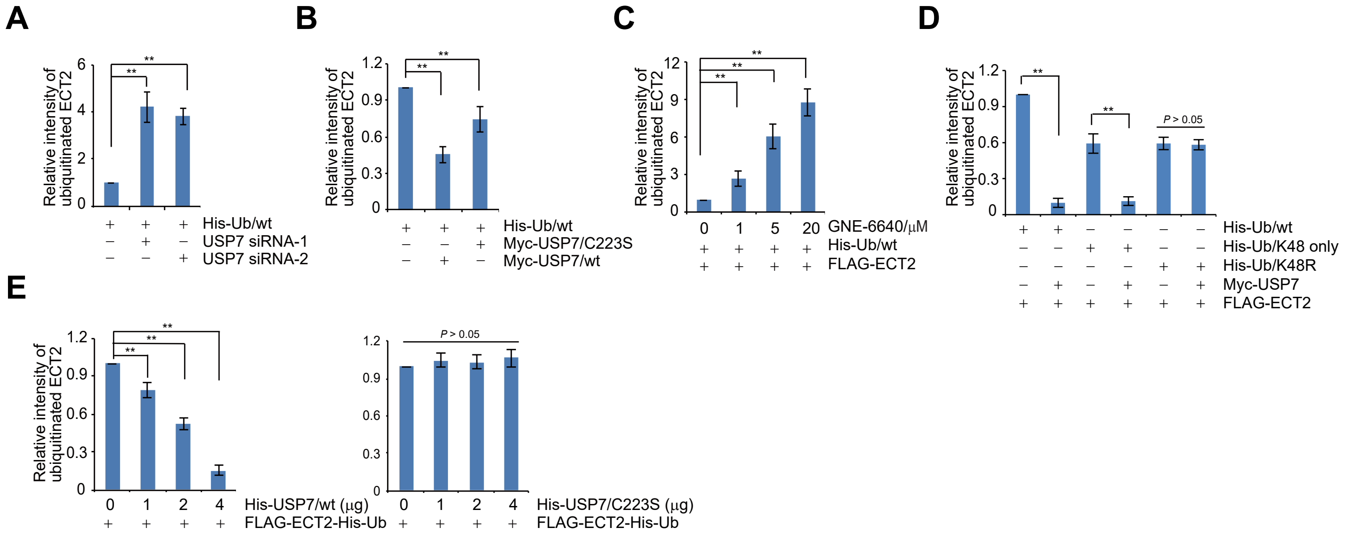

Figure S4 (Figure 5 continued). USP7 Promotes ECT2 Stabilization and Deubiquitination. (A) The quantitation of ubiquitinated ECT2 in experiments from Figure 5H was shown. Each bar represents the mean ± S.D. for biological triplicate experiments. **$P < 0.01$, one-way ANOVA. (B) The quantitation of ubiquitinated ECT2 in experiments from Figure 5I was shown. Each bar represents the mean ± S.D. for biological triplicate experiments. **$P < 0.01$, one-way ANOVA. (C) The quantitation of ubiquitinated ECT2 in experiments from Figure 5J was shown. Each bar represents the mean ± S.D. for biological triplicate experiments. **$P < 0.01$, one-way ANOVA. (D) The quantitation of ubiquitinated ECT2 in experiments from Figure 5K was shown. Each bar represents the mean ± S.D. for biological triplicate experiments. **$P < 0.01$, one-way ANOVA. (E) The quantitation of ubiquitinated ECT2 in experiments from Figure 5L was shown. Each bar represents the mean ± S.D. for biological triplicate experiments. **$P < 0.01$, one-way ANOVA.

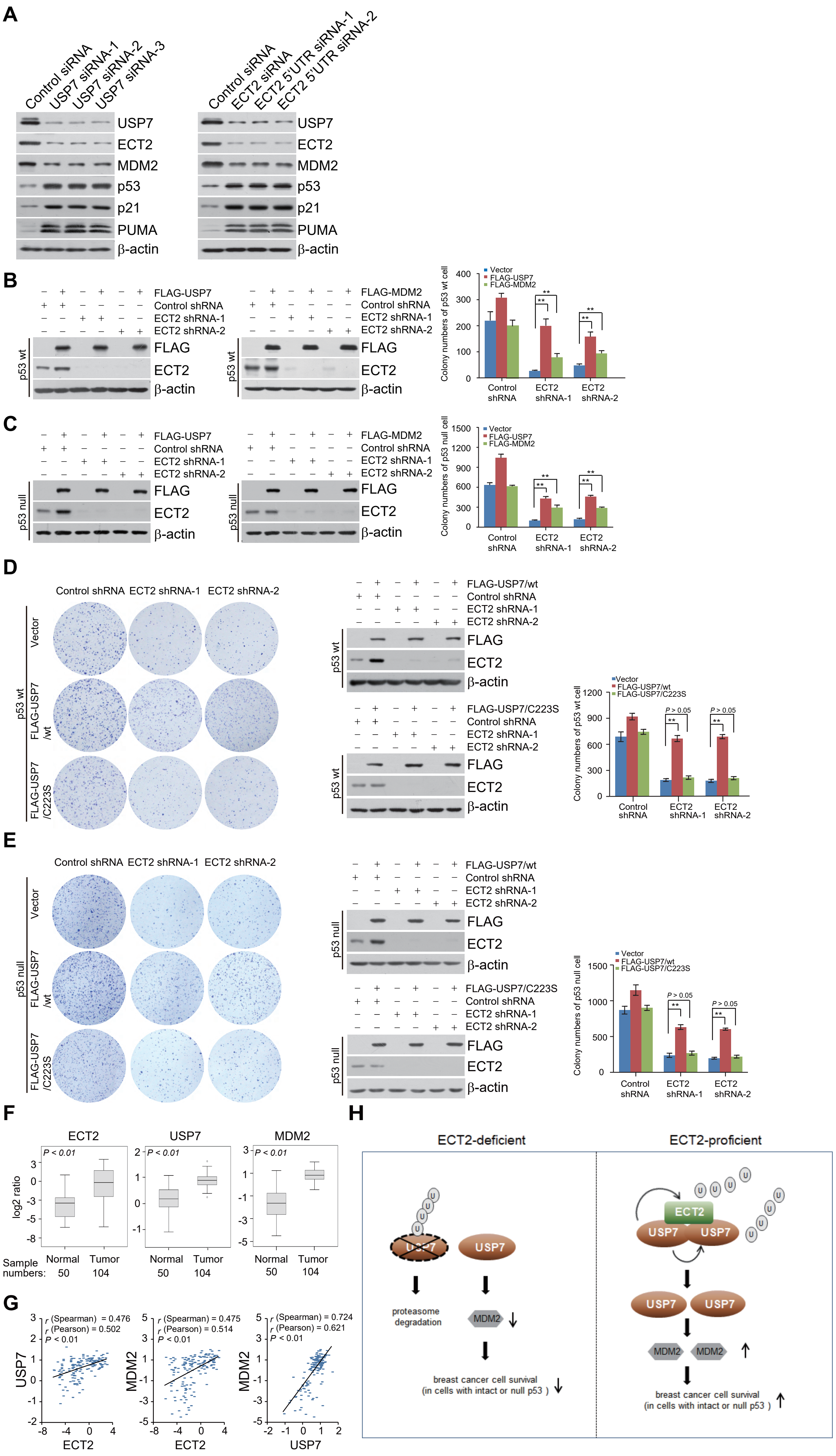
A
Control siRNA
USP7 siRNA-1
USP7 siRNA-2
USP7 siRNA-3
ECT2 siRNA
ECT2 5'UTR siRNA-1
ECT2 5'UTR siRNA-2
USP7
ECT2
MDM2
p53
p21
PUMA
β-actin
B
FLAG-USP7
FLAG-MDM2
Control shRNA
ECT2 shRNA-1
ECT2 shRNA-2
p53 wt
FLAG
ECT2
β-actin
Vector
Colony numbers of p53 wt cell
C
p53 null
Colony numbers of p53 null cell
D
FLAG-USP7/wt
FLAG-USP7/C223S
P > 0.05
E
F
log2 ratio
Normal
Tumor
Sample numbers:
50
104
P < 0.01
G
r (Spearman) = 0.476
r (Pearson) = 0.502
r (Spearman) = 0.475
r (Pearson) = 0.514
r (Spearman) = 0.724
r (Pearson) = 0.621
H
ECT2-deficient
ECT2-proficient
proteasome degradation
breast cancer cell survival
(in cells with intact or null p53)

Figure S5 (Figure 6 continued). ECT2/USP7 Circuit Is Implicated in Breast Carcinogenesis through Controlling MDM2. (A) Cellular extracts were collected from MCF-7 cells expressing the indicated siRNAs and examined by Western blotting. (B) Cellular extracts were collected from MCF-7 cells stably expressing the indicated shRNAs or/and genes and examined by Western blotting. Colony numbers from Figure 6E were counted and statistically analysed. $^{**}P < 0.01$, one-way ANOVA. (C) Cellular extracts were collected from MCF-7 cells (p53 null) stably expressing the indicated shRNAs or/and genes and examined by Western blotting. Colony numbers from Figure 6F were counted and statistically analysed. $^{**}P < 0.01$, one-way ANOVA. (D) Colony formation assays with MCF-7 cells stably expressing the indicated shRNAs or/and genes. Representative images from biological triplicate experiments are shown. Cellular extracts were collected and examined by Western blotting. Colony numbers were counted and statistically analyzed. $^{**}P < 0.01$, one-way ANOVA. (E) Colony formation assays with p53 null MCF-7 cells stably expressing the indicated shRNAs or/and genes. Representative images from biological triplicate experiments are shown. Cellular extracts were collected and examined by Western blotting. Colony numbers were counted and statistically analyzed. $^{**}P < 0.01$, one-way ANOVA. (F) Scores of the stained sections from Figure 6H were determined by evaluating the nuclear intensity of immunopositivity by Image-pro Plus software and are presented with box plots. *P* values were determined by two-tailed unpaired Student's t-test. (G) The correlation plot, coefficient and *P* values were analyzed as indicated. (H) Working model. ECT2 coordinates with USP7, in a GEF activity-independent manner, to form a feedforward circuit and promote breast cancer cell survival. In ECT2-deficient cells, monomeric USP7 is susceptible to be ubiquitinated and degraded by proteasome, accompanying by low abundance of MDM2, while in ECT2-proficient cells, ECT2 acts as a scaffolding protein to facilitate USP7 intermolecular self-association, -deubiquitination, and -stabilization, and, in turn, USP7 deubiquitinates and stabilizes ECT2 as well as MDM2. In this manner, ECT2 promotes breast cancer cell survival regardless of the expression status of p53.

Figure S6. Uncropped Blots Related to Figures 1-6 and Supplemental Figures 1-5.

Full unedited gel for Figure 1A

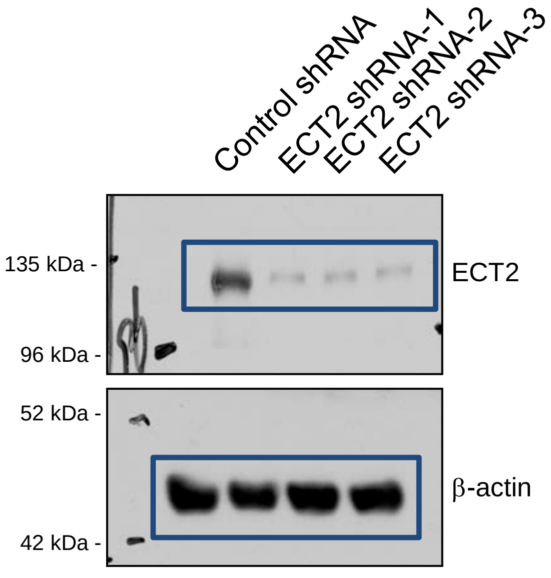

Full unedited gel for Figure 1B

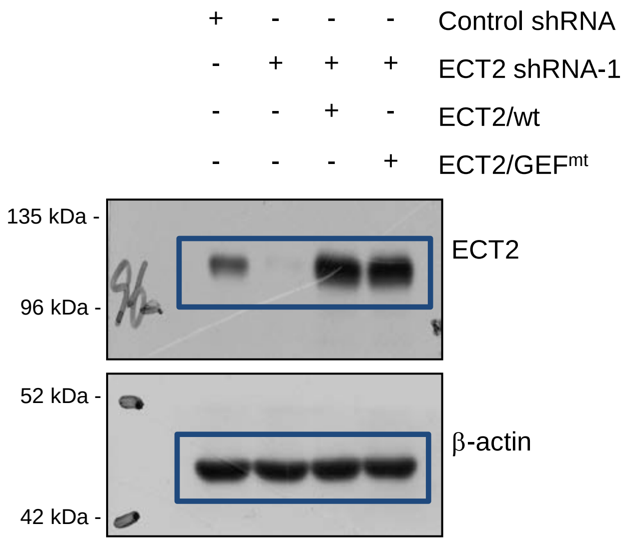

Full unedited gel for Figure 1C

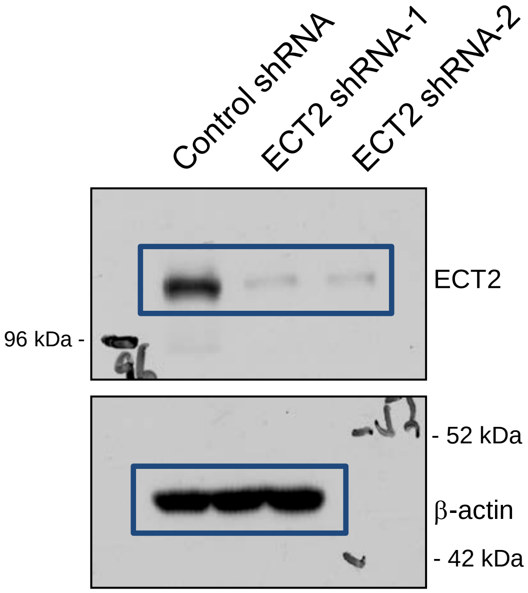

Full unedited gel for Figure 1D

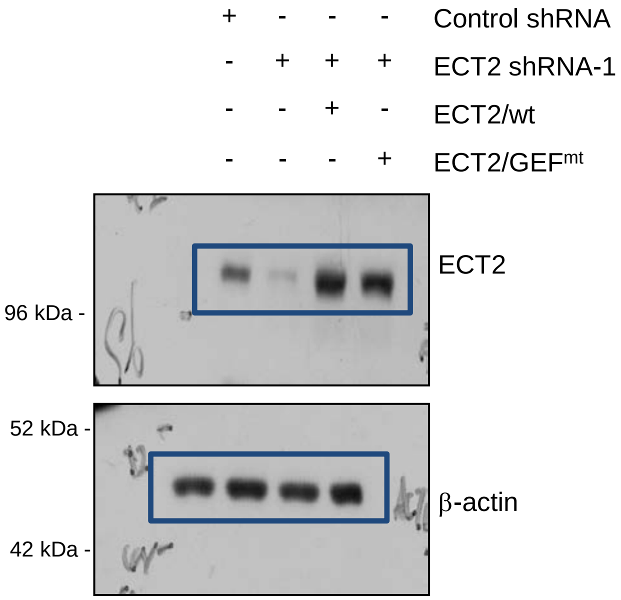

Full unedited gel for Figure 1E

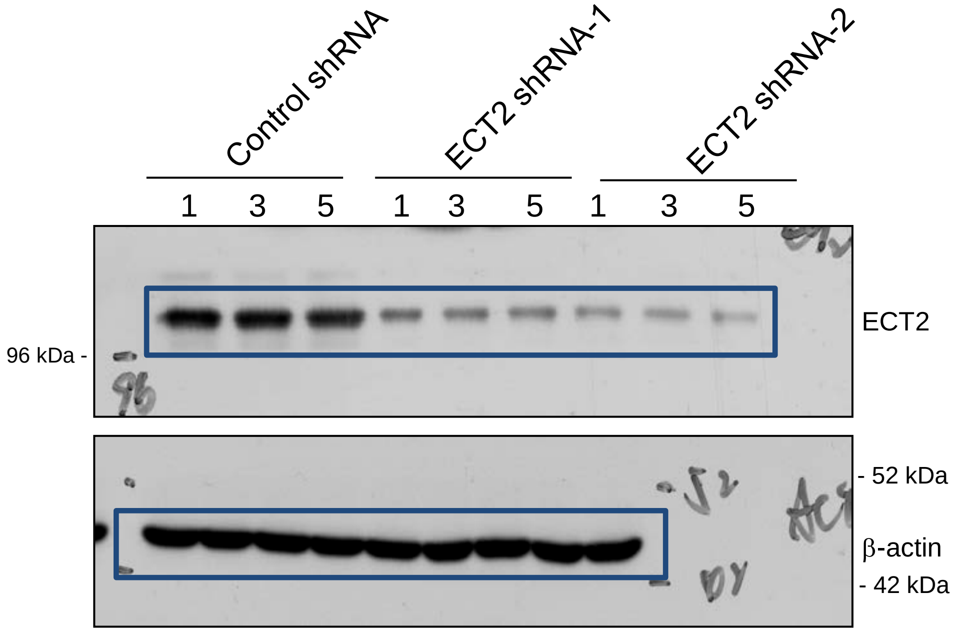

Full unedited gel for Figure 1F

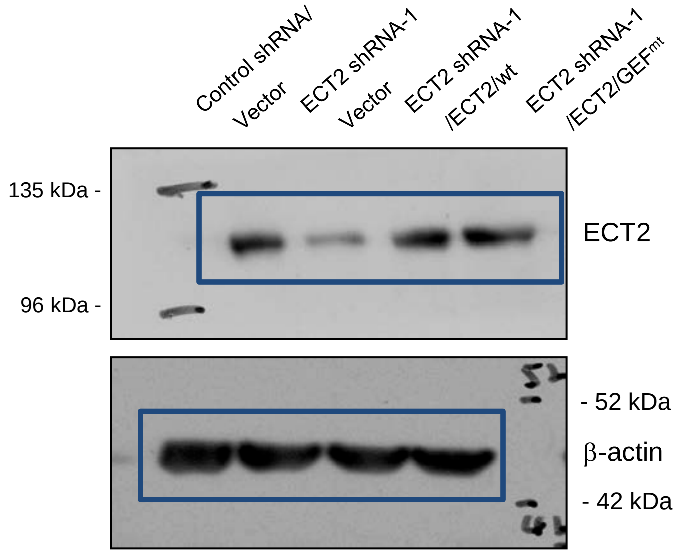

Full unedited gel for Figure 2A

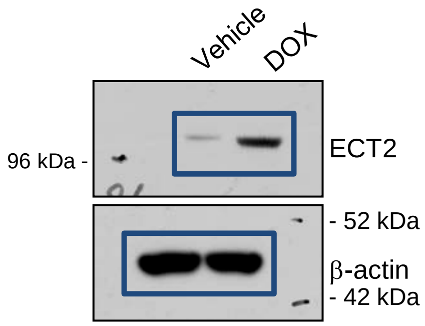

Full unedited gel for Figure 2B

MCF-7

Left panel

Right panel

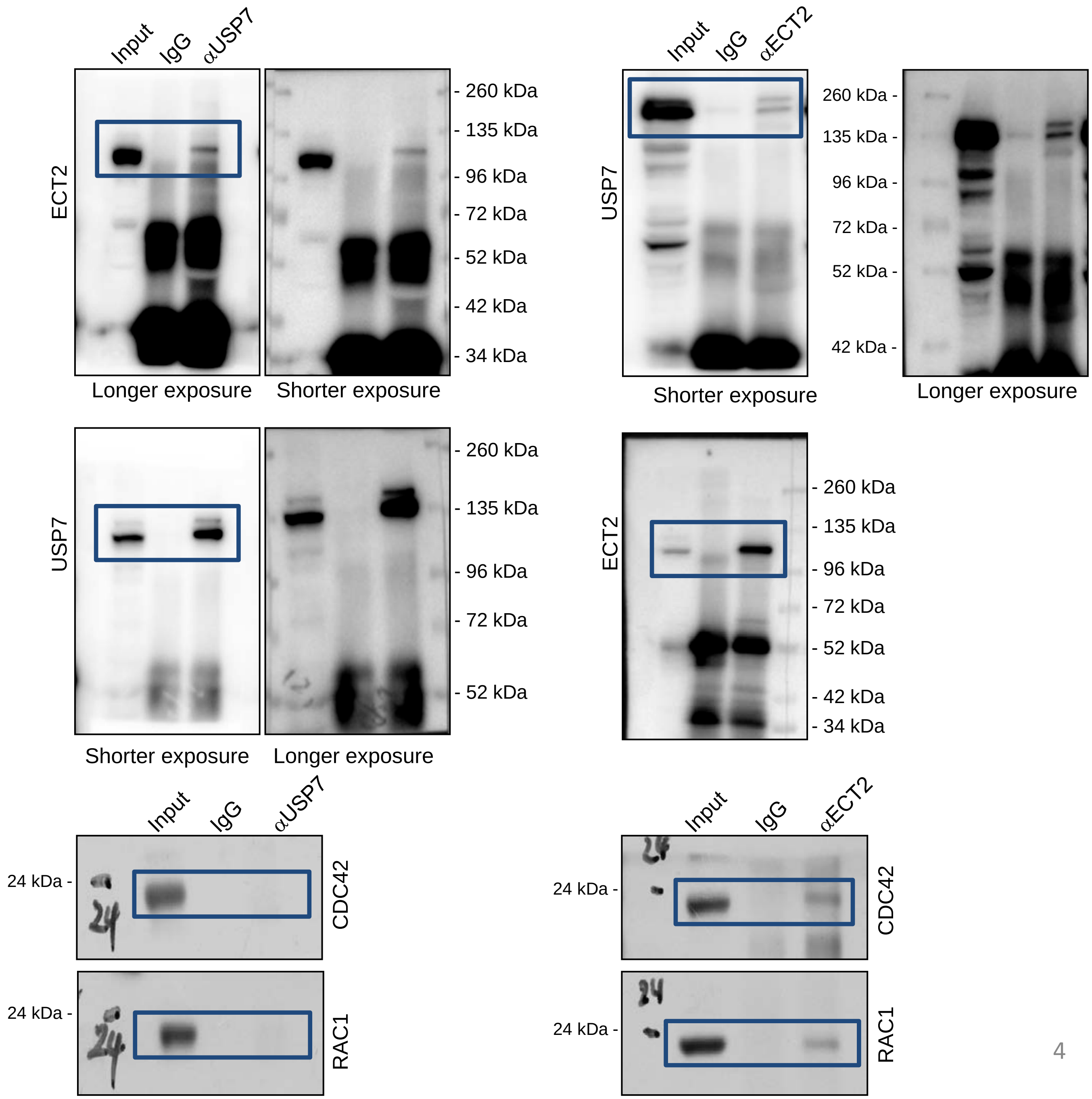

HeLa

Left panel

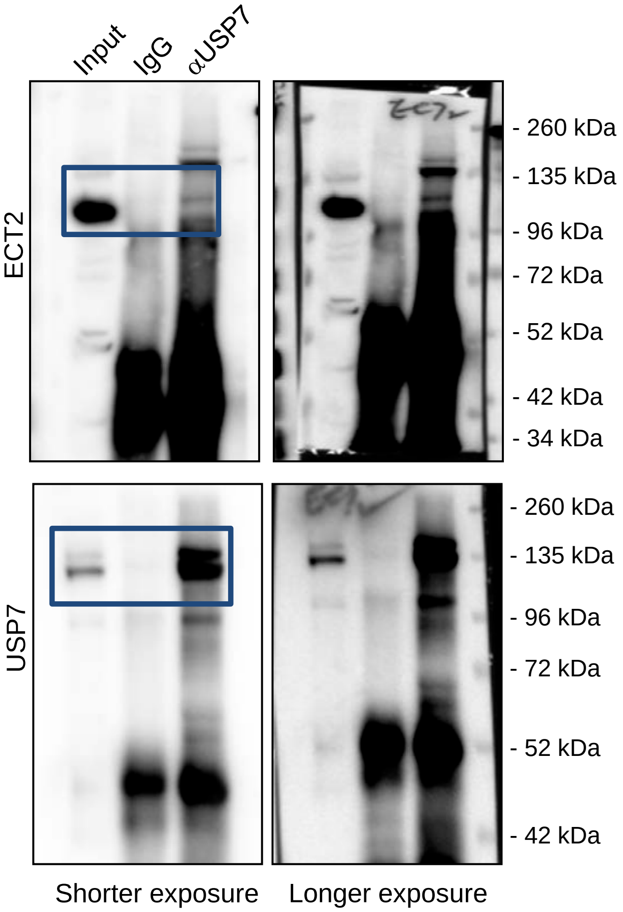

Right panel

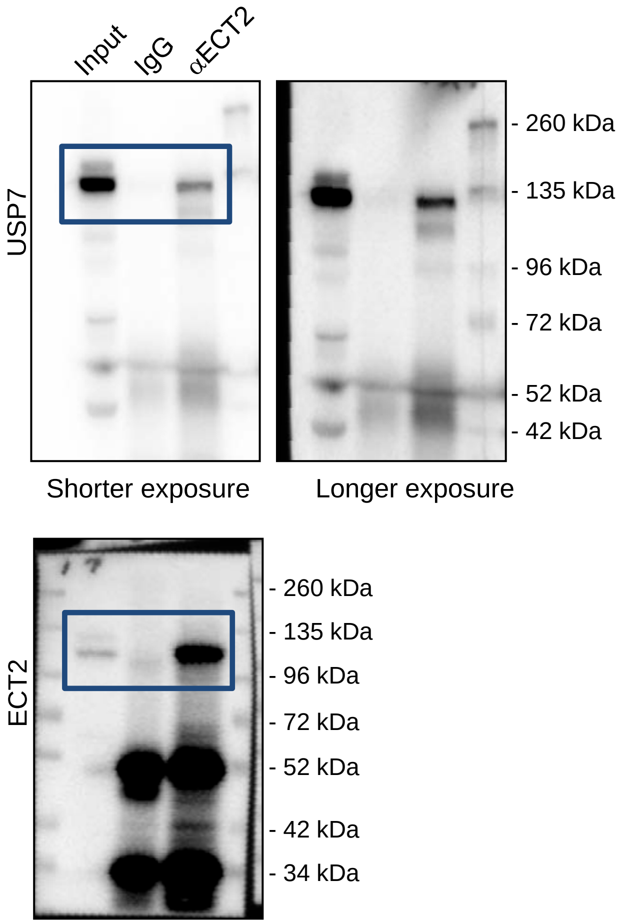

Full unedited gel for Figure 2C

upper panel

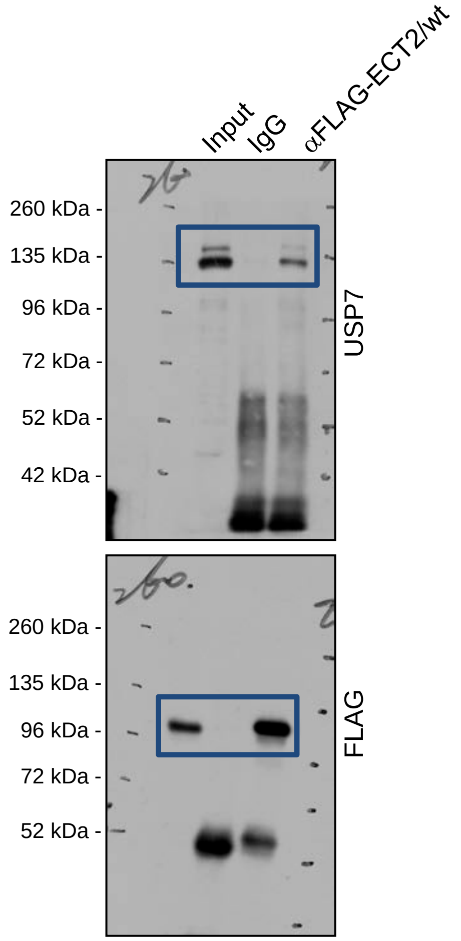

lower panel

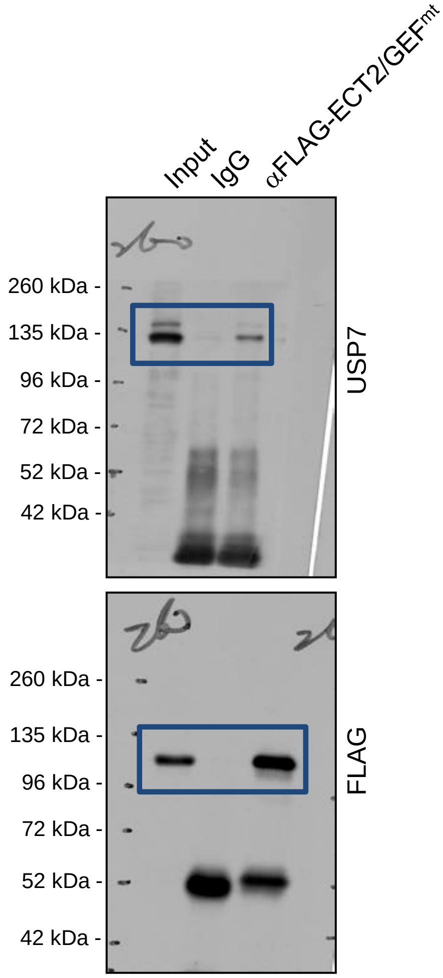

Full unedited gel for Figure 2F

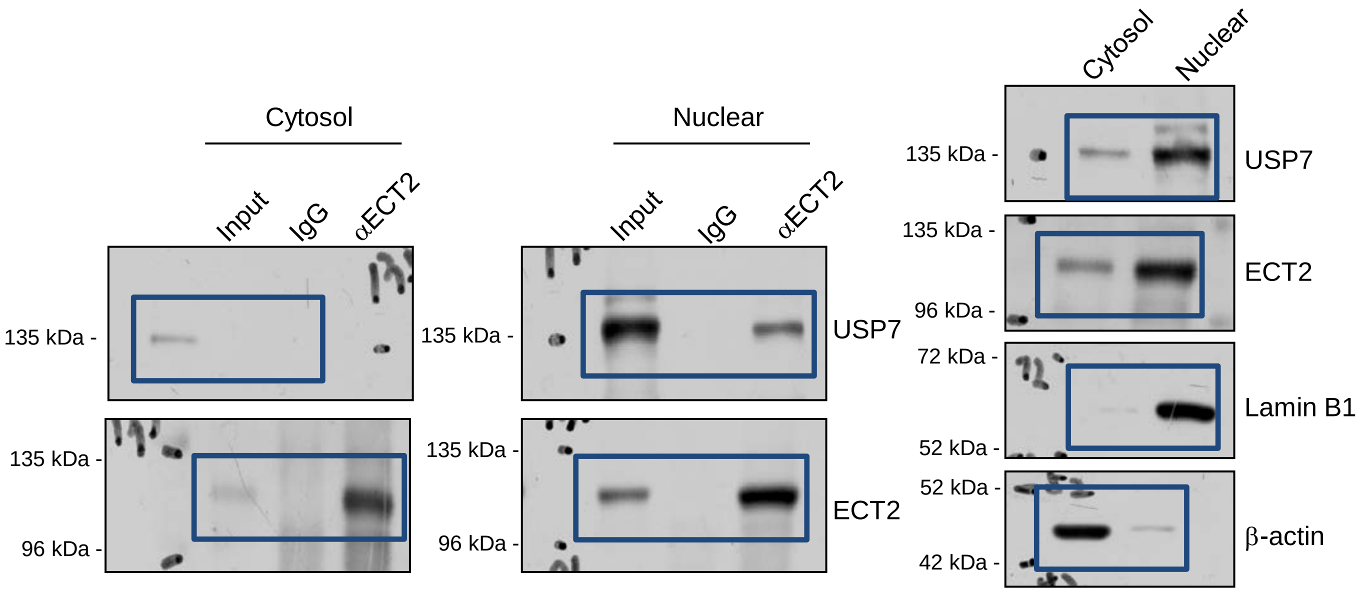

Full unedited gel for Figure 2G

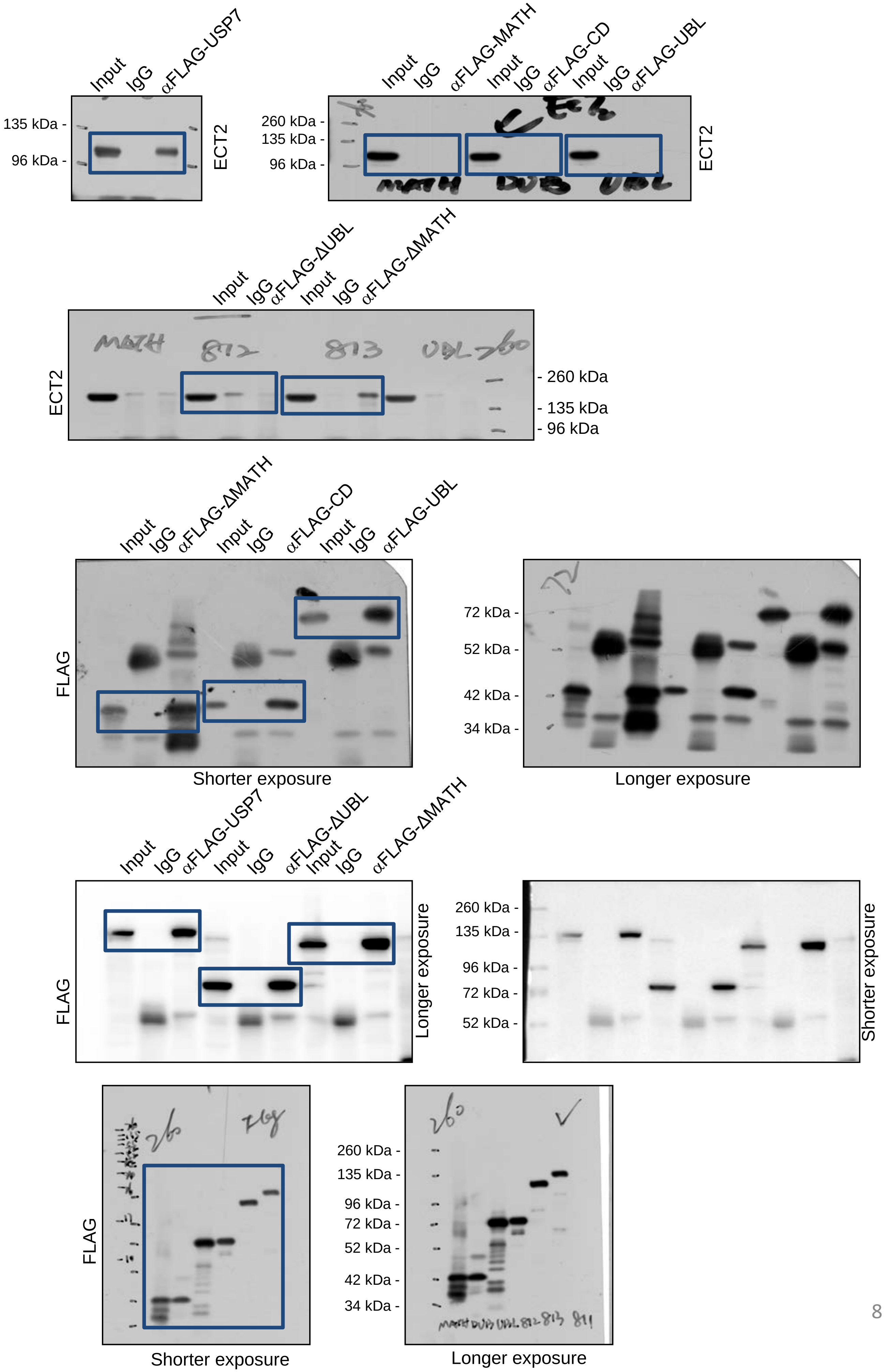

Full unedited gel for Figure 2H

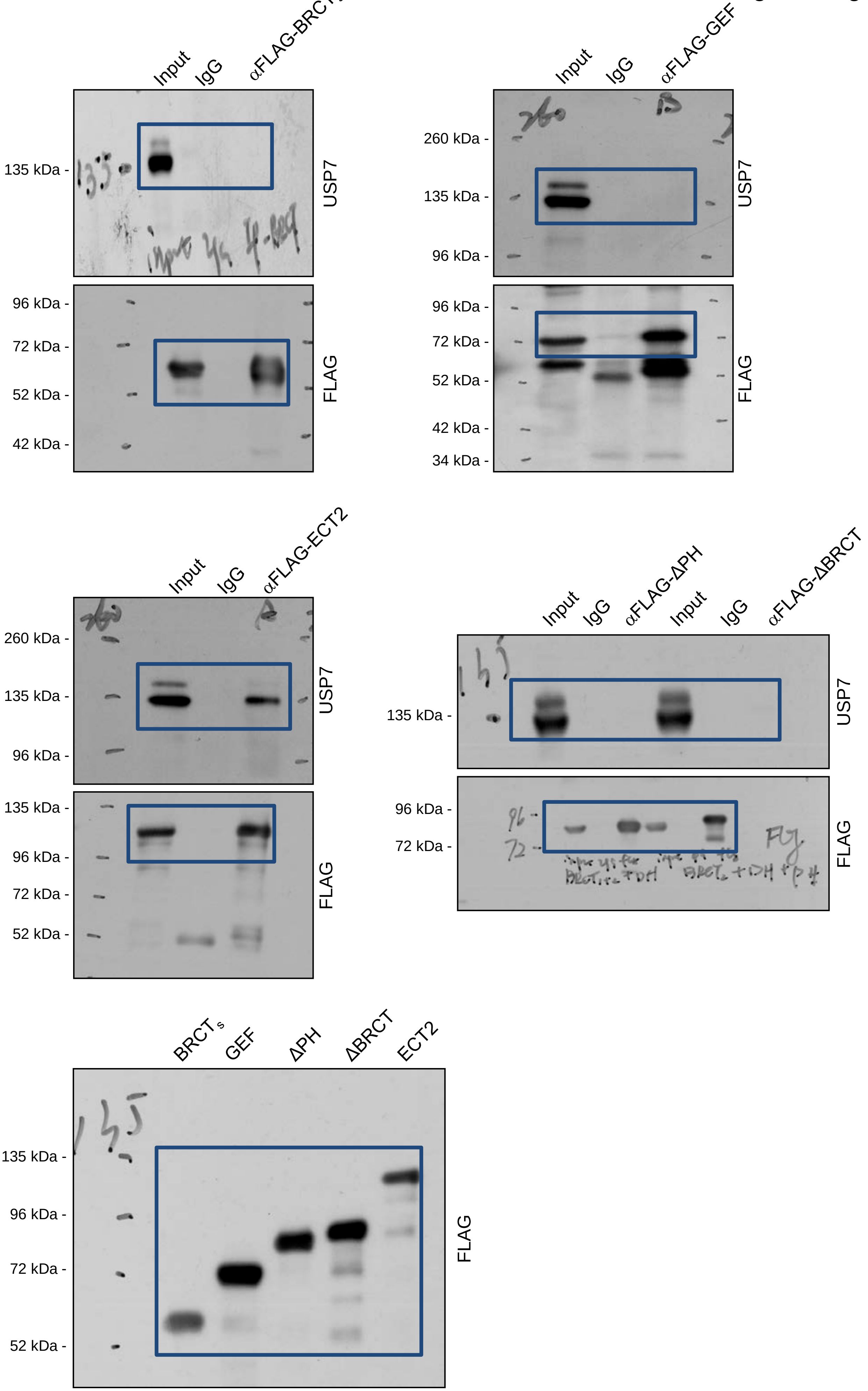

Full unedited gel for Figure 2I

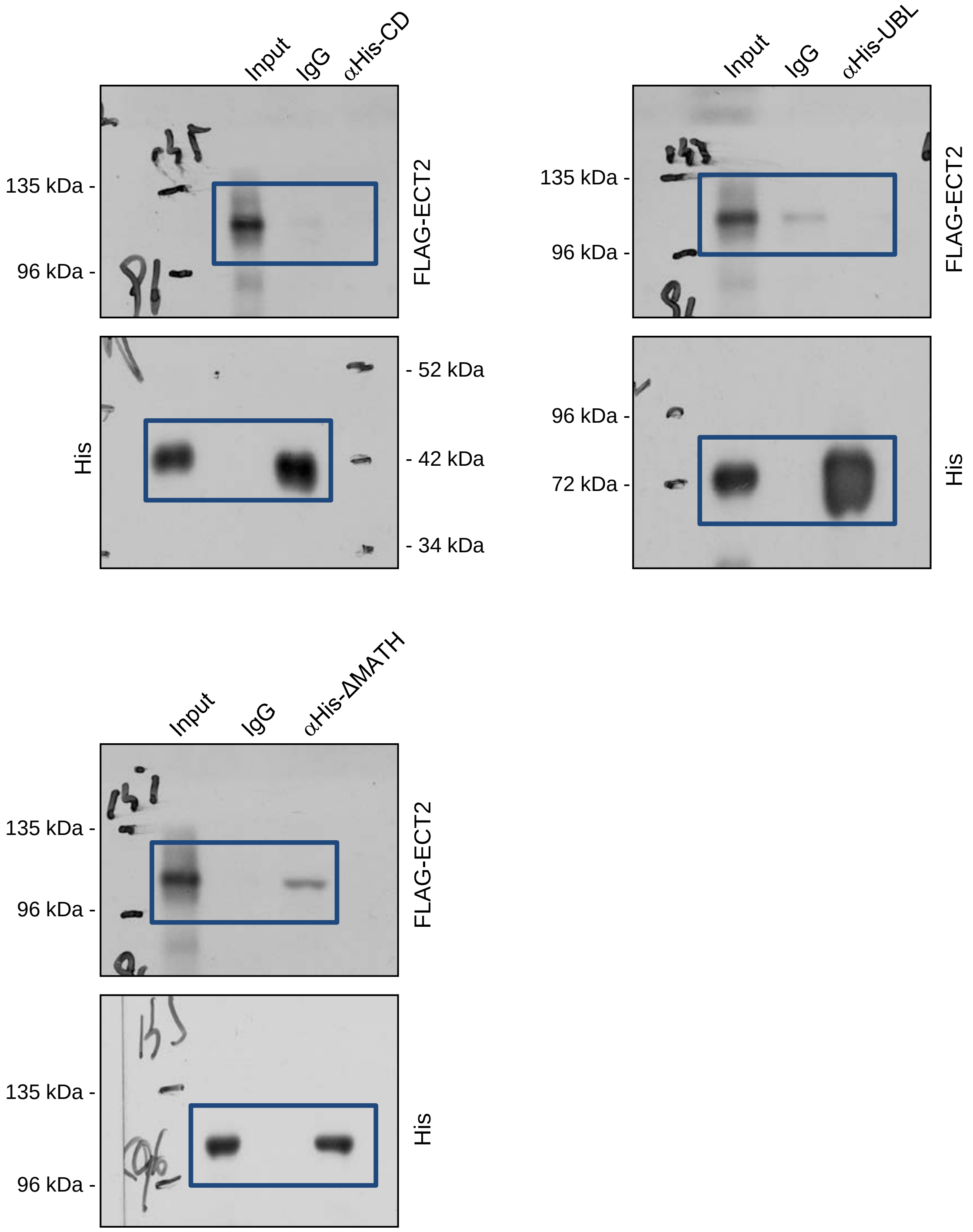

Full unedited gel for Figure 3A

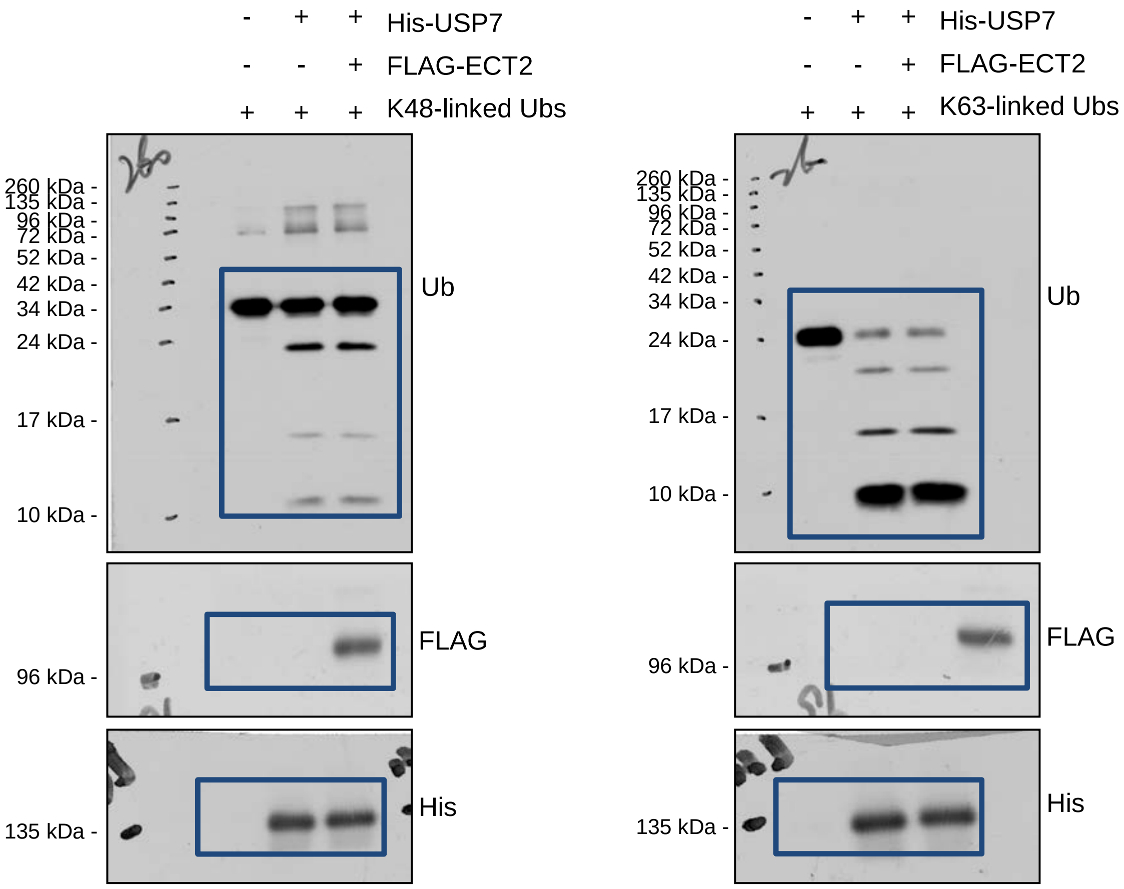

Full unedited gel for Figure 3B

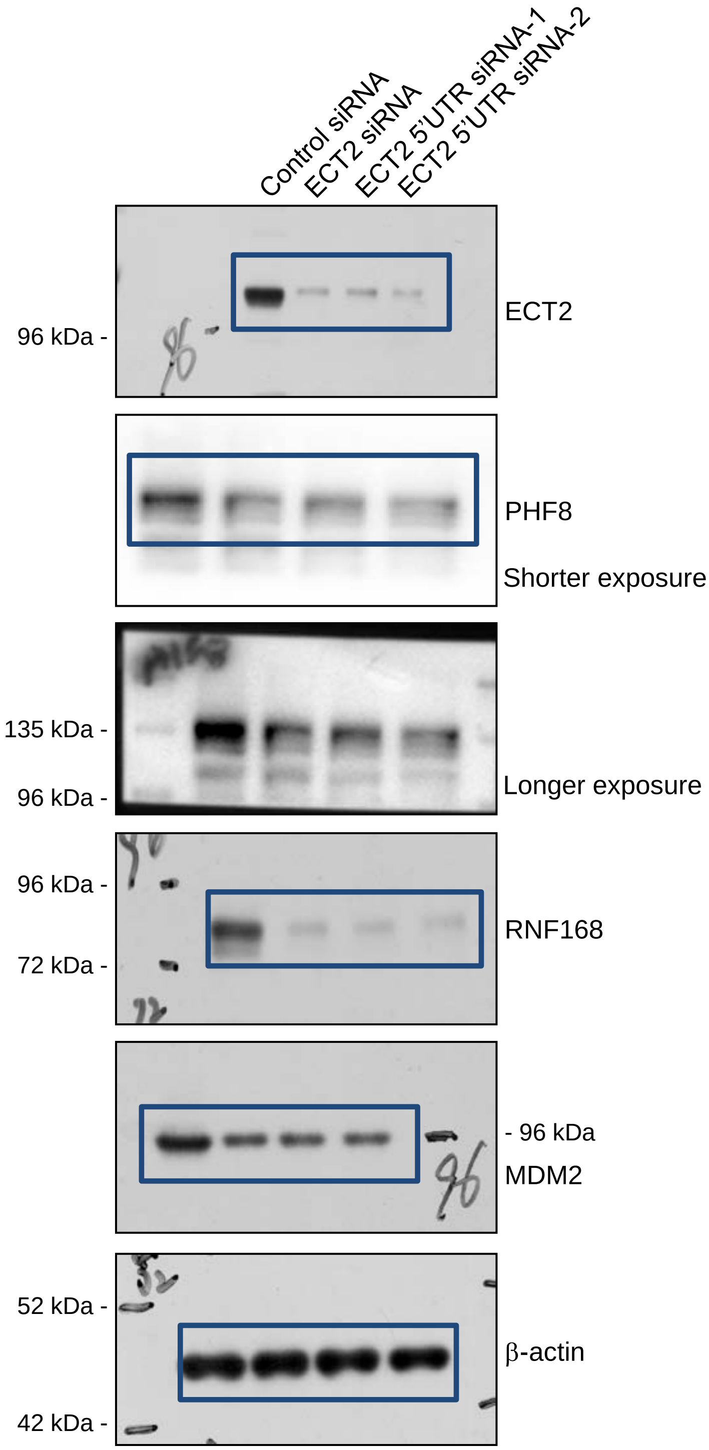

Full unedited gel for Figure 3C

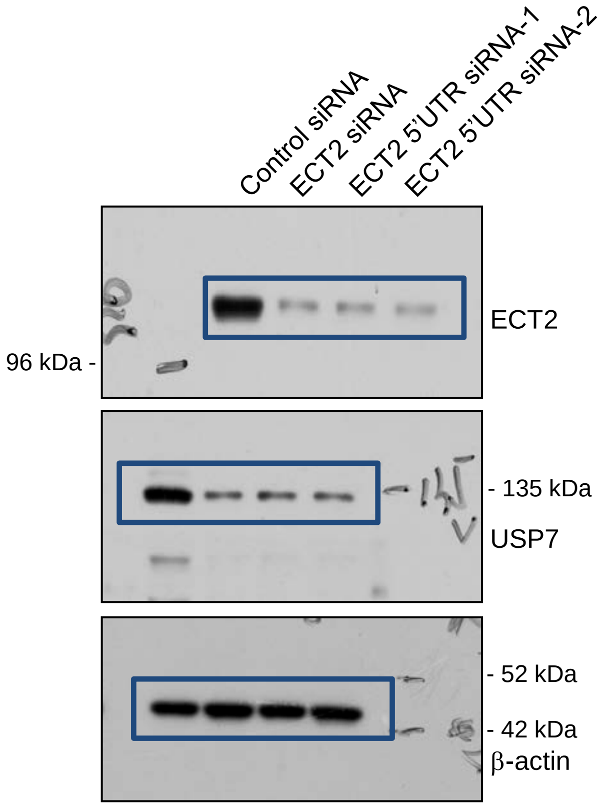

Full unedited gel for Figure 3D

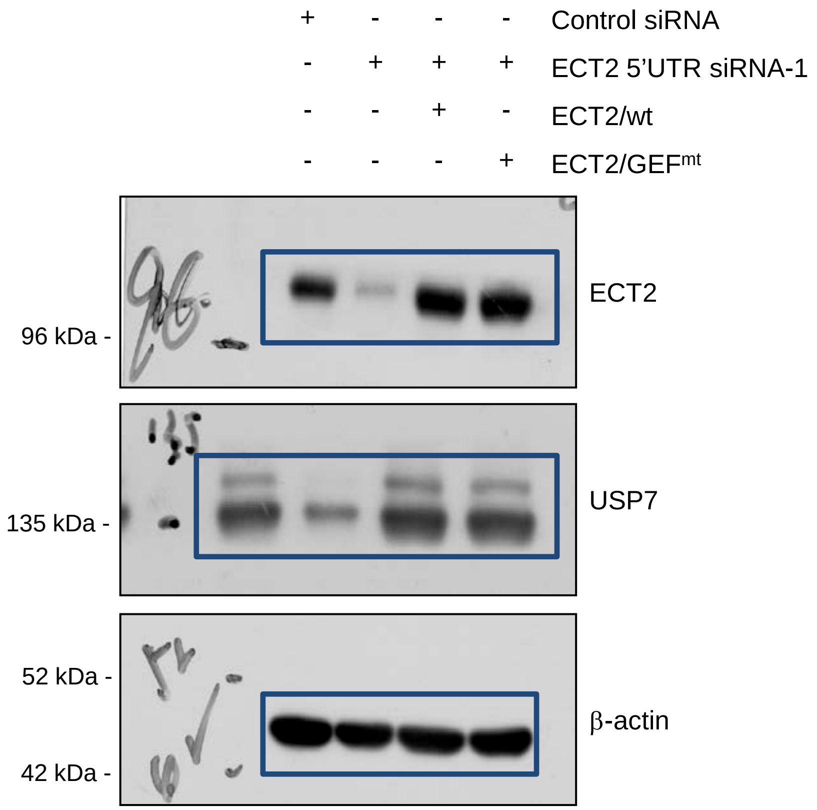

Full unedited gel for Figure 3E

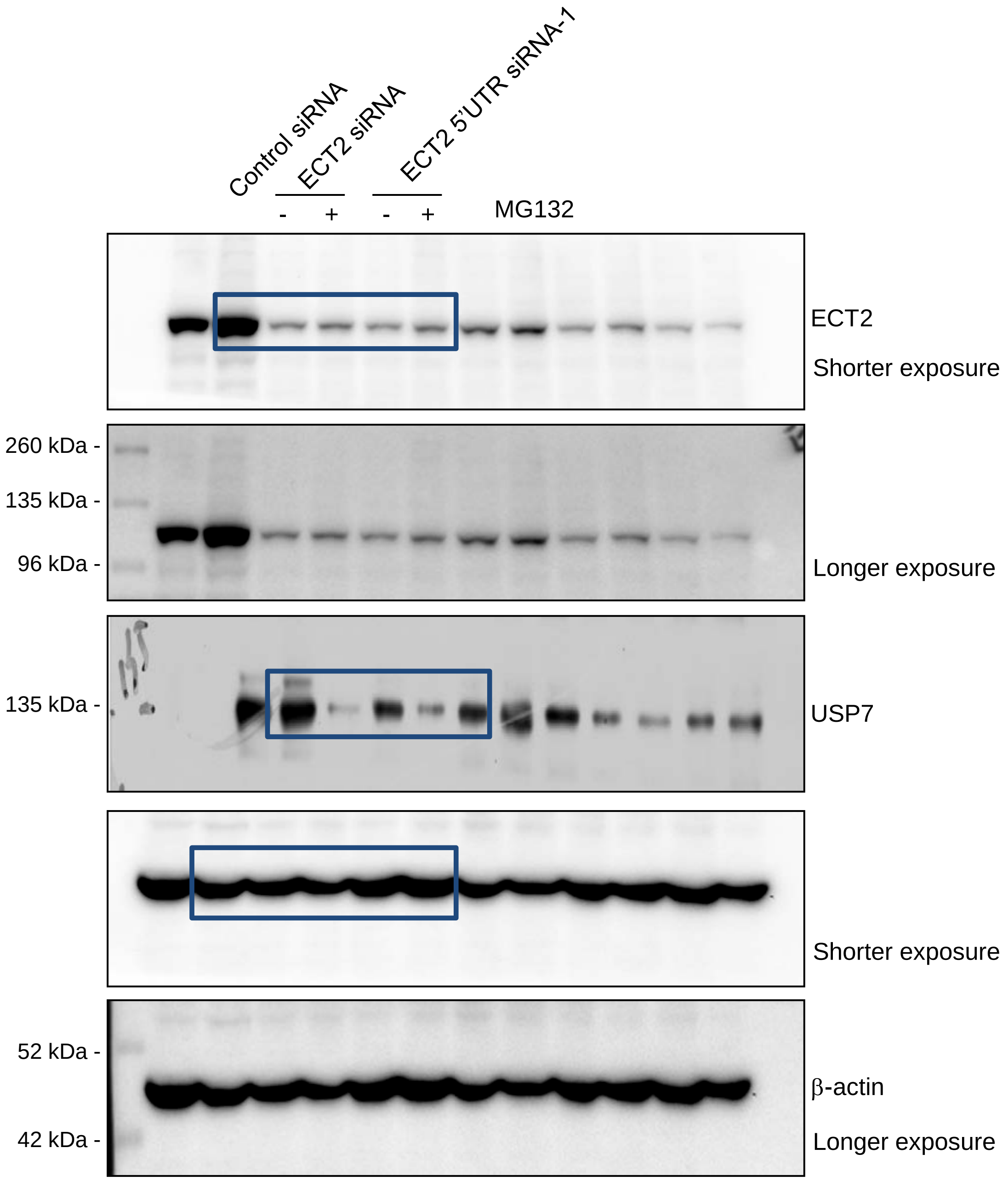

Full unedited gel for Figure 3F

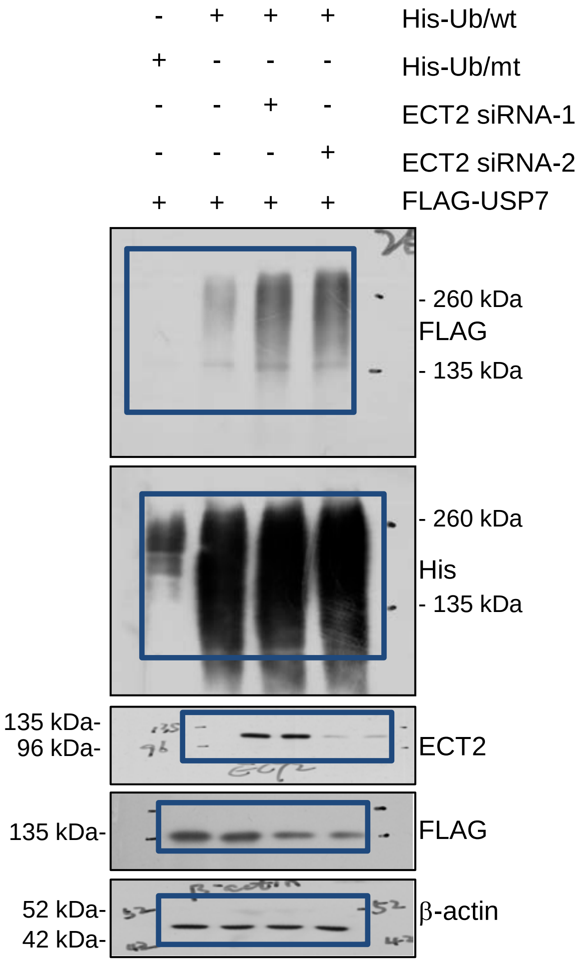

Full unedited gel for Figure 3G

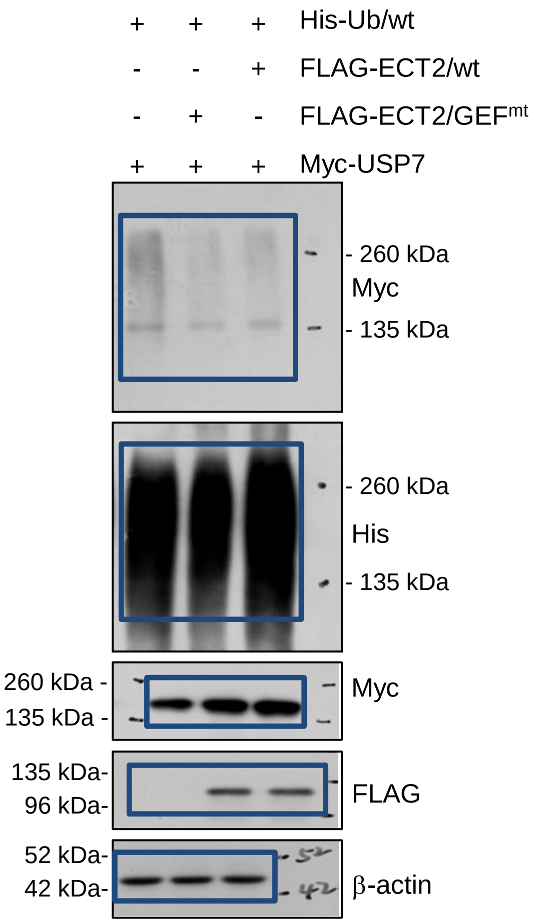

Full unedited gel for Figure 3H left panel

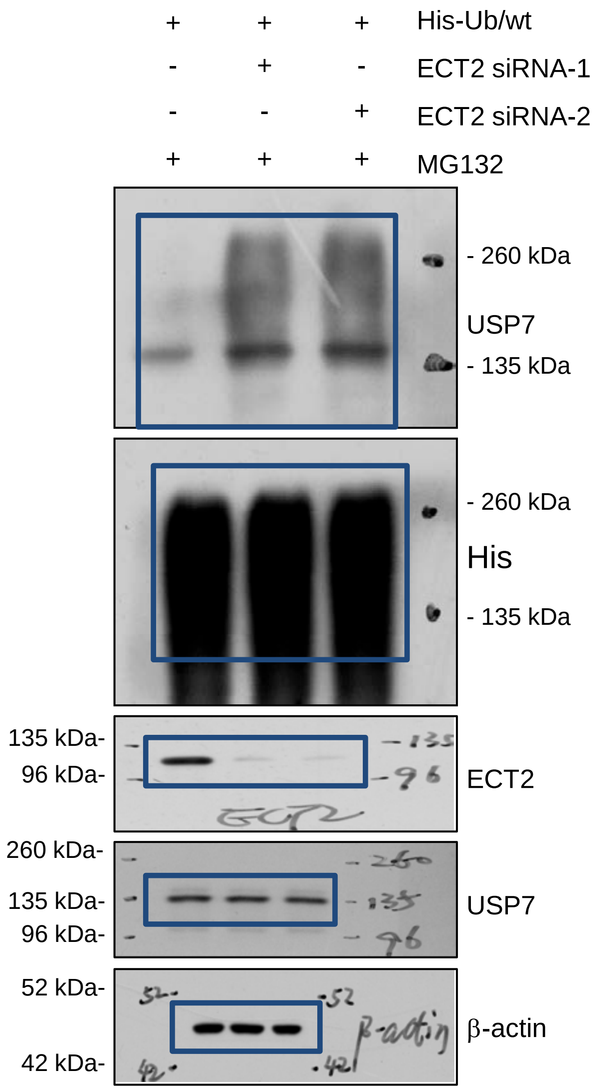

Full unedited gel for Figure 3H right panel

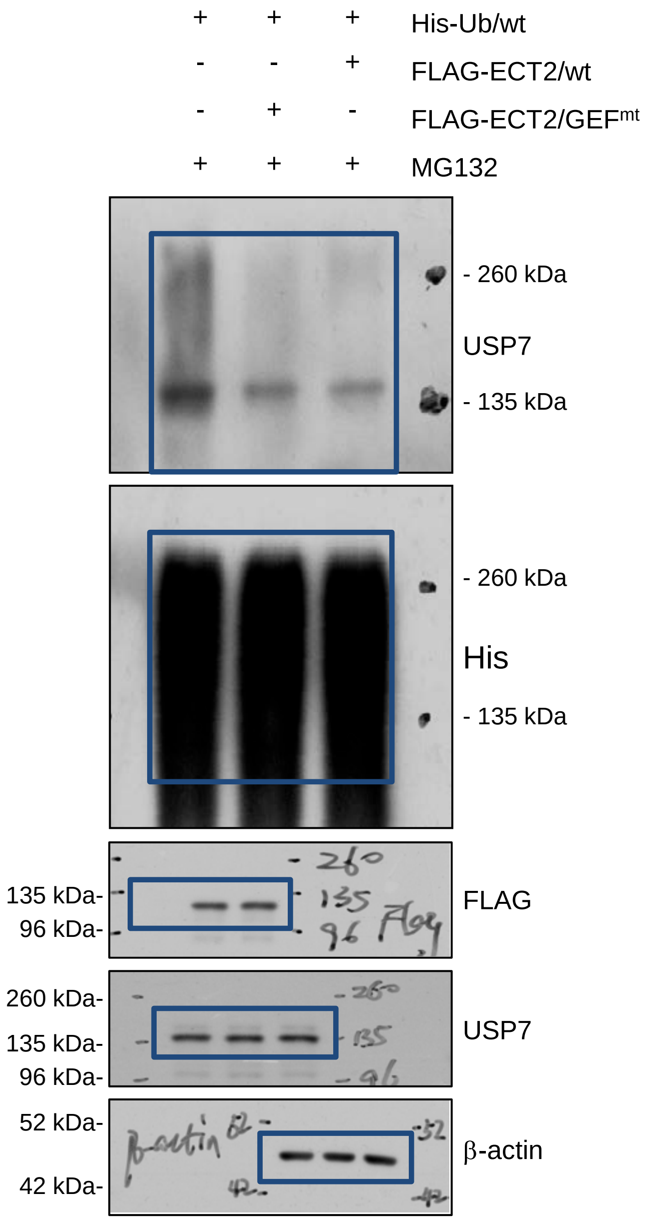

Full unedited gel for Figure 4A

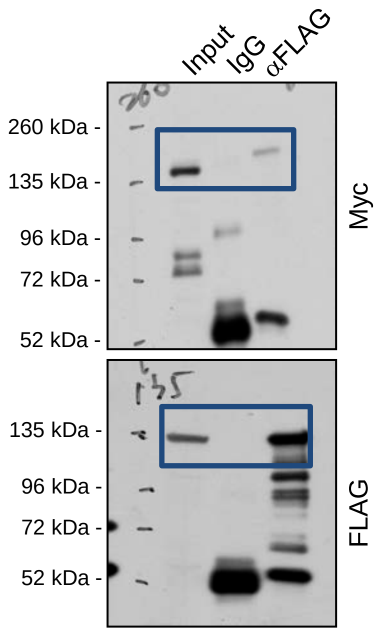

Full unedited gel for Figure 4C

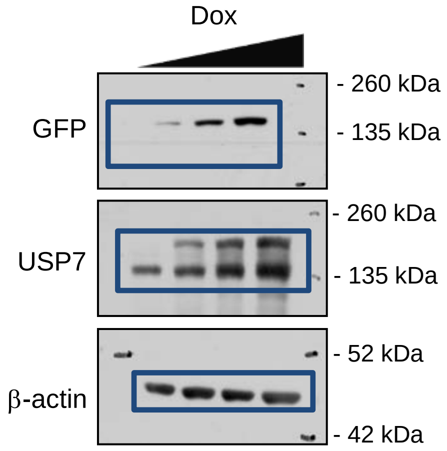

Full unedited gel for Figure 4D

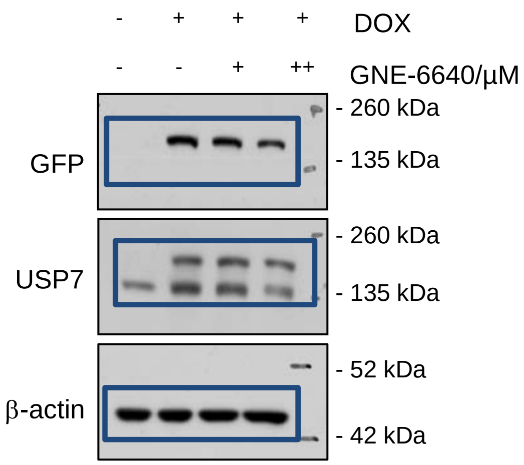

Full unedited gel for Figure 4E

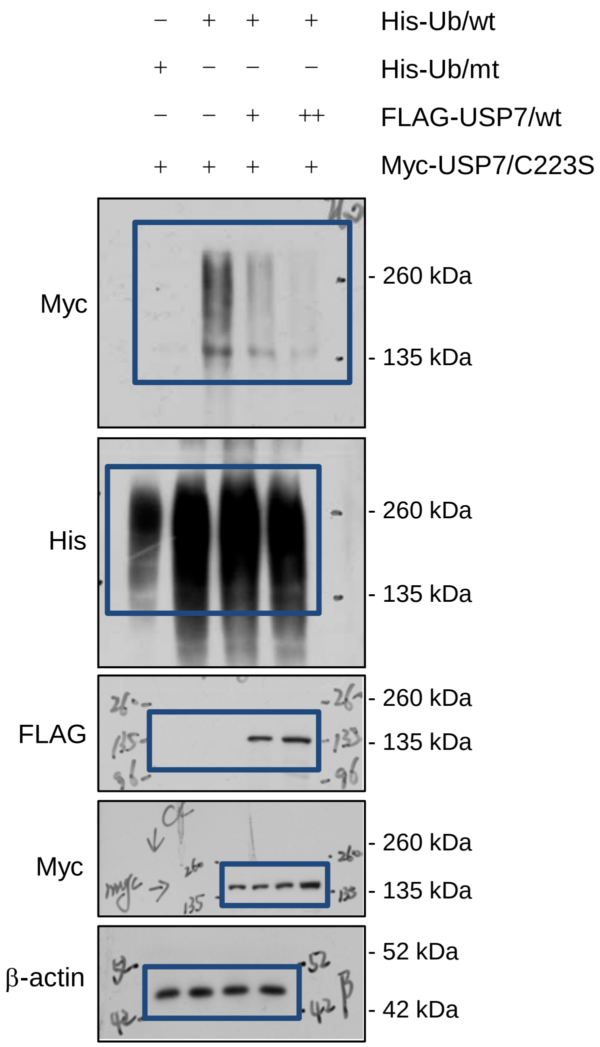

Full unedited gel for Figure 4F

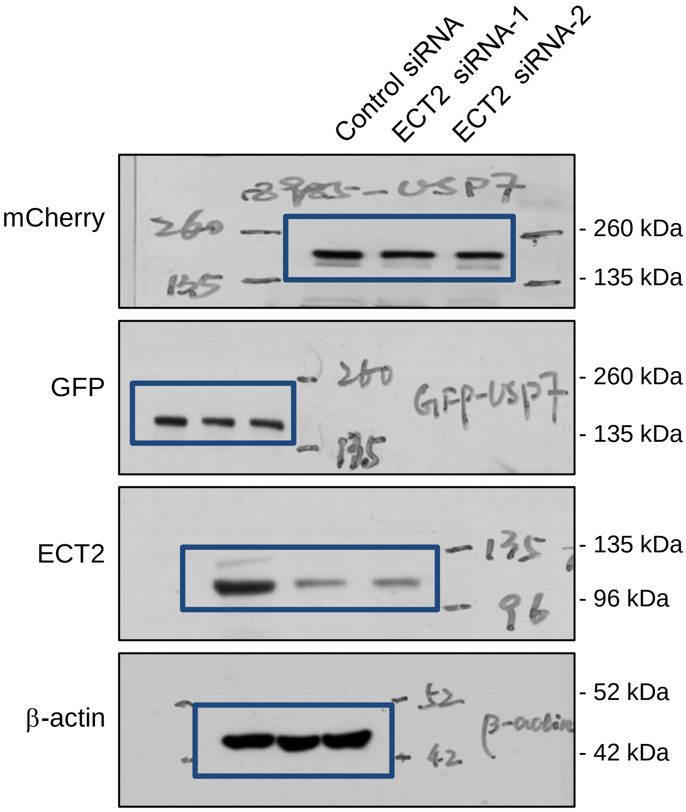

Full unedited gel for Figure 4G

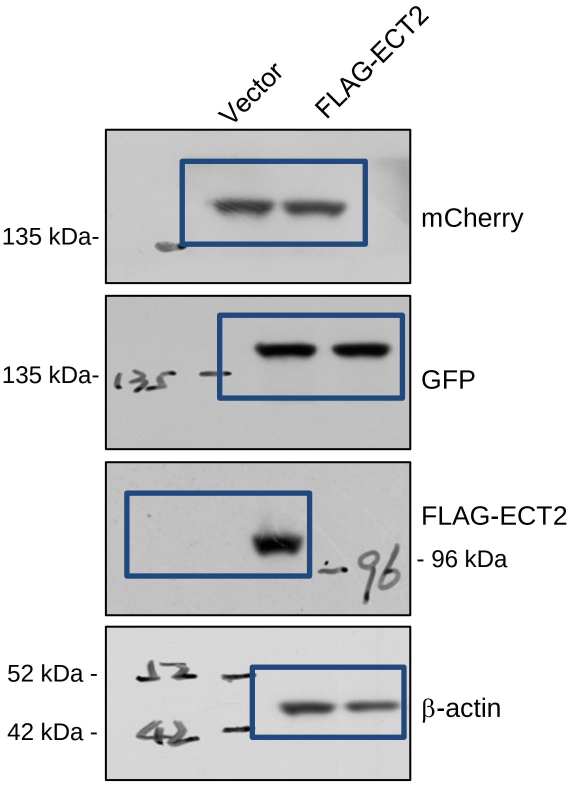

Full unedited gel for Figure 4H

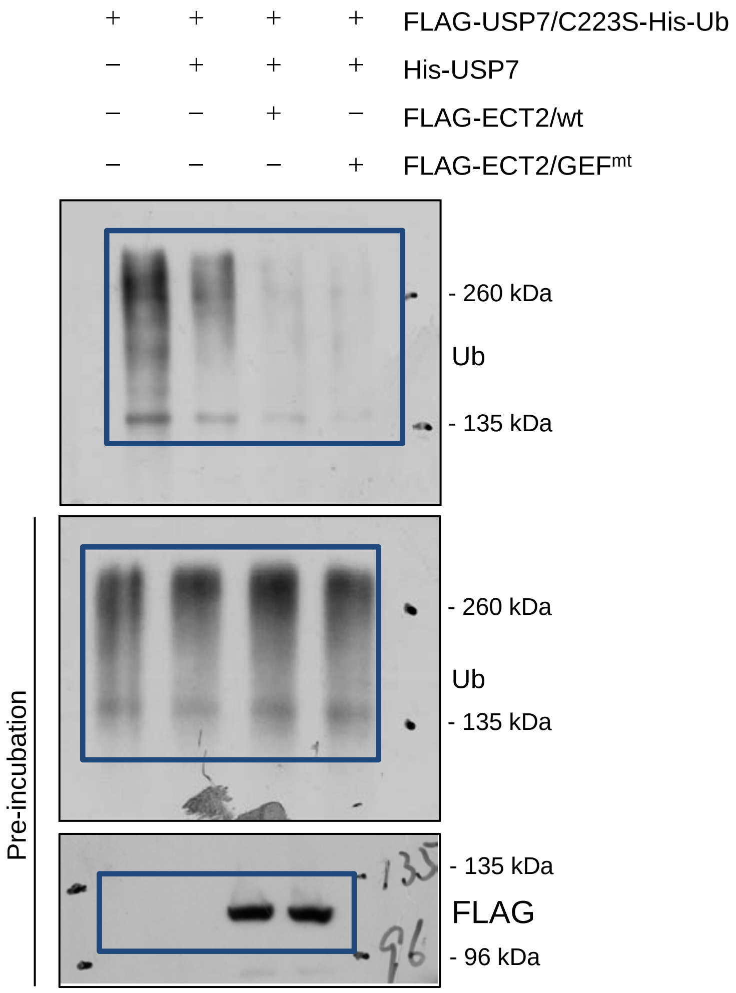

Full unedited gel for Figure 4I

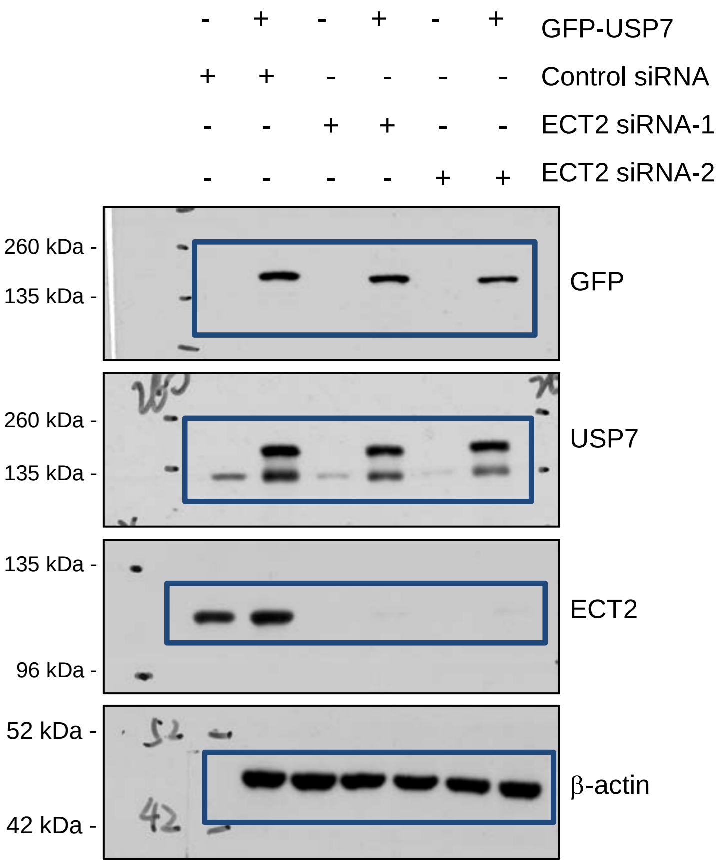

Full unedited gel for Figure 5A

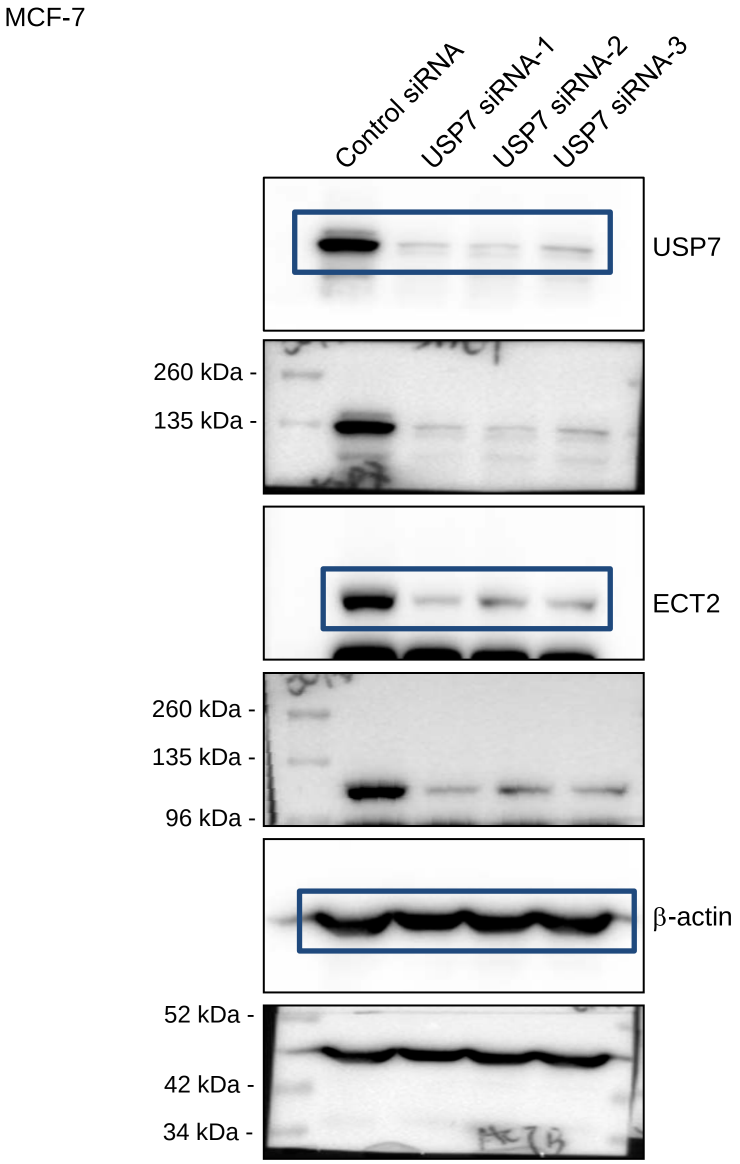

Full unedited gel for Figure 5B

MDA-MB-468

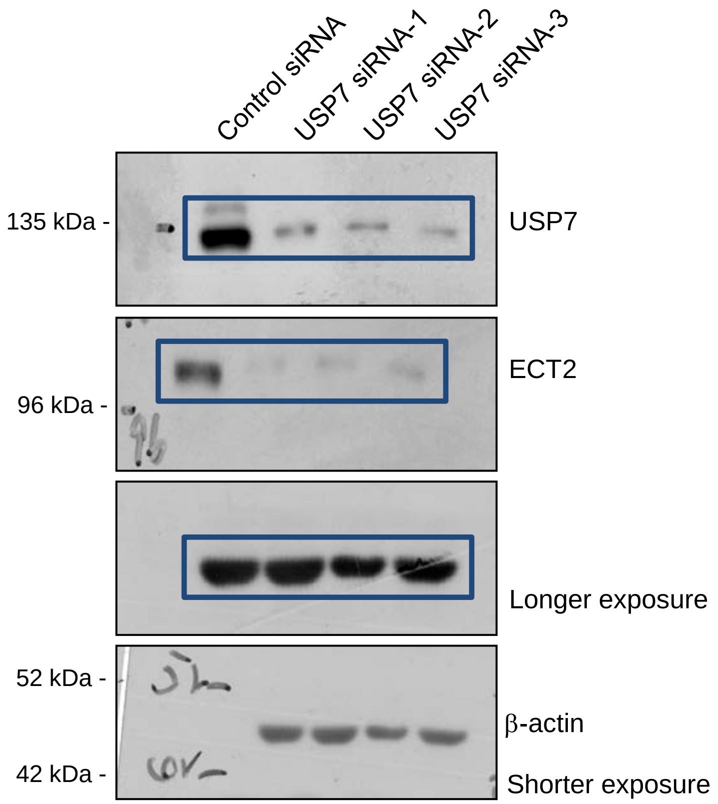

Full unedited gel for Figure 5C

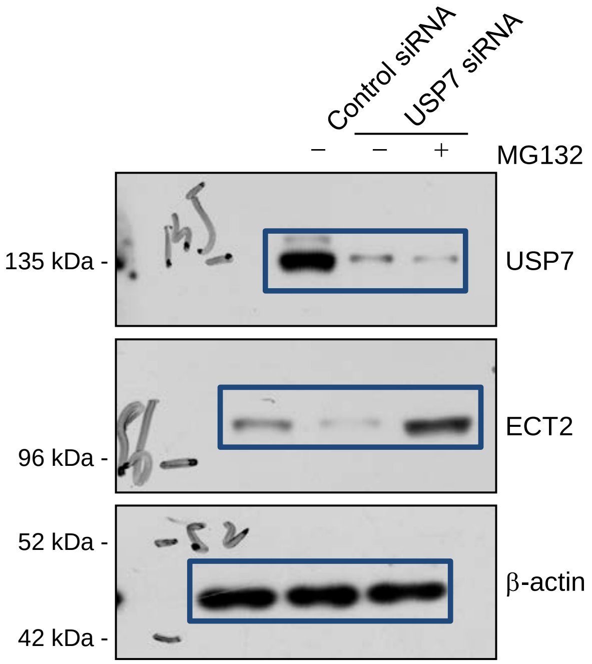

Full unedited gel for Figure 5D

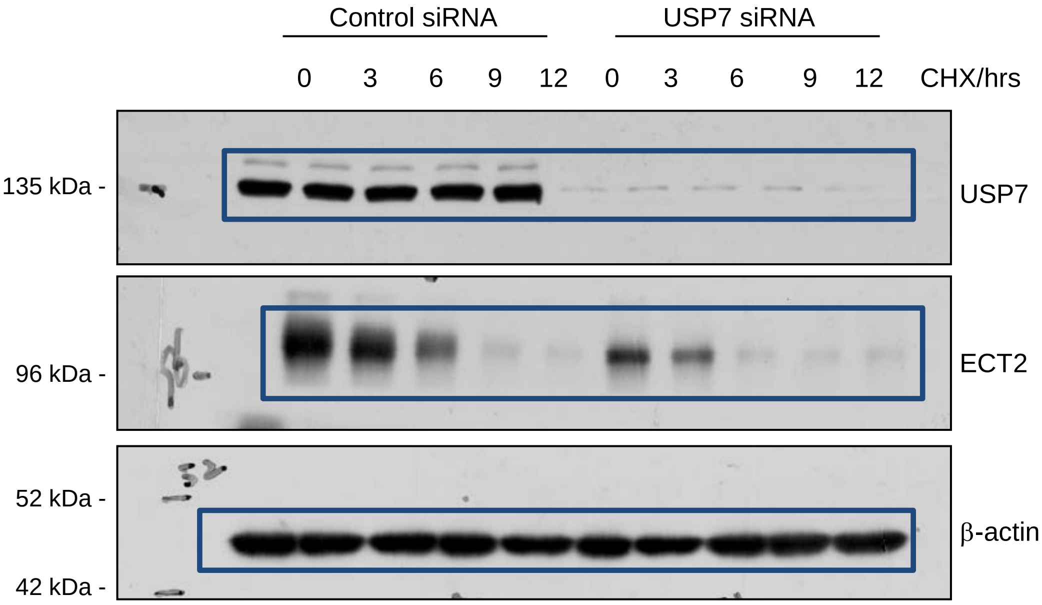

Full unedited gel for Figure 5E

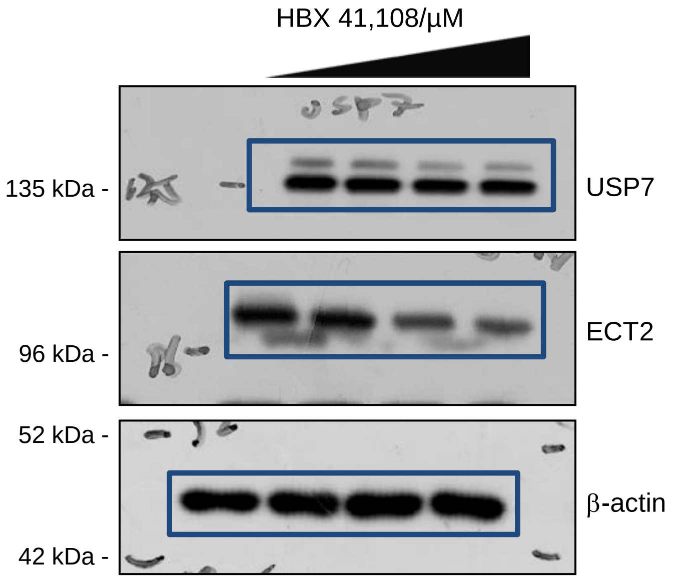

Full unedited gel for Figure 5F

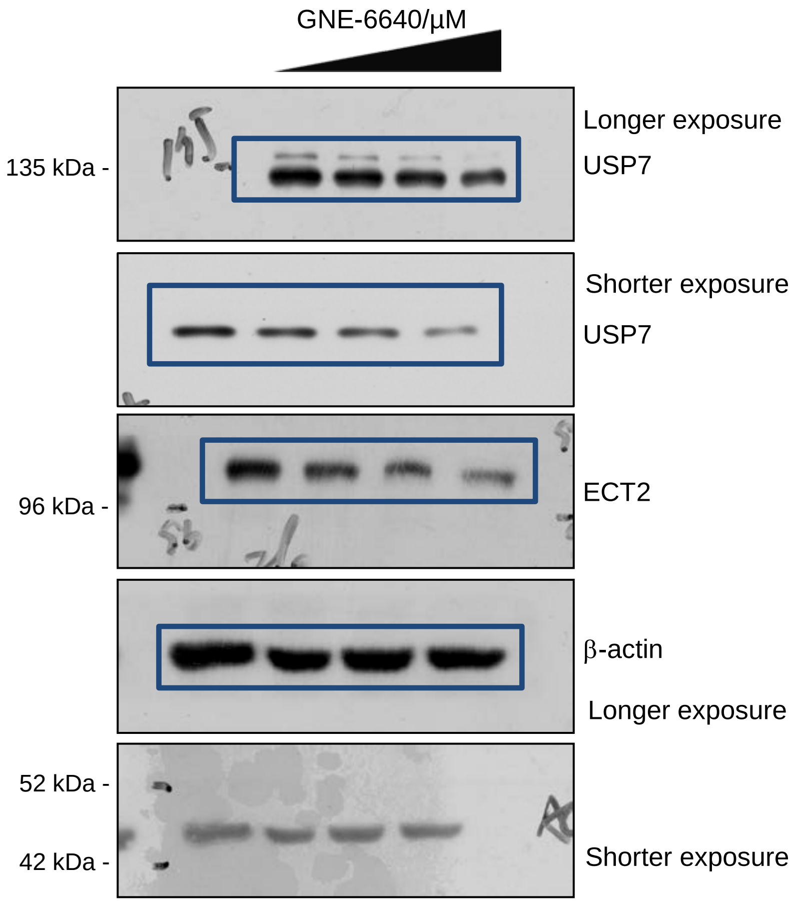

Full unedited gel for Figure 5G

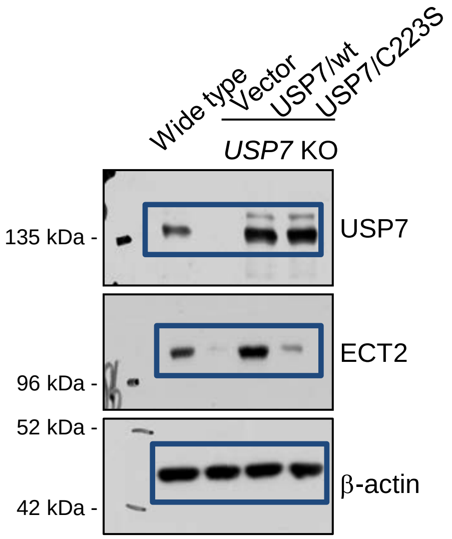

Full unedited gel for Figure 5H

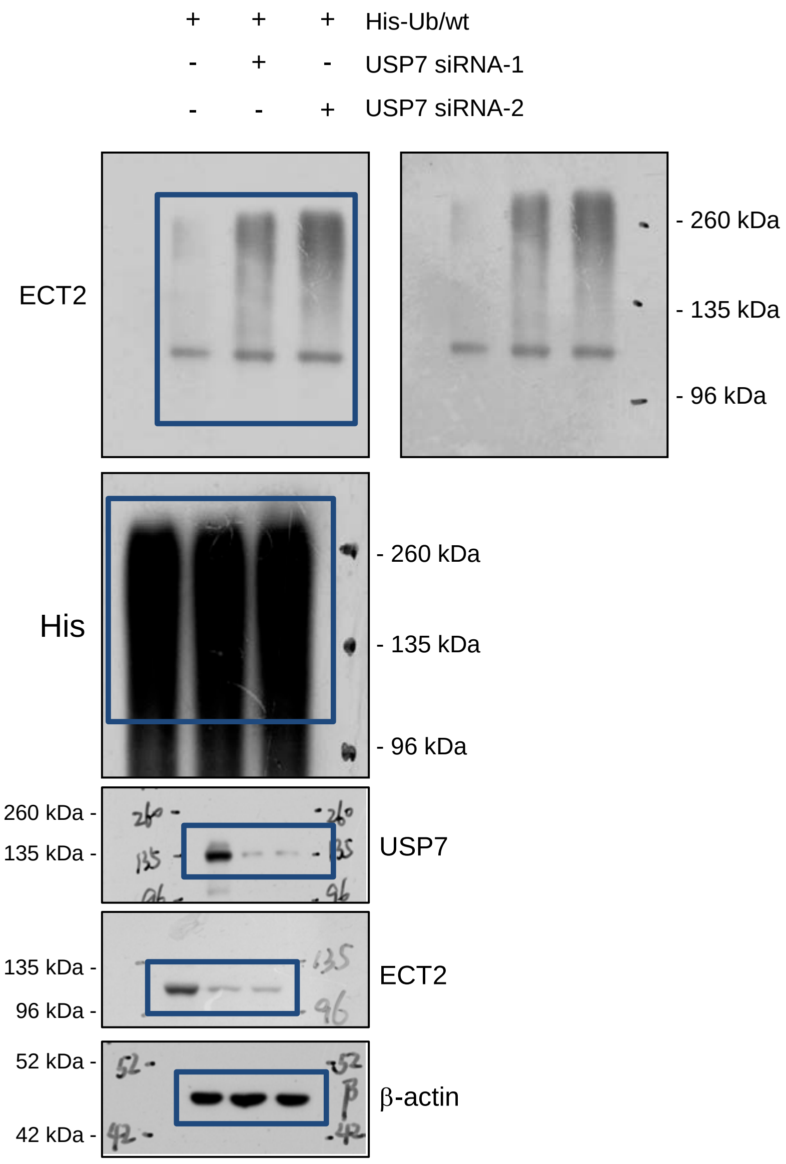

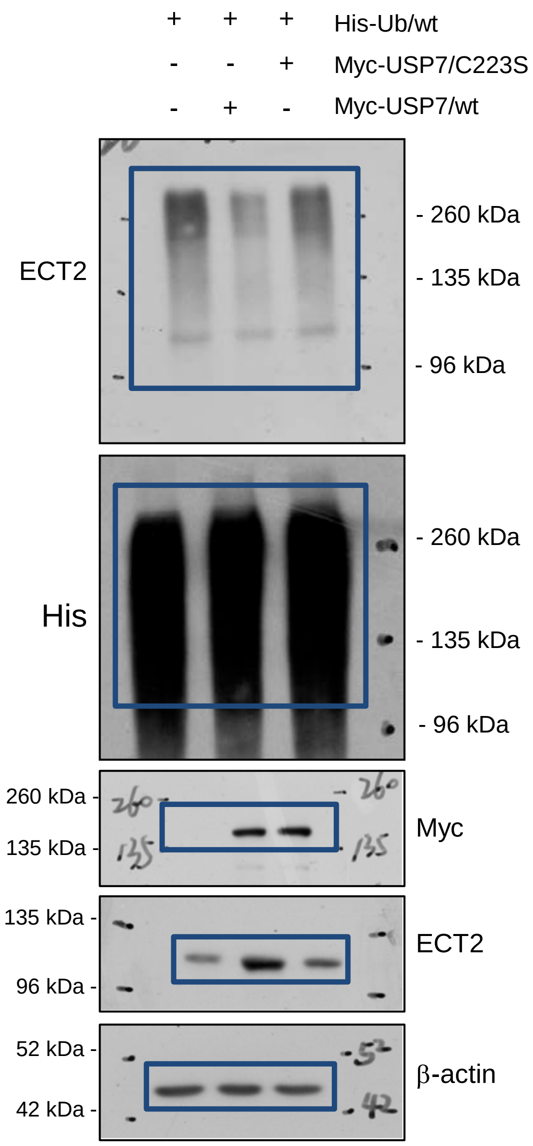

+ + + His-Ub/wt
- - + Myc-USP7/C223S
- + - Myc-USP7/wt
ECT2
- 260 kDa
- 135 kDa
- 96 kDa
His
- 260 kDa
- 135 kDa
- 96 kDa
260 kDa -
135 kDa -
Myc
135 kDa -
96 kDa -
ECT2
52 kDa -
42 kDa -
β-actin

Full unedited gel for Figure 5J

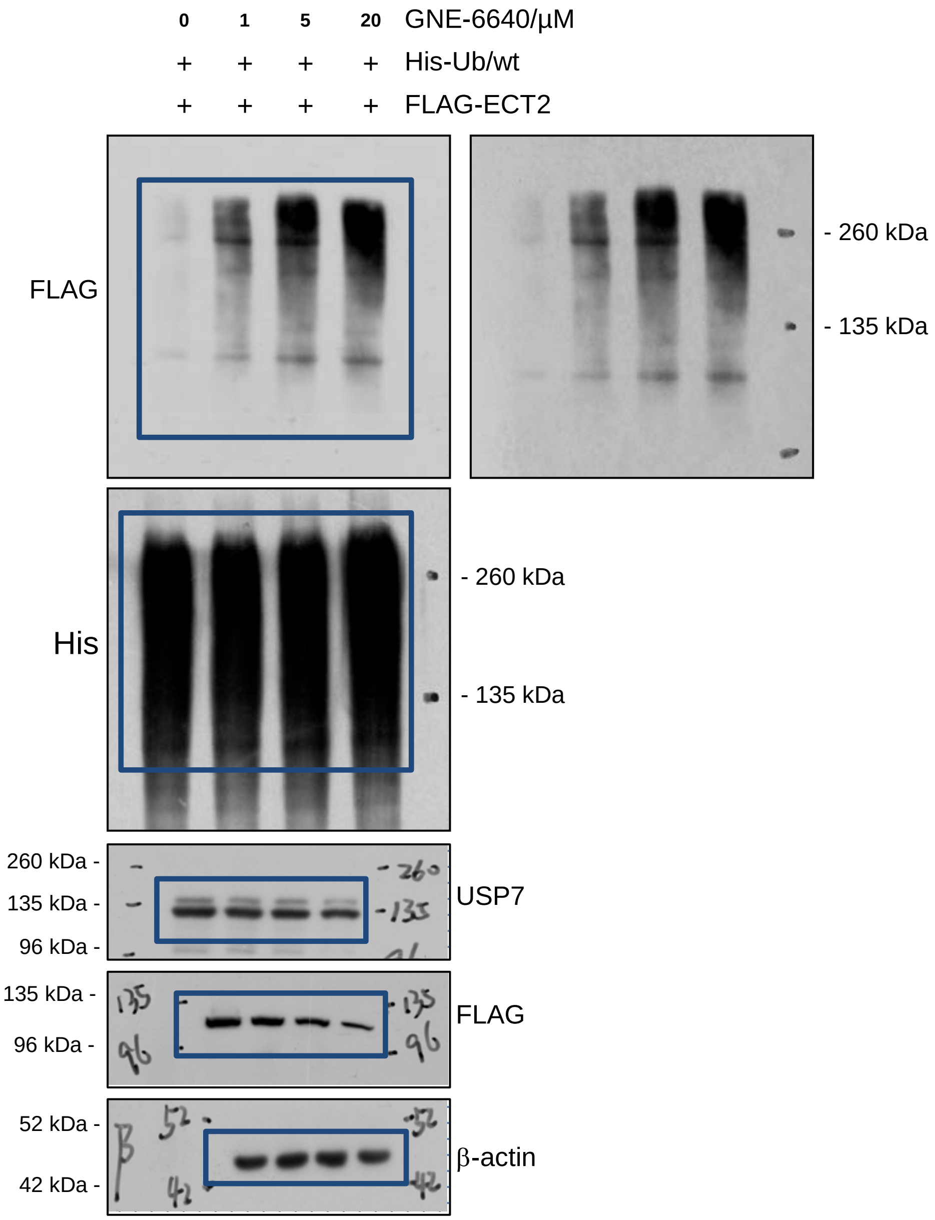

Full unedited gel for Figure 5K

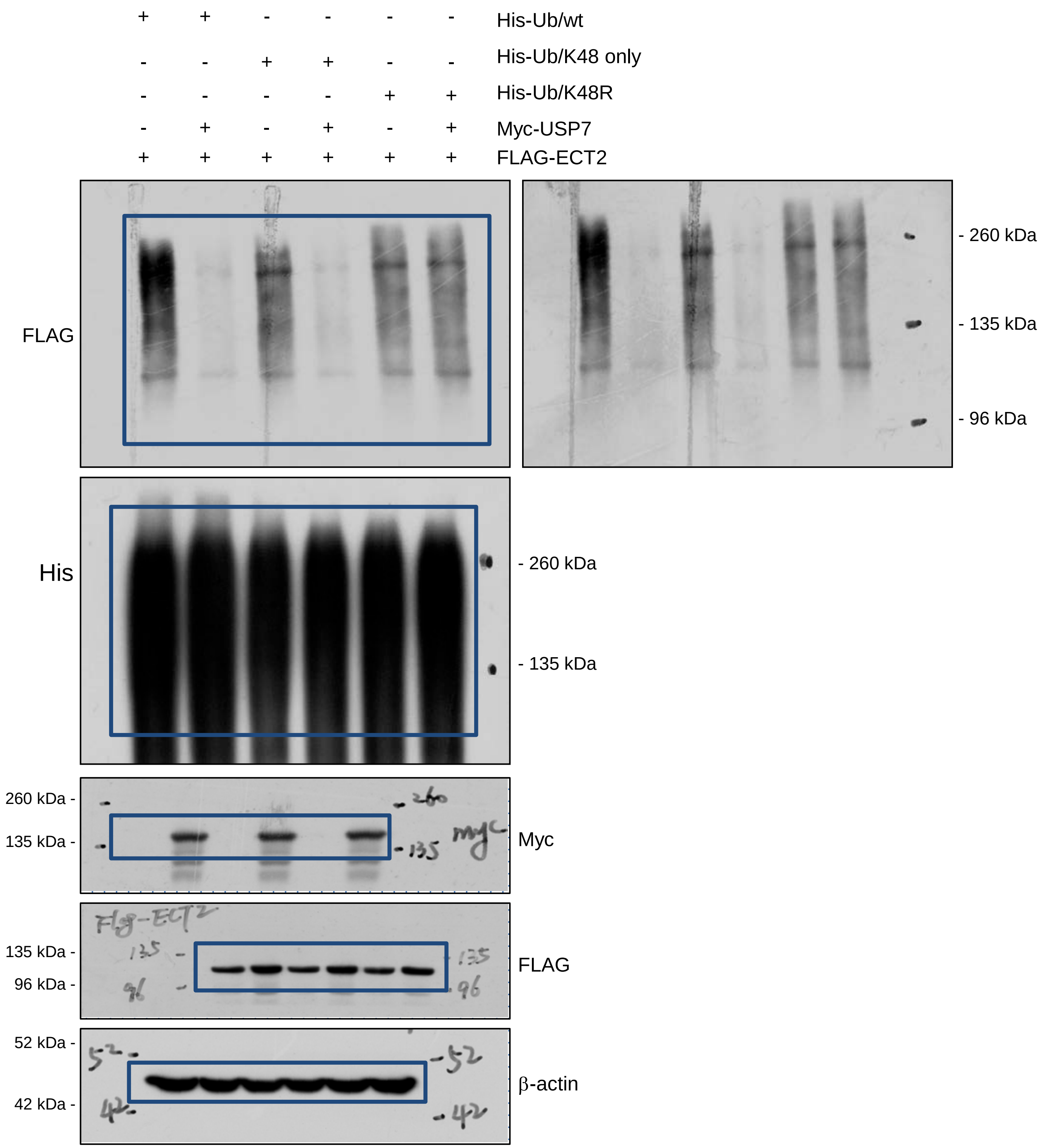

Left panel

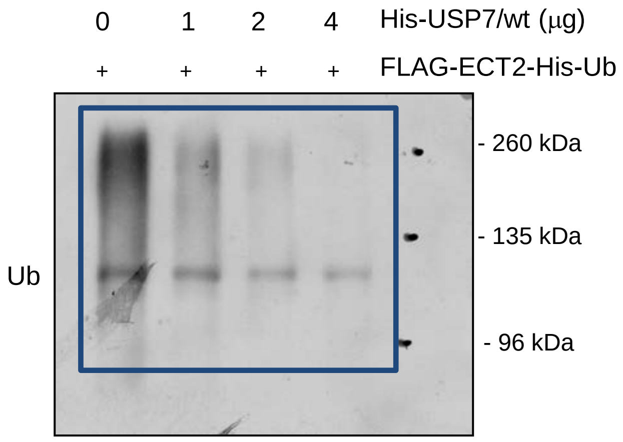

Right panel

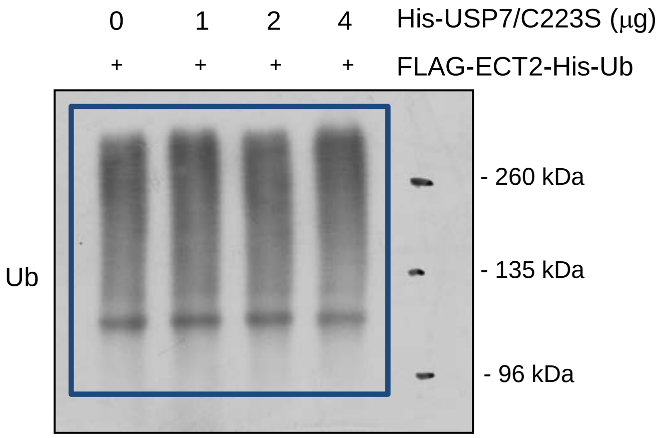

Full unedited gel for Figure 6C

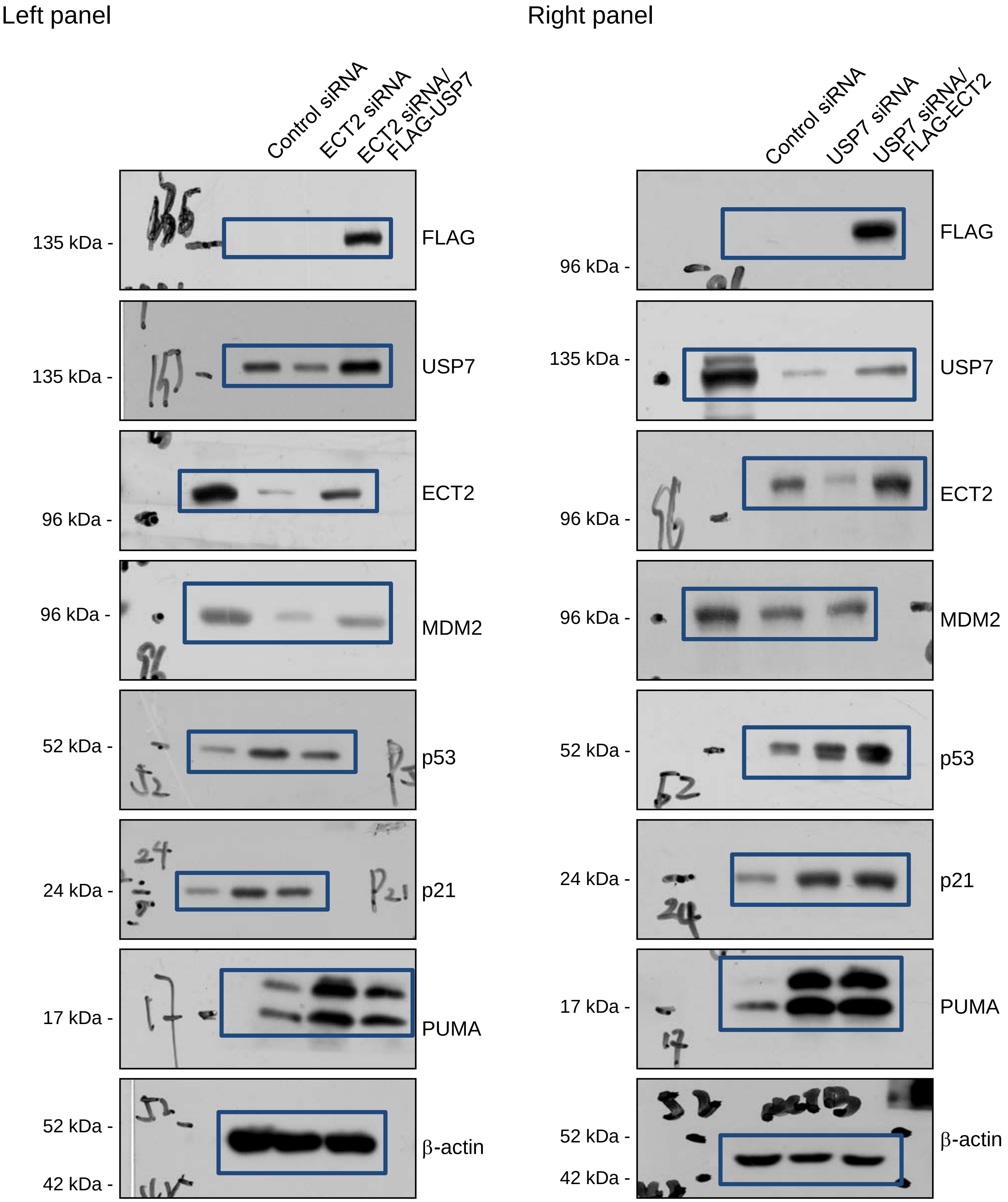

Full unedited gel for Figure 6D

Left panel

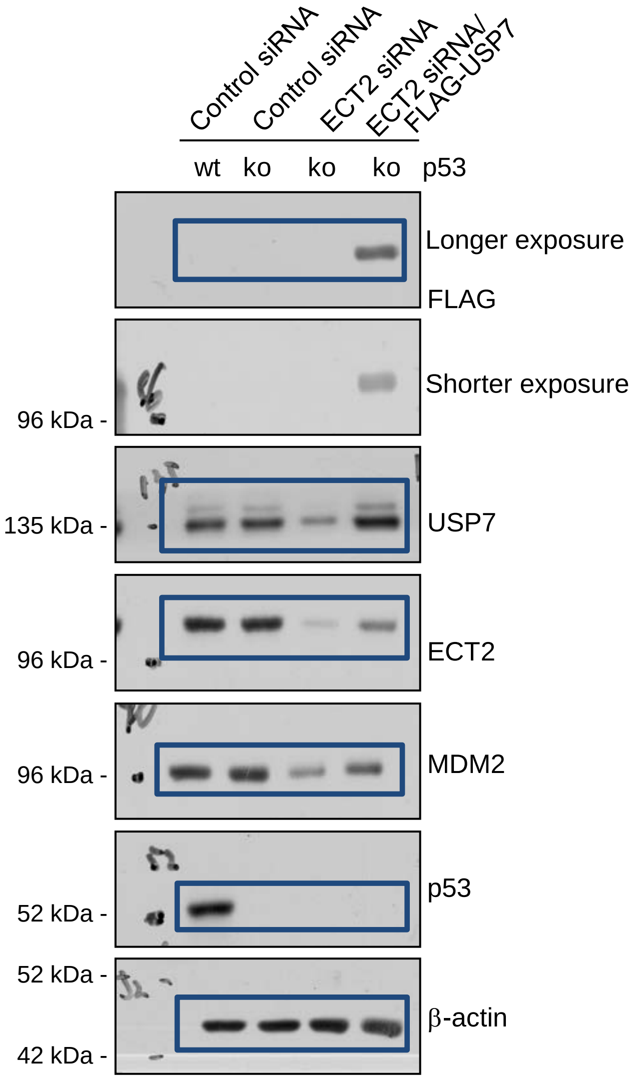

Right panel

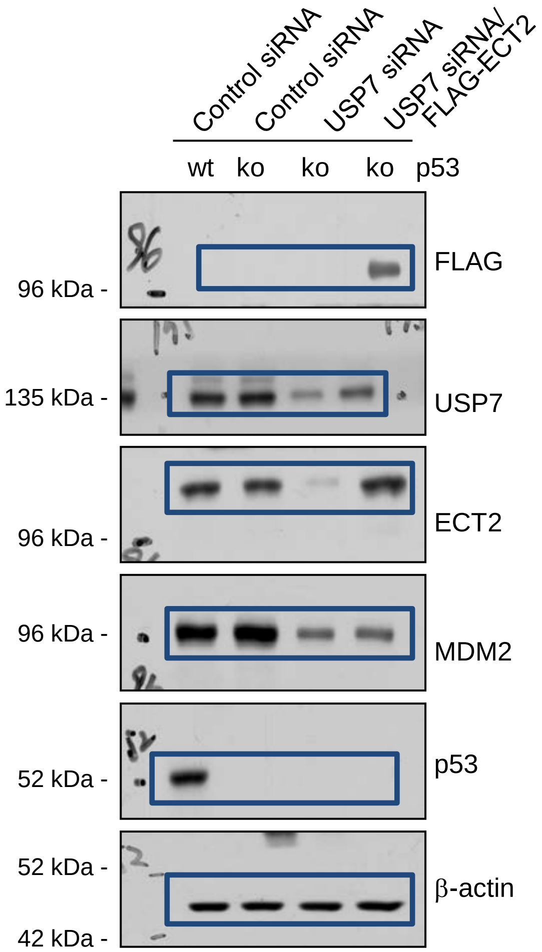

Full unedited gel for Figure 6G

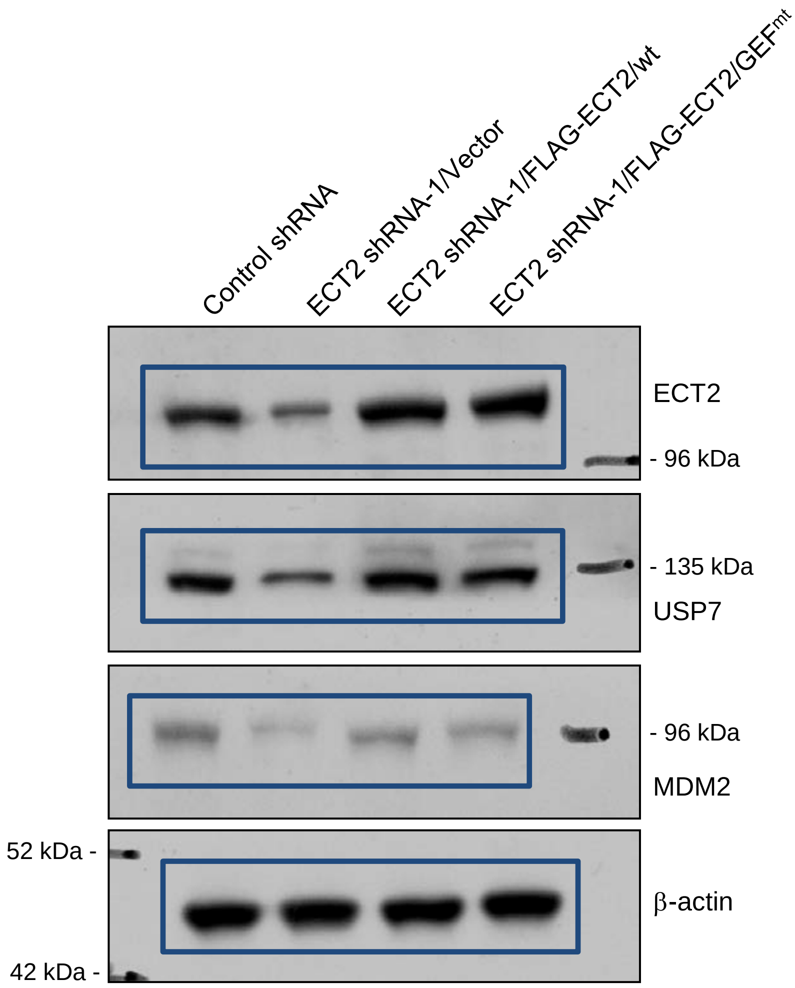

Full unedited gel for Figure S1E

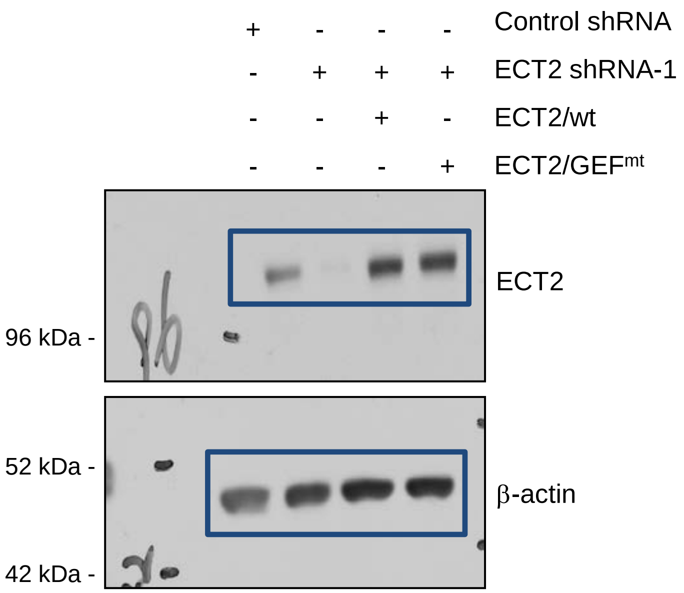

Full unedited gel for Figure S1F

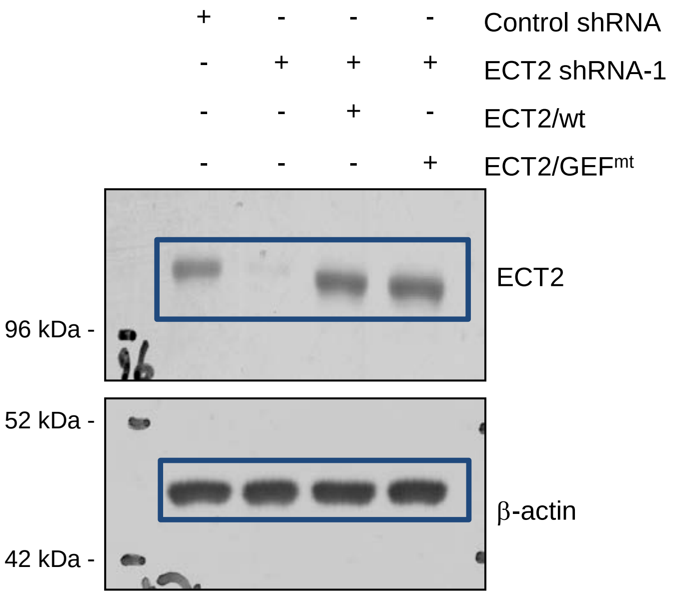

Full unedited gel for Figure S2A

ZR 75-1

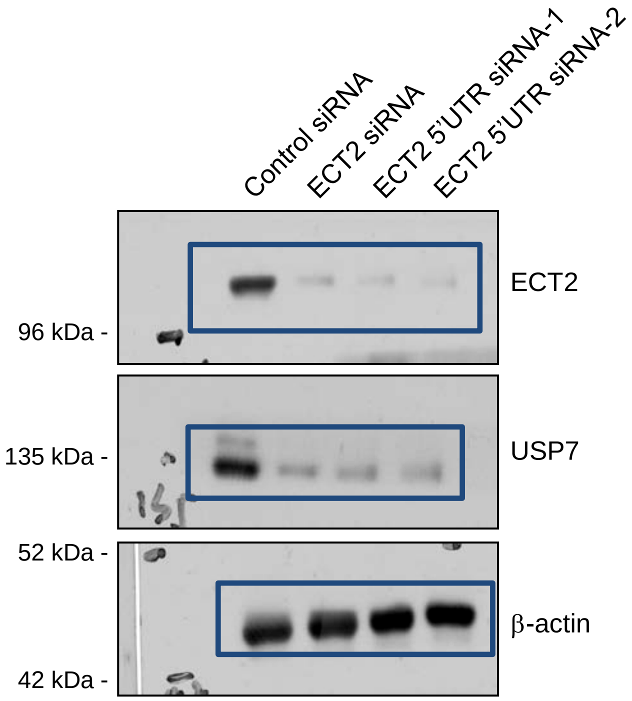

MDA-MB-468

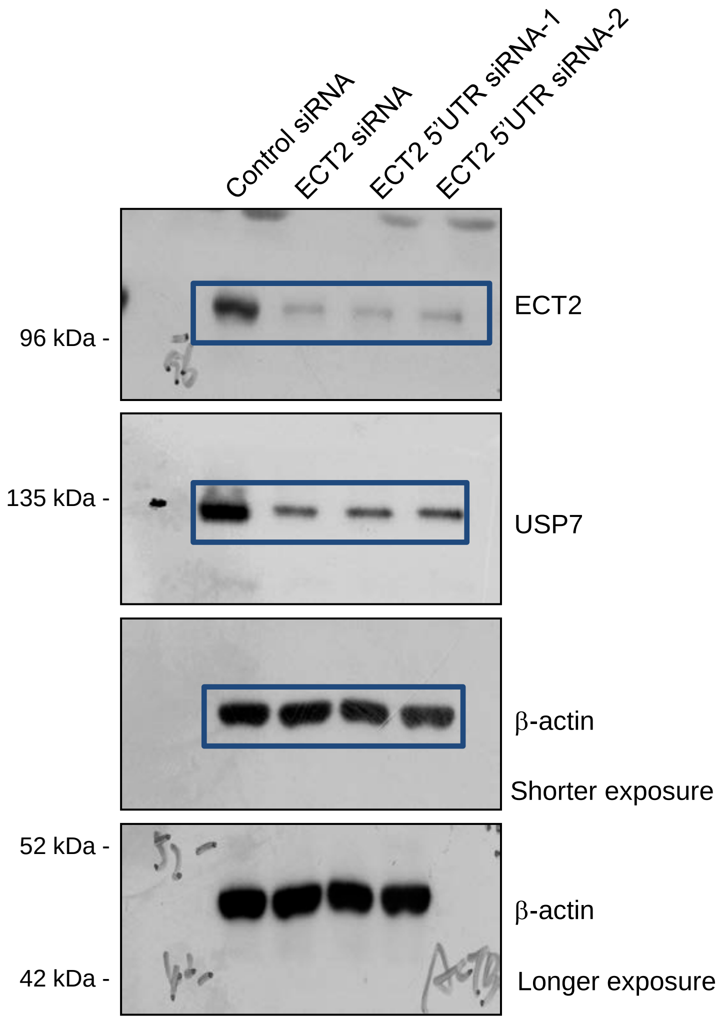
Control siRNA
ECT2 siRNA
ECT2 5'UTR siRNA-1
ECT2 5'UTR siRNA-2
96 kDa -
ECT2
135 kDa -
USP7
β-actin
Shorter exposure
52 kDa -
42 kDa -
β-actin
Longer exposure

Left panel

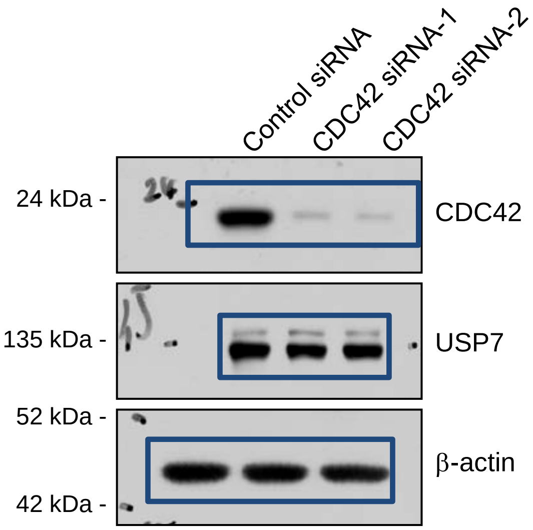

middle panel

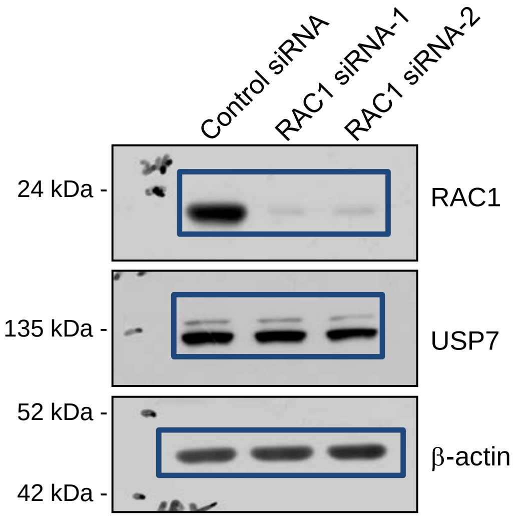

right panel

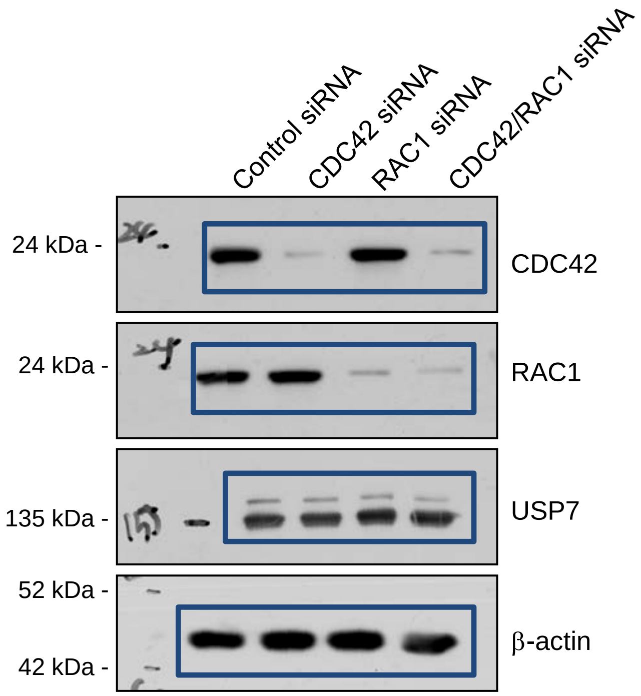

Full unedited gel for Figure S2D

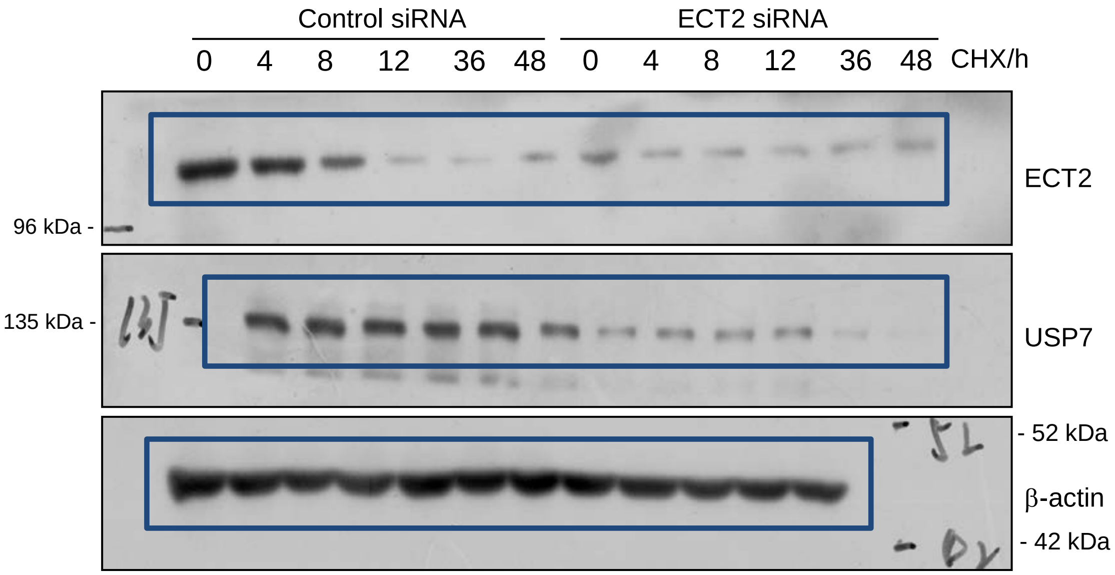

Full unedited gel for Figure S3A

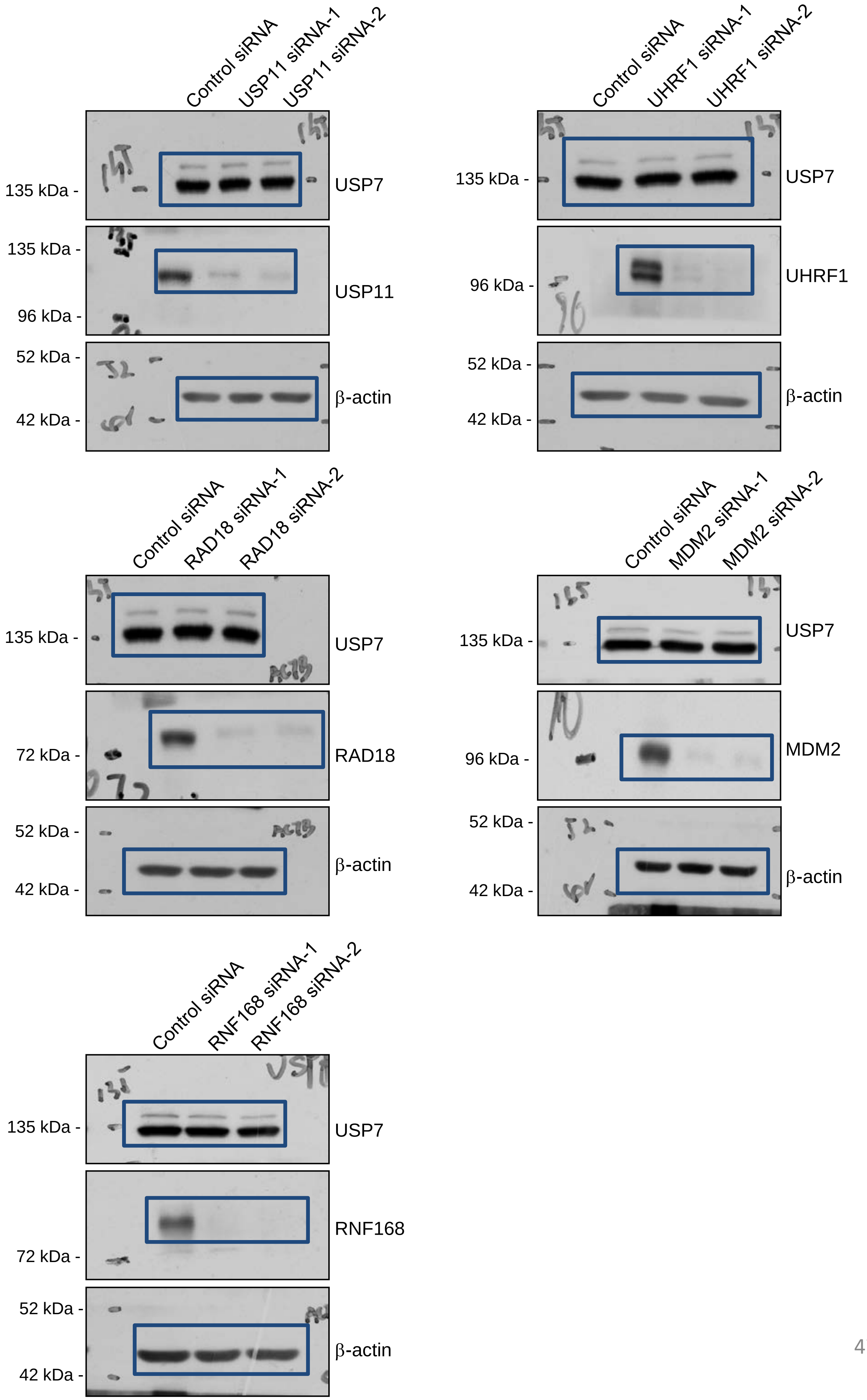

Full unedited gel for Figure S3B

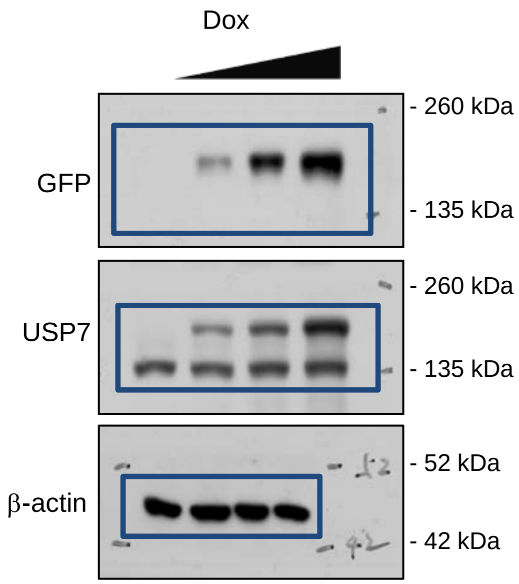

Full unedited gel for Figure S3C

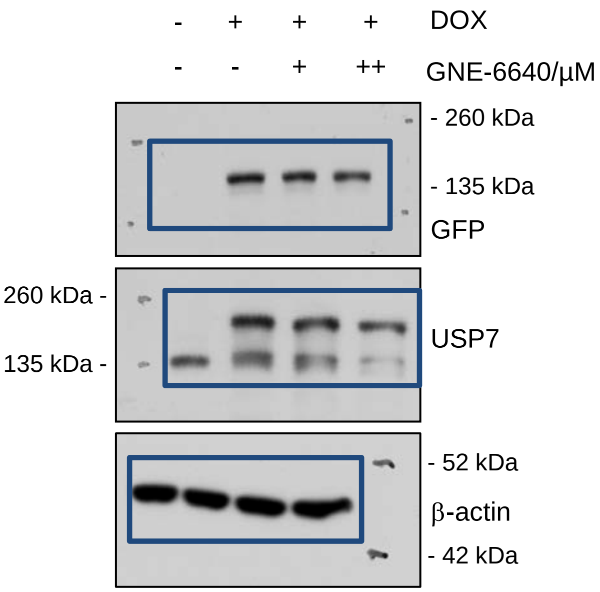

Full unedited gel for Figure S3D

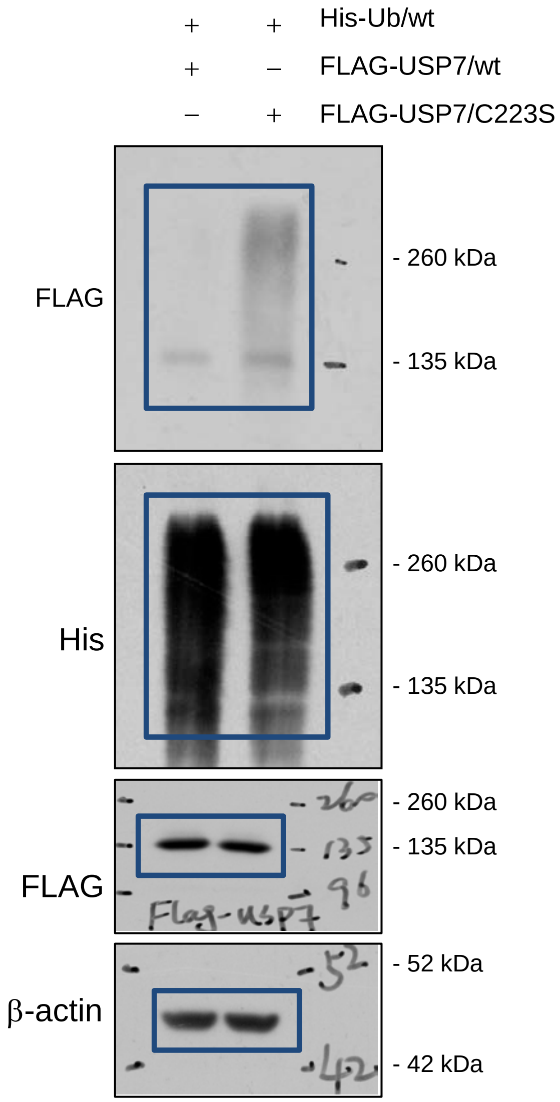

Full unedited gel for Figure S3I

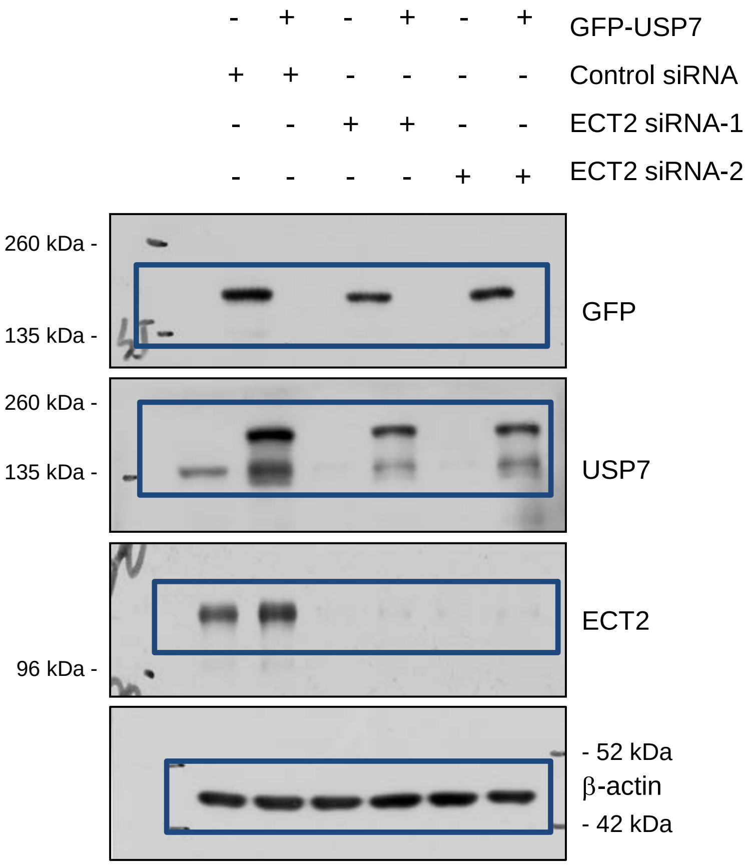

Full unedited gel for Figure S5A

Left panel

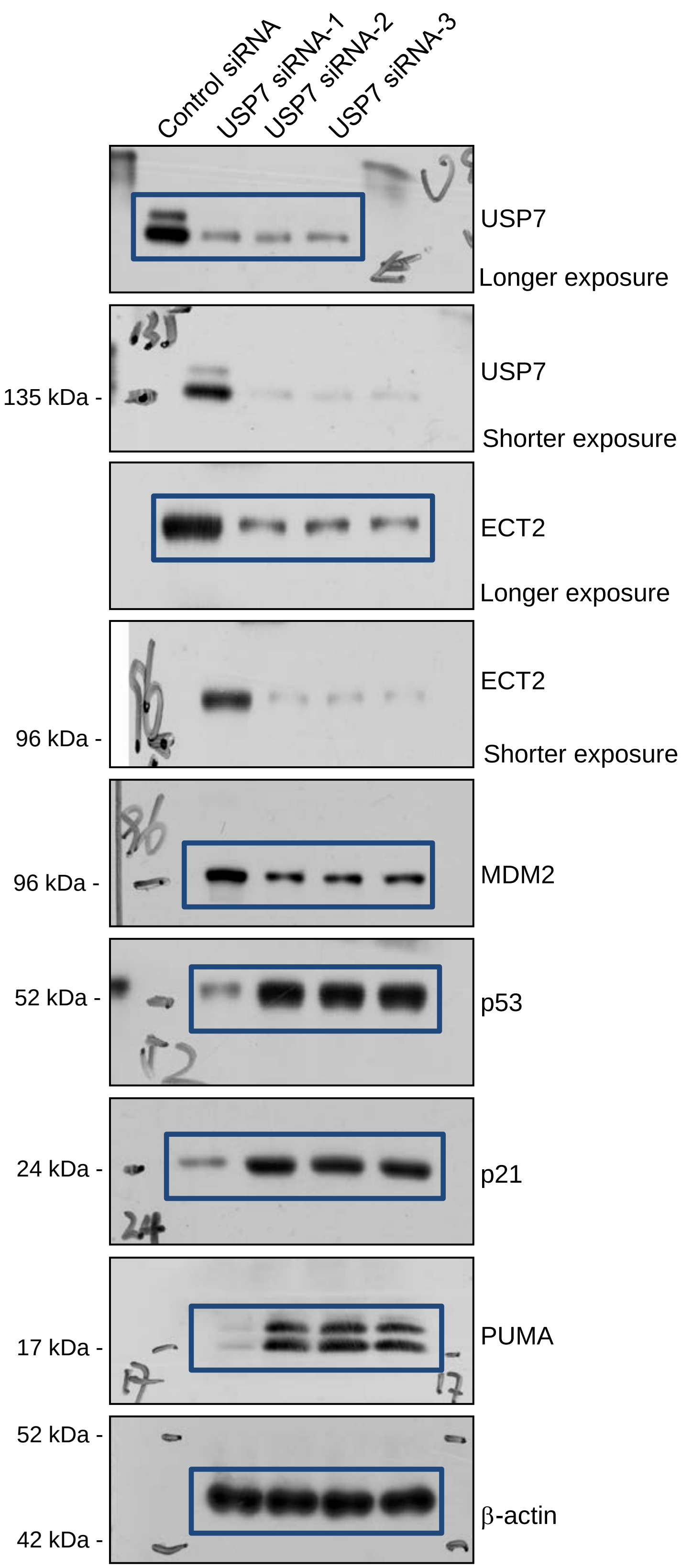

Right panel

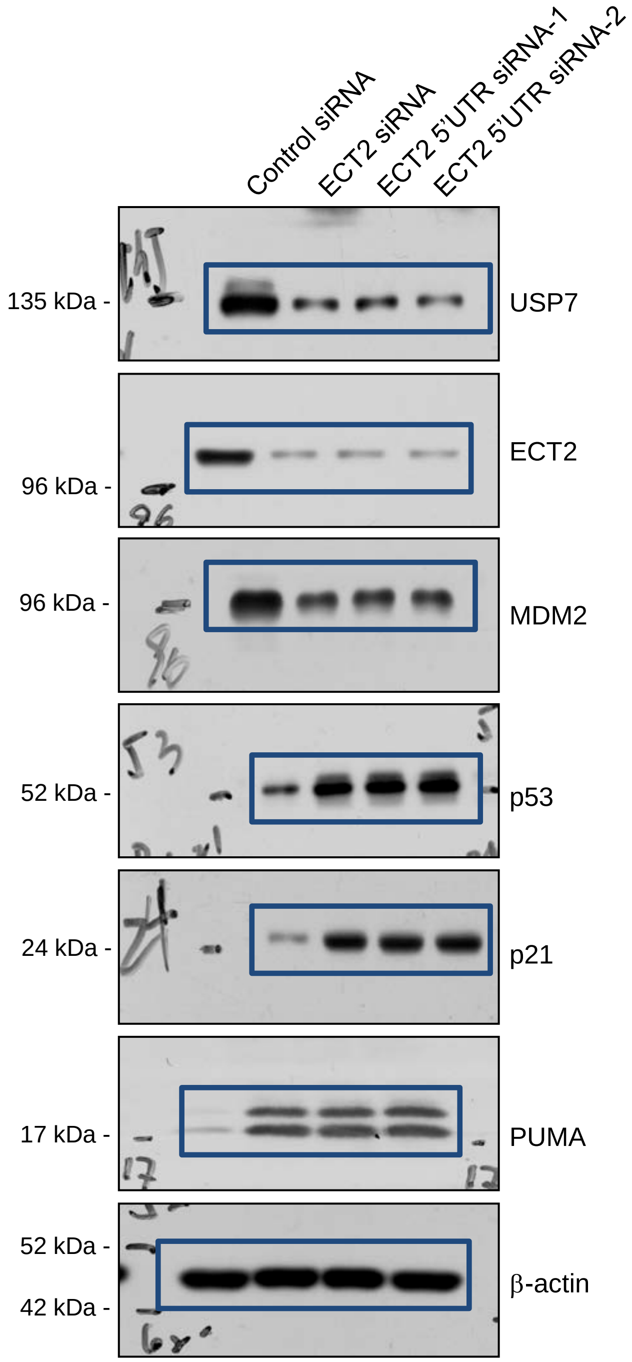

Full unedited gel for Figure S5B

Left panel

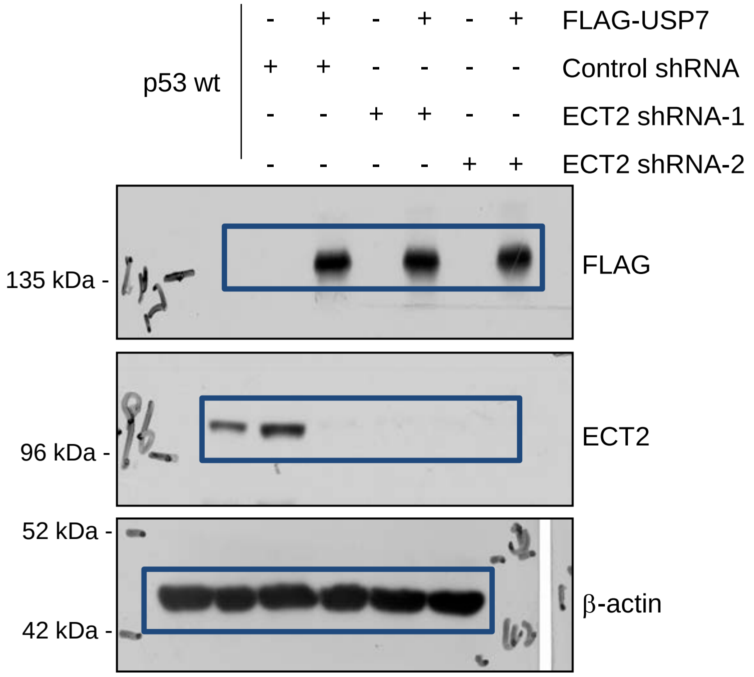

right panel

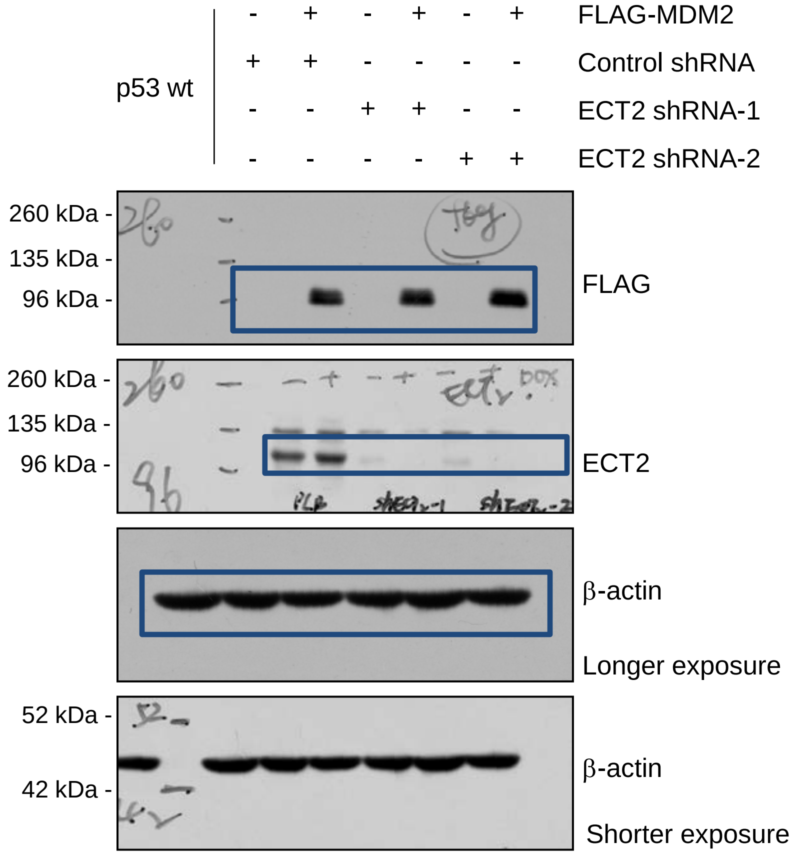

Left panel

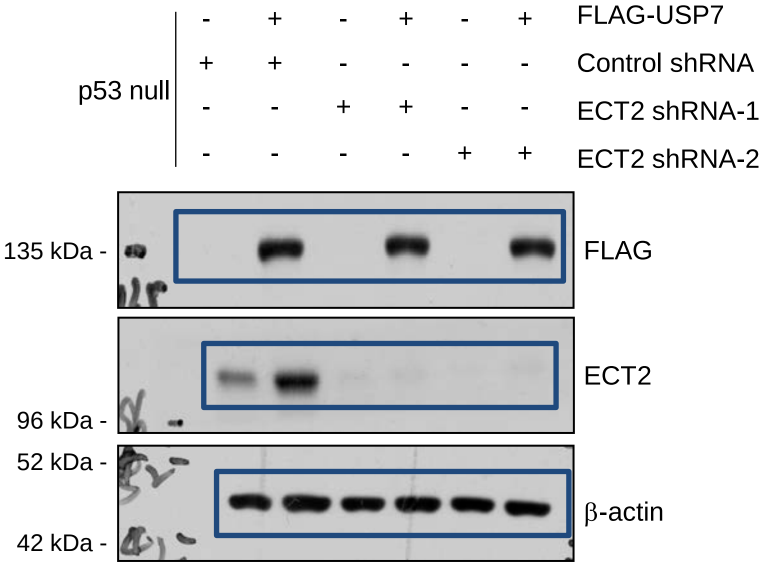

right panel

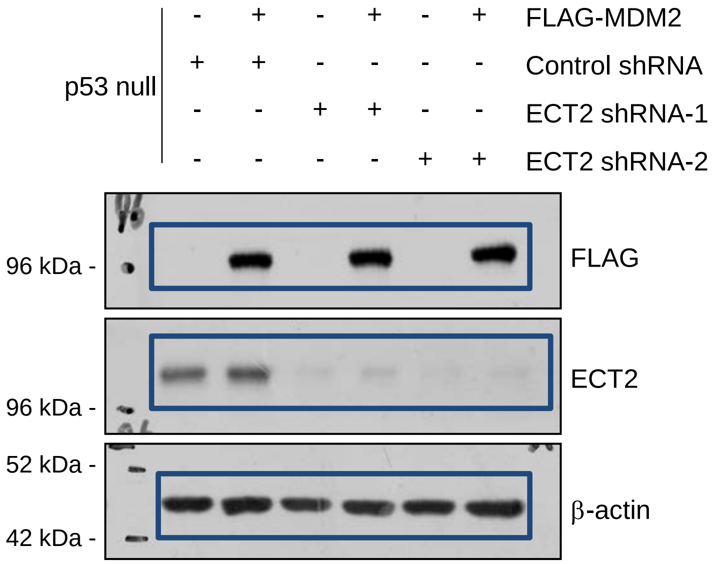

upper panel

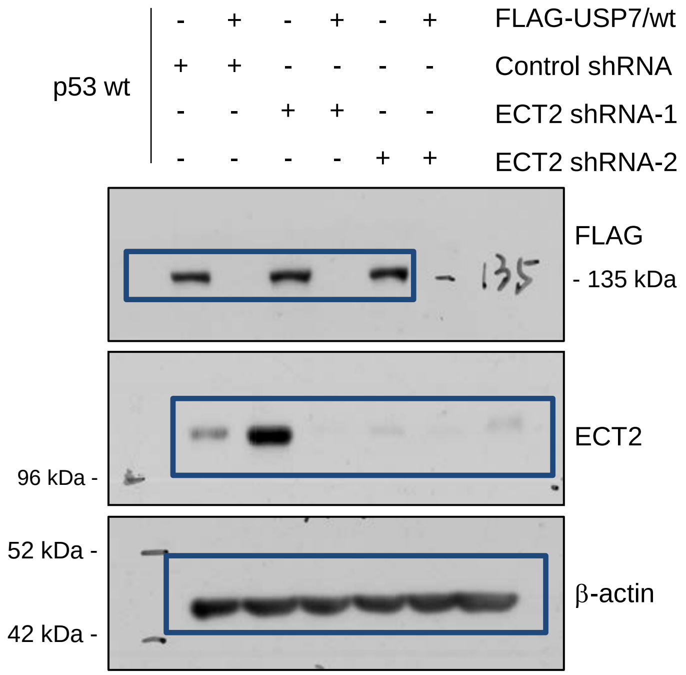

lower panel

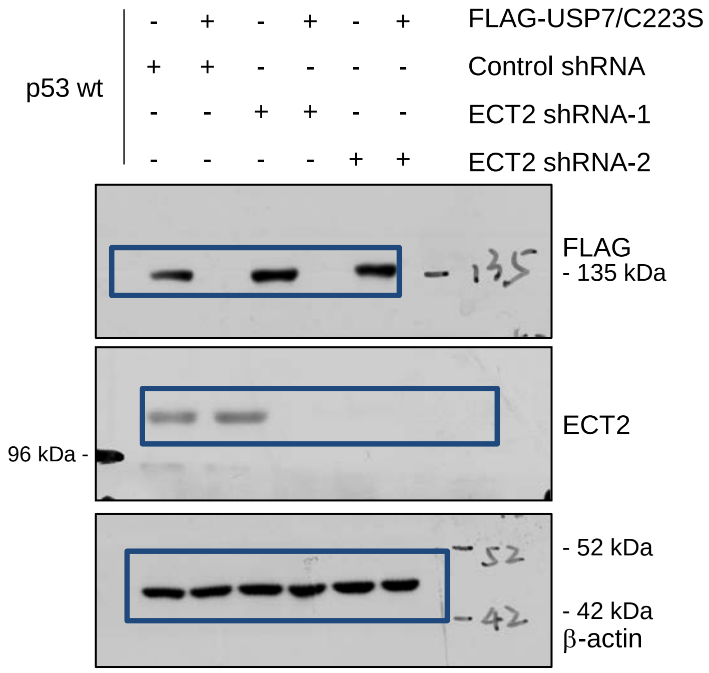

upper panel

p53 null

FLAG-USP7/wt	-	+	-	+	-	+
Control shRNA	+	+	-	-	-	-
ECT2 shRNA-1	-	-	+	+	-	-
ECT2 shRNA-2	-	-	-	-	+	+

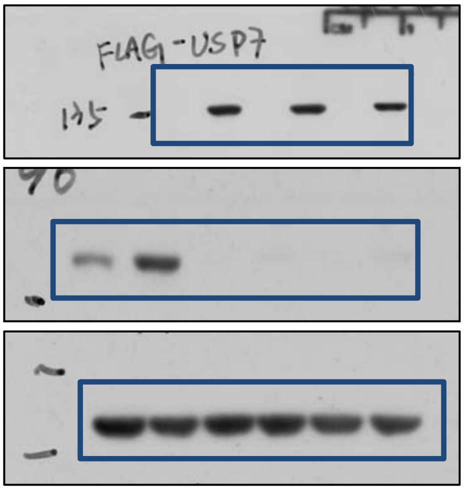

lower panel

p53 null

FLAG-USP7/C223S	-	+	-	+	-	+
Control shRNA	+	+	-	-	-	-
ECT2 shRNA-1	-	-	+	+	-	-
ECT2 shRNA-2	-	-	-	-	+	+

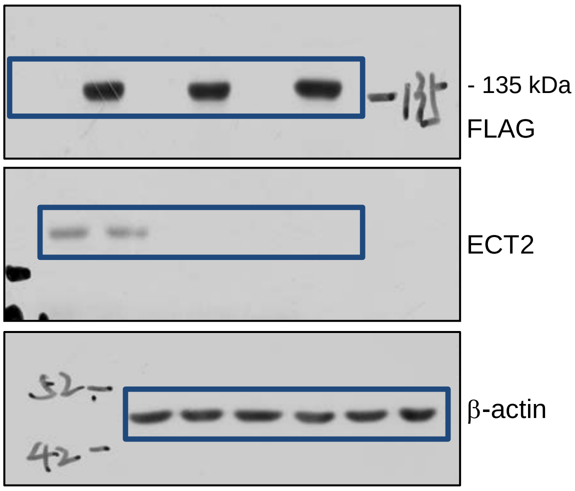

Supplementary File 1

Mass Spectrometry Analysis of ECT2-containing Protein Complex

Description	Score	Coverage	# Proteins	# Unique Peptides	# Peptides	# PSMs	# AAs	MW [kDa]	calc. pI				
E3 ubiquitin-protein ligase TRIP12 OS=Homo sapiens GN=TRIP12	317.9541893	27.9	10	1	47	115	2025	223.7604282	8.396972656				
	Sequence	# PSMs	# Proteins	Modifications	ΔCn	q-Value	PEP	XCorr	Charge	MH+ [Da]	ΔM [ppm]	RT [min]	# Missed Cleavages
	KPNPLANSNTSGYSESK	6	4		0.0000	0	0.001063	5.26	3	1793.86448	-1.12	12.84	0
	IIEAAHQVGEDEISLSTLGR	2	2		0.0000	0	5.39E-09	5.23	2	2138.11284	1.85	28.08	0
	HLAESESLLTSPPK	4	4		0.0000	0	0.000004566	4.81	2	1508.79387	-1.12	19.76	0
	LVDNFQHEENLLQQVASK	2	4		0.0000	0	0.000001033	4.58	3	2112.07322	0.53	30.08	0
	NTAGAQPQDDSIGGR	2	9		0.0000	0	0.000005535	4.36	2	1486.68498	-2.13	12.40	0
	GLPPHLFGPLGPR	5	4		0.0000	0	0.0000353	4.36	3	1357.77326	-0.54	27.41	0
	NHAVSSHIASMLSSQDLK	1	4		0.0000	0	0.0001226	4.29	3	1924.95224	-1.23	22.67	0
	LLDTNPEINQSDSQDSR	3	4		0.0000	0	0.000000616	4.23	2	1931.89116	-1.56	20.73	0
	QAESVMQDLGSSR	1	4		0.0000	0	0.00004503	4.11	2	1407.64763	-4.06	21.78	0
	QETSLTSHDLFDIDPVVAR	1	5		0.0000	0	0.00008031	3.99	3	2143.06583	-0.40	30.84	0
	SIVSESDVSSFEIQHSGFVK	2	4		0.0000	0	0.000001198	3.96	2	2182.05864	-3.53	29.44	0
	QAESVmQDLGSSR	2	4	M6(Oxidation)	0.0000	0	0.000261	3.73	2	1423.64470	-2.50	15.09	0
	SSAVVVDAIPVFLEK	2	4		0.0000	0	0.001619	3.67	2	1573.87798	-3.62	33.09	0
	mADPESNQEAVNSSAAR	2	6	M1(Oxidation)	0.0000	0	0.000005125	3.55	2	1792.77410	-1.47	12.64	0
	AQLmKEDPELAK	2	4	M4(Oxidation)	0.0000	0	0.002028	3.50	2	1388.70525	-2.76	11.78	1
	VEPVGNAPLLALVHK	4	4		0.0000	0	0.00003521	3.42	2	1556.91265	-2.14	28.48	0
	IDPLALVQAIER	5	4		0.0000	0	0.0000567	3.25	2	1337.77617	-1.97	36.51	0
	LSTQSNSNNIEPAR	3	4		0.0000	0	0.00007243	3.19	2	1530.74846	-1.48	13.07	0
	KPNPLANSNTSGYSESKK	1	4		0.0000	0	0.01366	3.11	3	1921.95407	-3.84	10.65	1
	EGIFAVDTmLK	2	4	M9(Oxidation)	0.0000	0	0.00648	2.97	2	1239.62798	-0.85	27.91	0
	DDSLDLSPQGR	1	4		0.0000	0	0.00333	2.97	2	1202.56499	0.13	21.42	0
	lQALLEAR	1	4	N-Term(Acetyl)	0.0000	0	0.01071	2.95	2	955.55419	-3.11	27.90	0
	LVDLPLGLPFYK	2	5		0.0000	0	0.0003355	2.92	2	1374.79900	-3.06	35.05	0
	LFLQFVTGSPR	2	5		0.0000	0	0.0003791	2.91	2	1264.70305	-1.46	31.40	0
	LPDYSSIEIMR	1	4		0.0000	0	0.0001731	2.85	2	1323.65862	-2.08	29.92	0
	SVESTcLcFAR	1	4	C6(Carbamidomethyl); (	0.0000	0	0.0005841	2.79	2	1329.59111	-1.15	22.66	0
	TcPFFFPFDTR	4	4	C2(Carbamidomethyl)	0.0000	0	0.004412	2.79	2	1434.64543	-3.98	34.98	0
	EIIPTSEFINSK	3	4		0.0000	0	0.0004296	2.73	2	1377.72368	-1.74	27.50	0
	LQALLEAR	3	4		0.0000	0	0.006551	2.72	2	913.54570	-0.97	20.00	0
	EGIFAVDTMLK	1	4		0.0000	0	0.007084	2.69	2	1223.63188	-1.82	31.40	0
	ALTYmmEALPR	5	4	M5(Oxidation); M6(Oxid	0.0000	0	0.002284	2.68	2	1327.63542	-2.35	25.60	0
Bloom syndrome protein OS=Homo sapiens GN=BLM	221.1557527	33.31	6	38	38	74	1417	158.900716	7.488769531				
	Sequence	# PSMs	# Proteins	Modifications	ΔCn	q-Value	PEP	XCorr	Charge	MH+ [Da]	ΔM [ppm]	RT [min]	# Missed Cleavages
	DGLAALAYHAGLSDSARDEVQQK	1	2		0.0000	0	0.00001998	6.74	3	2415.18802	-0.82	26.34	1
	DGEISHcLLFYTYHDVTR	1	2	C7(Carbamidomethyl)	0.0000	0	1.744E-09	6.29	3	2226.02579	-1.21	30.00	0
	SSSIIGSSSASHTSQATSGANSK	3	2		0.0000	0	0.00001953	5.87	3	2152.00504	-2.92	11.17	0
	DGLAALAYHAGLSDSAR	2	2		0.0000	0	0.000004331	5.47	2	1687.82939	-6.23	27.93	0
	QKFPSVPVMALTATANPR	3	2		0.0000	0	0.0003488	5.38	3	1928.03299	-4.84	28.26	1
	ELNFSHLPSNSVSPGDcLLTTTLGK	2	2	C17(Carbamidomethyl)	0.0000	0	0.000005394	5.23	3	2687.32614	-3.15	31.96	0
	SAAEELDEEIPVSSHYFASK	3	2		0.0000	0	0.00002306	4.68	3	2209.02390	-2.59	29.07	0
	YRPDSLDGPMEGDScPTGNSMK	1	2	C15(Carbamidomethyl)	0.0000	0	0.00008242	4.64	3	2413.99948	-2.63	22.09	0
	TSSDNNVSVTNVSVAK	1	3		0.0000	0	0.0001626	4.09	2	1621.79802	-2.97	17.75	0
	YGAEVISVLQK	2	2		0.0000	0	0.0002898	3.78	2	1206.67351	0.50	25.57	0
	NLFERPLFNTHLQK	1	2		0.0000	0	0.000553	3.65	2	1756.94731	-1.18	24.54	0
	YRPDSLDGPmEGDScPTGNSMK	1	2	M10(Oxidation); C15(Ca	0.0000	0	0.00008263	3.60	3	2429.99924	-0.63	19.03	0
	DLDTSDRKEDVLSTSK	3	3		0.0000	0	0.0002317	3.51	2	1808.88469	-1.45	16.03	2
	REcDTMADTLQR	2	2	C3(Carbamidomethyl)	0.0000	0	0.0001601	3.38	2	1495.66155	-0.88	15.88	1
	LLDcGNELLQQR	2	2	C4(Carbamidomethyl)	0.0000	0	0.0002694	3.37	2	1458.73406	-2.02	24.91	0
	SLLPDFLQTPK	2	3		0.0000	0	0.003379	3.27	2	1258.70342	-0.64	32.39	0
	mSmQELNPETSTDcDAR	1	2	M1(Oxidation); M3(Oxic	0.0000	0	0.00003308	2.94	2	2016.78972	-2.34	17.35	0
	LIDTIPDDKLK	3	2		0.0000	0	0.001392	2.92	2	1270.72319	-1.71	20.37	1
	SVEGYYQESGR	2	2		0.0000	0	0.00142	2.91	2	1274.56255	-1.77	17.28	0
	HTASINDLER	4	2		0.0000	0	0.01173	2.88	2	1155.57659	1.09	14.42	0
	LISTLENLYER	3	2		0.0000	0	0.0002124	2.75	2	1350.72551	-0.66	29.03	0
	EVVcTTQNTPTVK	3	3	C4(Carbamidomethyl)	0.0000	0	0.0001385	2.61	2	1476.73186	-3.02	15.33	0
	AQLYTTNTVK	2	3		0.0000	0	0.003047	2.60	2	1138.60869	-1.41	14.38	0
	NLEEAELHSTEK	3	3		0.0000	0	0.003942	2.58	2	1399.66985	-0.12	14.31	0
	LLTEVDFNK	2	2		0.0000	0	0.004363	2.54	2	1078.57781	-0.13	23.75	0
Ubiquitin carboxyl-terminal hydrolase 7 OS=Homo sapiens GN=USP7	244.1806576	33.15	9	36	36	103	1086	126.1885602	5.706542969				
	Sequence	# PSMs	# Proteins	Modifications	ΔCn	q-Value	PEP	XCorr	Charge	MH+ [Da]	ΔM [ppm]	RT [min]	# Missed Cleavages
	LSEVLQAVTDHDIPQQLVER	3	4		0.0000	0	6.737E-09	5.65	3	2290.20096	-1.26	30.24	0
	HQYINEDEYEVNLK	1	3		0.0000	0	5.012E-08	5.03	2	1793.83000	-2.29	23.45	0
	FDKDHDVMLFLK	1	4		0.0000	0	0.0000395	4.59	3	1507.75614	-3.52	26.77	1
	IQSLLDIQEKEFEK	2	3		0.0000	0	0.0000627	4.25	3	1719.91349	-1.71	27.61	1
	VFYELQHSDKPVGTK	2	5		0.0000	0	0.0001171	4.00	3	1747.89786	-2.04	16.73	0
	LNTDPmLLQFFK	3	4	M6(Oxidation)	0.0000	0	0.0002151	3.47	2	1482.76384	-1.58	33.60	0
	IIGVHQEDELLEcLSPATSR	2	4	C13(Carbamidomethyl)	0.0000	0	0.0005371	3.47	3	2267.12473	-3.97	30.21	0
	RPAMLDNEADGNK	3	4		0.0000	0	0.0002683	3.46	2	1430.66728	-1.44	11.72	0
	LNTDPMLLQFFK	5	4		0.0000	0	0.0002535	3.43	2	1466.76848	-1.90	36.04	0
	TIPNDPGFVVTLSNR	5	4		0.0000	0	0.00004238	3.18	2	1629.86040	0.51	28.86	0
	KLYYQQLK	3	4		0.0000	0	0.00488	2.84	2	1083.61833	-1.28	14.91	1
	FDDDVVSR	3	5		0.0000	0	0.01051	2.79	2	952.43511	-2.09	16.85	0
	DLLQFFKPR	7	4		0.0000	0	0.005438	2.79	2	1163.65361	-3.09	31.25	0
	LSESVLSPPcFVR	4	7	C10(Carbamidomethyl)	0.0000	0	0.004489	2.78	2	1490.76421	-2.04	28.89	0
	MNYFQVAK	4	4		0.0000	0	0.007779	2.78	2	1000.49199	-0.11	22.23	0
	IQDYDVSLDK	3	4		0.0000	0	0.00254	2.73	2	1195.58391	-0.20	21.55	0
	VDVIFcDK	5	4	C6(Carbamidomethyl)	0.0000	0	0.008613	2.72	2	995.48442	-2.28	22.72	0
	FMYDPQTDQNIK	1	5		0.0000	0	0.0005958	2.68	2	1499.67912	-2.96	23.28	0
	ISHLFFHK	2	9		0.0000	0	0.01534	2.65	2	1028.56890	1.22	16.03	0
	IINYRDDEK	2	9		0.0000	0	0.008896	2.54	2	1165.58196	-2.46	10.81	1
	AVYMMPTEGDDSSK	1	5		0.0000	0	0.0004966	2.52	2	1530.63994	-3.40	22.37	0
	VLLDNVENK	2	5		0.0000	0	0.004559	2.49	2	1043.57195	-1.20	17.77	0
	DDPENDNSELPTAK	1	4		0.0000	0	0.003594	2.48	2	1544.66533	-3.77	17.32	0
	LLEIVSYK	4	4		0.0000	0	0.01301	2.48	2	964.56993	-1.51	25.14	0
	IQSLLDIQEK	2	3		0.0000	0	0.002718	2.41	2	1186.66582	-1.70	25.58	0
NAD-dependent protein deacetylase sirtuin-1 OS=Homo sapiens GN=SIRT1	33.09188819	10.71	4	7	7	14	747	81.62996464	4.665527344				
	Sequence	# PSMs	# Proteins	Modifications	ΔCn	q-Value	PEP	XCorr	Charge	MH+ [Da]	ΔM [ppm]	RT [min]	# Missed Cleavages
	NYTQNIDTLEQVAGIQR	1	4		0.0000	0	0.000004943	4.15	2	1962.98711	-0.46	32.21	0
	LSEITEKPPR	2	3		0.0000	0	0.001248	2.85	2	1169.65141	-0.95	12.90	0
	FIALSDKEGK	3	4		0.0000	0	0.007814	2.54	2	1107.60466	0.14	14.16	1
Protein ECT2 OS=Homo sapiens GN=ECT2	7712.724262	68.29	19	79	79	2486	883	99.98727694	7.488769531				
	Sequence	# PSMs	# Proteins	Modifications	ΔCn	q-Value	PEP	XCorr	Charge	MH+ [Da]	ΔM [ppm]	RT [min]	# Missed Cleavages
	KRPSAEHSLSIGSLLDISNTPESSINYGDTPK	14	5		0.0000	0	8.437E-11	10.09	3	3413.72092	0.72	32.02	1
	RNEQDFYAAVDDFRNEFK	53	5		0.0000	0	6.083E-11	8.79	3	2264.03281	-1.75	31.78	2
	rNEQDFYAAVDDFRNEFK	11	5	N-Term(Acetyl)	0.0000	0	4.647E-10	8.56	3	2306.04489	-1.06	36.40	2
	RPSAEHSLSIGSLLDISNTPESSINYGDTPK	1	5		0.0000	0	3.31E-08	7.57	4	3285.60561	-5.44	33.81	0
	VPPFQDcILSFLGFSDEEKTNMEEMTEMQGGK	4	5	C7(Carbamidomethyl)	0.0000	0	4.524E-08	7.29	4	3694.63759	-1.24	44.10	1
	cTHLVVEENIVKDLPFEPSK	2	5	C1(Carbamidomethyl)	0.0000	0	5.464E-07	7.17	3	2354.19980	-2.70	32.02	1
	cTHLVVEENIVKDLPFEPSKK	4	5	C1(Carbamidomethyl)	0.0000	0	5.411E-10	6.92	3	2482.29544	-2.29	30.37	2
	VPPFQDcILSFLGFSDEEKTNMEEMTEmQGGK	6	5	C7(Carbamidomethyl); M	0.0000	0	4.006E-08	6.87	4	3710.62758	-2.56	43.44	1
	VTEISKENLLIGSTSYVEEEMPQIETR	92	8		0.0000	0	0.000000337	6.48	3	3095.54074	-1.51	38.29	1
	LPSVALLLNDLKK	55	5		0.0000	0	0.000002561	6.30	3	1423.88697	-0.97	32.85	1
	NEQDFYAAVDDFRNEFK	20	5		0.0000	0	1.536E-07	6.16	3	2107.92990	-2.73	37.30	1
	vTEISKENLLIGSTSYVEEEMPQIETR	1	8	N-Term(Acetyl)	0.0000	0	0.000000165	6.14	3	3137.56198	1.91	37.89	1
	VTHLVANcTQGEKFR	10	6	C8(Carbamidomethyl)	0.0000	0	7.705E-08	5.96	3	1759.88907	-1.03	17.31	1
	IKDDLEDLIVNWDESK	10	6		0.0000	0	2.967E-08	5.93	2	1931.95500	-2.46	37.38	1
	QIFDVVYEVDGcPANLLSSHR	11	5	C12(Carbamidomethyl)	0.0000	0	4.557E-10	5.87	3	2419.16141	-4.03	33.47	0
	AIGSLKEVMTHINEDKR	3	5		0.0000	0	0.000004037	5.64	3	1941.01737	-2.55	25.84	2
	ENLLIGSTSYVEEEMPQIETR	32	8		0.0000	0	2.818E-08	5.63	2	2438.16997	-2.33	37.66	0
	VPPFQDcILSFLGFSDEEK	7	5	C7(Carbamidomethyl)	0.0000	0	0.000004614	5.57	3	2228.05570	-1.08	45.25	0
	ADAENLIYTADPESFEVNTKDmDSTLSR	3	6	M22(Oxidation)	0.0000	0	2.759E-08	5.57	3	3148.41318	-4.21	30.96	1
	VTEISKENLLIGSTSYVEEEmPQIETR	8	8	M21(Oxidation)	0.0000	0	2.308E-07	5.54	3	3111.54416	1.23	29.91	1
	eNLLIGSTSYVEEEMPQIETR	8	8	N-Term(Acetyl)	0.0000	0	3.82E-08	5.49	2	2480.17949	-2.71	39.59	0
	KSVSMLSLNTPNSNRK	5	6		0.0000	0	0.000009228	5.49	3	1775.94156	-0.99	18.61	2
	ENLLIGSTSYVEEEmPQIETR	23	8	M15(Oxidation)	0.0000	0	3.276E-08	5.45	2	2454.16191	-3.53	30.64	0
	KSVSMLSLNTPNSNR	15	6		0.0000	0	2.098E-07	5.37	2	1647.84807	-0.17	19.02	1
	HTADENPDKSTLEK	9	5		0.0000	0	0.000115	5.27	3	1584.74942	-0.41	11.05	1
	KSVSmLSLNTPNSNRK	2	6	M5(Oxidation)	0.0000	0	0.00003802	5.21	3	1791.93180	-3.59	11.92	2
	VAVSLGTPIMKPEWIYK	36	5		0.0000	0	3.751E-07	5.20	3	1932.06241	-2.07	30.73	0
	ADAENLIYTADPESFEVNTK	32	6		0.0000	0	0.00005933	5.07	3	2227.03873	-0.66	34.63	0
	cTHLVVEENIVK	28	5	C1(Carbamidomethyl)	0.0000	0	0.00001959	5.04	2	1440.75200	0.29	18.97	0
	hTADENPDKSTLEK	10	5	N-Term(Acetyl)	0.0000	0	0.0002542	4.95	3	1626.75693	-2.28	11.27	1
	aDAENLIYTADPESFEVNTK	6	6	N-Term(Acetyl)	0.0000	0	0.000005857	4.94	2	2269.04302	-3.41	33.24	0
	LPSVALLLNDLK	23	5		0.0000	0	0.00003732	4.90	2	1295.79070	-2.07	36.41	0
	rNEQDFYAAVDDFR	5	5	N-Term(Acetyl)	0.0000	0	2.196E-07	4.90	2	1787.80046	1.14	32.99	1
	RNEQDFYAAVDDFR	24	5		0.0000	0	6.955E-08	4.88	2	1745.78557	-1.31	31.07	1
	VILVQEAGKQEELIK	133	10		0.0000	0	4.198E-07	4.82	2	1696.98223	-1.31	23.43	1
	VAVSLGTPImKPEWIYK	34	5	M10(Oxidation)	0.0000	0	0.00004334	4.81	3	1948.06107	-0.13	28.72	0
	TSLADSSIFDSKVTEISK	3	10		0.0000	0	0.000003356	4.81	2	1927.99040	2.30	27.57	1
	nEQDFYAAVDDFRNEFK	1	5	N-Term(Acetyl)	0.0000	0	0.00001816	4.80	2	2149.94316	-1.42	38.94	1
	NEQDFYAAVDDFR	10	5		0.0000	0	0.0000612	4.74	2	1589.68545	-0.81	31.08	0
	vILVQEAGKQEELIK	13	10	N-Term(Acetyl)	0.0000	0	0.000003708	4.69	2	1738.99224	-1.60	31.42	1
	LSSTSSLAGIPSPSLVSLPSFFER	8	3		0.0000	0	0.0000737	4.65	2	2479.30571	-0.92	41.14	0
	VIGPPVVLNcSQK	65	7	C10(Carbamidomethyl)	0.0000	0	0.0006592	4.55	3	1410.77478	-1.87	23.24	0
	VTHLVANcTQGEK	54	6	C8(Carbamidomethyl)	0.0000	0	0.00004331	4.49	3	1456.71906	-1.57	9.57	0
	vAVSLGTPIMKPEWIYK	3	5	N-Term(Acetyl)	0.0000	0	0.000008767	4.47	3	1974.07486	-1.07	36.07	0
	DFNSKVTHLVANcTQGEK	1	6	C13(Carbamidomethyl)	0.0000	0	0.00006449	4.47	3	2047.98905	1.17	22.06	1
	lVTLVHHMGGVIR	25	6	N-Term(Acetyl)	0.0000	0	0.00005869	4.31	4	1473.83584	-0.07	28.26	0
	FHAFLKINQAKPEcGR	6	5	C14(Carbamidomethyl)	0.0000	0	0.000003246	4.27	3	1915.99436	-0.88	18.50	1
	VILVQEAGKQEELIKALK	2	10		0.0000	0	0.0001823	4.24	3	2009.20182	0.60	26.71	2
	kSVSMLSLNTPNSNR	1	6	N-Term(Acetyl)	0.0000	0	0.0001019	4.21	2	1689.85869	-0.13	26.44	1
	HIHLmPLSQIK	20	6	M5(Oxidation)	0.0000	0	0.001228	4.20	2	1332.74443	-0.96	24.06	0
	SVSMLSLNTPNSNRK	16	6		0.0000	0	0.00001032	4.20	3	1647.84720	-0.70	19.86	1
	LVTLVHHMGGVIR	104	6		0.0000	0	0.0003308	4.18	4	1431.82155	-2.67	22.64	0
	VETISLGEHPcDR	32	6	C11(Carbamidomethyl)	0.0000	0	0.0005405	4.18	3	1512.70877	-1.59	17.31	0
	tSLADSSIFDSK	22	10	N-Term(Acetyl)	0.0000	0	0.00005119	4.13	2	1312.62578	-0.74	31.66	0
	LVTLVHHMGGVIRK	31	6		0.0000	0	0.0001221	4.11	2	1559.91936	-0.63	17.21	1

	TIFGSIPDIFDVHTK	37	6		0.0000	0	0.000009481	4.09	2	1689.88298	-1.03	37.07	0
	LVTLVHHmGGVIR	106	6	M8(Oxidation)	0.0000	0	0.0006022	4.08	4	1447.81789	-1.65	15.23	0
	TSLADSSIFDSK	62	10		0.0000	0	0.0003297	4.07	2	1270.61577	-0.33	27.86	0
	RALMTSHGSVEGR	10	6		0.0000	0	0.0001842	4.07	3	1400.70600	-0.27	12.11	1
	cTHLVVEENIVKDLPFEPSKK	1	5	N-Term(Acetyl)	0.0000	0	2.856E-08	4.04	2	2467.27837	-4.81	33.23	2
	SVSMLSLNTPNSNR	20	6		0.0000	0	0.00004773	3.98	2	1519.75347	0.06	23.81	0
	vTHLVANcTQGEK	18	6	N-Term(Acetyl); C8(Carl	0.0000	0	0.00005335	3.94	2	1498.72978	-1.42	20.76	0
	HIHLmPLSQIKK	5	6	M5(Oxidation)	0.0000	0	0.001305	3.90	4	1460.83791	-1.89	16.18	1
	sVSMLSLNTPNSNR	5	6	N-Term(Acetyl)	0.0000	0	0.00003582	3.89	2	1561.76177	-1.40	30.87	0
	vETISLGEHPcDR	8	6	N-Term(Acetyl); C11(Ca	0.0000	0	0.00004534	3.82	2	1554.71782	-2.53	22.27	0
	hIHLmPLSQIK	2	6	N-Term(Acetyl); M5(Oxi	0.0000	0	0.003626	3.77	3	1374.75428	-1.45	22.37	0
	aGETMYLYEK	22	6	N-Term(Acetyl)	0.0000	0	0.0007027	3.75	2	1246.56474	-1.05	23.56	0
	EVMTHINEDKR	14	5		0.0000	0	0.0001818	3.74	2	1371.66728	-0.95	12.28	1
	TNMEEMTEMQGGK	11	5		0.0000	0	0.00003197	3.71	2	1485.59831	-2.43	20.46	0
	KSVSmLSLNTPNSNR	7	6	M5(Oxidation)	0.0000	0	0.000003351	3.71	2	1663.84099	-1.37	15.83	1
	LVTLVHHmGGVIRK	9	6	M8(Oxidation)	0.0000	0	0.002784	3.68	4	1575.91433	-0.59	12.76	1
	TNMEEMTEmQGGK	22	5	M9(Oxidation)	0.0000	0	0.0001728	3.68	2	1501.59209	-3.16	16.77	0
	ALMTSHGSVEGR	27	6		0.0000	0	0.0002534	3.57	3	1244.60538	0.10	10.41	0
	aLMTSHGSVEGR	18	6	N-Term(Acetyl)	0.0000	0	0.0001481	3.57	2	1286.61589	0.05	13.90	0
	vIGPPVVLNcSQK	12	7	N-Term(Acetyl); C10(Ca	0.0000	0	0.00127	3.56	2	1452.78459	-2.33	30.15	0
	HIHLMPLSQIKK	7	6		0.0000	0	0.0007603	3.54	4	1444.84499	-0.53	19.07	1
	QSLVELLIRPVQR	27	5		0.0000	0	0.0009907	3.53	3	1550.93625	-0.97	33.99	0
	LKETLAQLSR	40	6		0.0000	0	0.009676	3.53	3	1158.68222	-1.66	16.82	1
	TYPPFVNFFEMSK	80	6		0.0000	0	0.0001392	3.50	2	1606.75505	-3.76	39.98	0
	aLmTSHGSVEGR	17	6	N-Term(Acetyl); M3(Oxi	0.0000	0	0.0001129	3.49	3	1302.60688	-2.97	10.68	0
	SVSmLSLNTPNSNRK	11	6	M4(Oxidation)	0.0000	0	0.000203	3.49	3	1663.84165	-0.97	19.05	1
	ALmTSHGSVEGR	25	6	M3(Oxidation)	0.0000	0	0.0007889	3.45	3	1260.59809	-1.66	10.74	0
	SVSmLSLNTPNSNR	31	6	M4(Oxidation)	0.0000	0	0.000135	3.44	2	1535.74834	0.03	18.99	0
	eTDVSPFPPR	51	5	N-Term(Acetyl)	0.0000	0	0.001715	3.41	2	1186.57219	-1.47	30.55	0
	ETDVSPFPPR	82	5		0.0000	0	0.0009315	3.39	2	1144.56389	0.45	20.00	0
	AGETMYLYEK	23	6		0.0000	0	0.001105	3.37	2	1204.55205	-2.86	21.69	0
	lKETLAQLSR	10	6	N-Term(Acetyl)	0.0000	0	0.0019	3.34	2	1200.69438	-0.28	22.21	1
	lVTLVHHmGGVIR	9	6	N-Term(Acetyl); M8(Oxi	0.0000	0	0.00141	3.32	2	1489.82793	-1.96	20.97	0
	SIGDIFLKYSK	3	6		0.0000	0	0.001023	3.31	2	1270.70415	-0.05	29.21	1
	tYPPFVNFFEMSK	8	6	N-Term(Acetyl)	0.0000	0	0.0001373	3.29	2	1648.77629	2.81	42.90	0
	sVSmLSLNTPNSNR	6	6	N-Term(Acetyl); M4(Oxi	0.0000	0	0.002336	3.27	2	1577.75920	0.22	22.34	0
	tIFGSIPDIFDVHTK	2	6	N-Term(Acetyl)	0.0000	0	0.0001788	3.26	2	1731.89958	2.48	39.23	0
	HIHLMPLSQIK	40	6		0.0000	0	0.001444	3.22	2	1316.74773	-2.33	22.09	0
	qSLVELLIRPVQR	10	5	N-Term(Acetyl)	0.0000	0	0.001244	3.16	3	1592.94669	-1.03	32.48	0
	ETDVSPFPPRK	7	5		0.0000	0	0.001184	3.14	2	1272.65740	-0.74	14.79	1
	cTHLVVEENIVK	2	5	N-Term(Acetyl)	0.0000	0	0.001507	3.13	2	1425.73296	-5.42	24.73	0
	TNmEEmTEmQGGK	4	5	M3(Oxidation); M6(Oxic	0.0000	0	0.000619	3.04	2	1533.58269	-2.59	13.75	0
	iNQAKPEcGR	5	5	N-Term(Acetyl); C8(Carl	0.0000	0	0.005086	3.03	3	1214.59220	-2.05	12.89	0
	EVMTHINEDK	9	5		0.0000	0	0.0008406	3.03	2	1215.56548	-1.64	10.35	0
	EVMTHINEDKRK	4	5		0.0000	0	0.0009712	3.02	4	1499.76162	-1.30	10.57	2
	aGETmYLYEK	11	6	N-Term(Acetyl); M5(Oxi	0.0000	0	0.002713	3.02	2	1262.55730	-2.91	20.45	0
	DLVKTYPPFVNFFEmSK	1	6	M15(Oxidation)	0.0000	0	0.001084	3.01	3	2078.03391	1.68	34.39	1
	AIGSLKEVMTHINEDK	1	5		0.0000	0	0.01388	3.00	3	1784.91776	-1.93	24.07	1
	gGPILAPEEIK	8	6	N-Term(Acetyl)	0.0000	0	0.001707	2.99	2	1165.64433	-1.76	23.82	0
	EVmTHINEDK	6	5	M3(Oxidation)	0.0000	0	0.0002602	2.98	2	1231.56206	-0.27	10.29	0
	GGPILAPEEIK	30	6		0.0000	0	0.007524	2.97	2	1123.63432	-1.33	22.08	0
	QEWFWGSIQMDAR	1	6		0.0000	0	0.00004909	2.93	2	1653.75163	2.25	44.53	0
	QEWFWGSIQmDAR	3	6	M10(Oxidation)	0.0000	0	0.01817	2.93	2	1669.74187	-0.58	32.53	0
	eNLLIGSTSYVEEEmPQIETR	4	8	N-Term(Acetyl); M15(O:	0.0000	0	0.00005955	2.90	2	2496.17168	-3.79	33.13	0
	TYPPFVNFFEmSK	51	6	M11(Oxidation)	0.0000	0	0.0006005	2.89	2	1622.75005	-3.67	36.60	0
	ALKDIKVGFVK	1	6		0.0000	0	0.002802	2.89	2	1217.75920	-2.04	19.49	2
	AGETmYLYEK	19	6	M5(Oxidation)	0.0000	0	0.01303	2.74	2	1220.54766	-2.25	18.15	0
	eVMTHINEDKRK	2	5	N-Term(Acetyl)	0.0000	0	0.009522	2.63	3	1541.77073	-2.21	10.16	2
	ETLAQLSR	15	6		0.0000	0	0.01822	2.55	2	917.50316	-2.13	16.54	0
	dMDSTLSR	5	6	N-Term(Acetyl)	0.0000	0	0.01702	2.53	2	966.41802	-1.78	17.93	0
	EVmTHINEDKR	5	5	M3(Oxidation)	0.0000	0	0.02088	2.43	2	1387.66203	-1.06	12.24	1
	eTLAQLSR	7	6	N-Term(Acetyl)	0.0000	0	0.02042	2.40	2	959.51384	-1.91	21.75	0
	hVANTIcK	4	6	N-Term(Acetyl); C7(Carl	0.0000	0	0.005384	2.32	2	984.48906	-4.16	10.85	0
	TNMEEmTEmQGGK	5	5	M6(Oxidation); M9(Oxic	0.0000	0	0.0003859	2.28	2	1517.59416	1.59	10.57	0
	vILVQEAGK	4	10	N-Term(Acetyl)	0.0000	0	0.005976	2.23	2	998.58476	-3.37	25.03	0
	VILVQEAGK	28	10		0.0000	0	0.009837	2.21	2	956.57622	-1.40	16.05	0
	SPHGQTRPPASLK	7	6		0.0000	0	0.004593	2.09	2	1375.74272	-1.02	7.76	0
	AFSFSKTPK	4	6		0.0000	0	0.01422	2.04	2	1012.54503	-1.21	13.49	1
	eVmTHINEDKR	6	5	N-Term(Acetyl); M3(Oxi	0.0000	0	0.004246	2.01	3	1429.67118	-2.02	10.86	1
	DLPFEPSKK	34	5		0.0000	0	0.01313	2.00	3	1060.56543	-1.85	17.51	1
	tYPPFVNFFEmSK	2	6	N-Term(Acetyl); M11(O:	0.0000	0	0.002092	1.99	2	1664.76103	-3.33	34.93	0
	sIGDIFLK	5	6	N-Term(Acetyl)	0.0000	0	0.008099	1.95	2	934.52410	-0.39	30.11	0
	YLPLGDER	25	5		0.0000	0	0.02069	1.94	2	962.49260	-1.67	24.99	0
	qEWFWGSIQMDAR	2	6	N-Term(Acetyl)	0.0000	0	0.004424	1.82	2	1695.75432	-2.46	37.61	0
Nucleolin OS=Homo sapiens GN=NCL	200.4449061	25.21	2	17	17	65	710	76.56836278	4.703613281				
	Sequence	# PSMs	# Proteins	Modifications	ΔCn	q-Value	PEP	XCorr	Charge	MH+ [Da]	ΔM [ppm]	RT [min]	# Missed Cleavages
	GLSEDTTEETLKESFDGSVR	4	2		0.0000	0	0.000009844	5.92	3	2200.02298	-1.04	31.27	1
	TLVLSNLSYSATEETLQEVFEK	3	2		0.0000	0	0.00001404	5.81	3	2501.26810	0.91	41.49	0
	VTQDELKEVFEDAAEIR	5	2		0.0000	0	0.00000017	5.50	2	1991.99236	0.11	36.52	1
	VEGTEPTTAFNLFVGNLNFNK	2	1		0.0000	0	0.000002352	5.05	2	2312.15386	-0.86	40.17	0
	KFGYVDFESAEDLEK	1	1		0.0000	0	4.036E-08	4.20	2	1776.82573	-3.95	28.72	1
	GFGFVDFNSEEDAK	6	2		0.0000	0	0.00005361	3.99	2	1561.67668	-2.52	30.67	0
	TEADAEKTFEEK	6	2		0.0000	0	0.0002404	3.86	2	1397.64055	-1.86	13.63	1
	FGYVDFESAEDLEK	2	1		0.0000	0	0.0002411	3.45	2	1648.73491	-1.74	34.55	0
	NDLAVVDVR	6	1		0.0000	0	0.002296	3.32	2	1000.54070	-1.54	25.18	0
	TGISDVFAK	5	1		0.0000	0	0.004657	3.00	2	937.49816	-0.87	23.85	0
	GLSEDTTEETLK	1	2		0.0000	0	0.001547	2.73	2	1322.62883	-2.58	17.88	0
	EVFEDAAEIR	3	2		0.0000	0	0.003873	2.27	2	1178.56743	-1.21	23.20	0
Tubulin alpha-1A chain OS=Homo sapiens GN=TUBA1A	200.0255716	56.01	21	1	18	54	416	46.26784264	5.084472656				
	Sequence	# PSMs	# Proteins	Modifications	ΔCn	q-Value	PEP	XCorr	Charge	MH+ [Da]	ΔM [ppm]	RT [min]	# Missed Cleavages
	QLFHPEQLITGKEDAANNYAR	4	23		0.0000	0	1.626E-09	7.24	3	2415.19974	-2.28	29.09	1
	AYHEQLSVAEITNAcFEPANQMVK	1	9	C15(Carbamidomethyl)	0.0000	0	0.000005865	6.78	3	2750.27634	-5.47	32.54	0
	FDGALNVDLTEFQTNLVPYPR	1	15		0.0000	0	4.302E-08	5.40	2	2409.20195	-2.76	40.43	0
	IHFPLATYAPVISAEK	7	12		0.0000	0	0.00008848	4.88	3	1756.96177	-0.87	32.09	0
	AVFVDLEPTVIDEVR	6	12		0.0000	0	0.00001549	4.73	2	1701.90386	-1.18	36.07	0
	TIGGGDDSFNTFFSETGAGK	4	17		0.0000	0	0.000009676	4.56	2	2007.88628	-3.42	34.99	0
	AVcMLSNTTAIAEAWAR	2	12	C3(Carbamidomethyl)	0.0000	0	0.00002046	4.49	2	1864.89250	-6.44	36.18	0
	lDHKFDLMYAK	1	14	N-Term(Acetyl)	0.0000	0	0.001405	4.27	3	1422.70786	-0.56	28.28	1
	QLFHPEQLITGK	4	25		0.0000	0	0.00009124	3.99	2	1410.77165	-1.69	29.18	0
	LDHKFDLMYAK	4	14		0.0000	0	0.0007673	3.86	3	1380.69614	-1.41	24.87	1
	TIQFVDWcPTGFK	1	11	C8(Carbamidomethyl)	0.0000	0	0.0001157	3.44	2	1598.76641	-0.53	35.97	0
	NLDIERPTYTNLNR	2	16		0.0000	0	0.0008995	3.09	2	1718.88078	-0.75	26.50	0
	aVFVDLEPTVIDEVR	1	12	N-Term(Acetyl)	0.0000	0	0.000166	3.08	2	1743.91435	-1.19	41.10	0
	DVNAAIATIK	2	8		0.0000	0	0.008629	3.07	2	1015.57555	-2.70	26.59	0
	VGINYQPPTVVPGGDLAK	5	14		0.0000	0	0.002757	2.97	2	1824.98235	-1.73	31.26	0
	EIIDLVLDR	4	9		0.0000	0	0.009746	2.69	2	1085.62041	0.23	32.01	0
	YMAccLLYR	1	9	C4(Carbamidomethyl); C	0.0000	0	0.002007	2.69	2	1249.55034	-1.86	29.47	0
Nucleophosmin OS=Homo sapiens GN=NPM1	1761.709878	75.85	9	25	25	523	265	29.44624075	4.614746094				
	Sequence	# PSMs	# Proteins	Modifications	ΔCn	q-Value	PEP	XCorr	Charge	MH+ [Da]	ΔM [ppm]	RT [min]	# Missed Cleavages
	TVSLGAGAKDELHIVEAEAMNYEGSPIK	85	3		0.0000	0	2.032E-11	9.28	3	2929.44742	-4.74	32.43	1
	ADKDYHFKVDNDENEHQLSLR	22	3		0.0000	0	2.902E-12	8.52	3	2573.19870	-1.13	19.31	2
	TVSLGAGAKDELHIVEAEAmNYEGSPIK	17	3	M20(Oxidation)	0.0000	0	2.441E-08	8.50	3	2945.44956	-2.26	34.56	1
	LAADEDDDDDDEEDDDEDDDDDDFDDEEAEEKAPVKK	6	3		0.0000	0	0.000001006	6.89	4	4246.54116	-2.27	27.07	2
	DELHIVEAEAMNYEGSPIK	16	3		0.0000	0	1.829E-07	6.29	3	2145.01352	-1.60	35.65	0
	DYHFKVDNDENEHQLSLR	3	3		0.0000	0	7.083E-08	5.61	3	2259.04026	-1.02	25.64	1
	mSVQPTVSLGGFEITPPVVLR	34	4	M1(Oxidation)	0.0000	0	4.175E-07	5.55	2	2243.20439	-2.74	38.34	0
	mEDSMDMDMSPLRPQNYLFGcELK	9	3	N-Term(Acetyl); C21(Ca	0.0000	0	5.039E-07	5.38	3	2949.25302	-2.27	38.07	0
	tVSLGAGAKDELHIVEAEAMNYEGSPIK	1	3	N-Term(Acetyl)	0.0000	0	0.0001726	5.31	4	2971.46523	-2.24	37.08	1
	aDKDYHFKVDNDENEHQLSLR	4	3	N-Term(Acetyl)	0.0000	0	8.309E-08	5.30	3	2615.21939	2.76	24.60	2
	DELHIVEAEAmNYEGSPIK	18	3	M11(Oxidation)	0.0000	0	8.533E-07	5.27	3	2161.00650	-2.49	31.27	0
	MSVQPTVSLGGFEITPPVVLR	38	4		0.0000	0	0.000002384	5.14	2	2227.21269	-1.31	35.37	0
	mEDSmDMDMSPLRPQNYLFGcELK	23	3	N-Term(Acetyl); M5(Oxi	0.0000	0	0.000006647	4.98	3	2965.24308	-3.90	36.22	0
	vDNDENEHQLSLR	9	4	N-Term(Acetyl)	0.0000	0	0.000001175	4.83	2	1610.73796	-1.62	19.49	0
	mSVQPTVSLGGFEITPPVVLR	13	4	N-Term(Acetyl)	0.0000	0	0.000002544	4.70	2	2269.22344	-1.21	42.86	0
	VDNDENEHQLSLR	50	4		0.0000	0	0.00001015	4.65	3	1568.72696	-1.94	15.33	0
	MTDQEAIQDLWQWR	7	2		0.0000	0	0.00001201	4.61	2	1819.83611	-3.94	39.23	0
	dELHIVEAEAMNYEGSPIK	1	3	N-Term(Acetyl)	0.0000	0	0.00844	4.49	3	2187.02597	-0.71	32.37	0
	lAADEDDDDDDEEDDDEDDDDDDFDDEEAEEKAPVKK	1	3	N-Term(Acetyl)	0.0000	0	0.000008311	4.38	4	4288.55971	-0.39	24.40	2
	LAADEDDDDDDEEDDDEDDDDDDFDDEEAEEKAPVK	1	3		0.0000	0	7.07E-08	4.33	3	4118.44260	-3.22	24.86	1
	mTDQEAIQDLWQWR	1	2	N-Term(Acetyl)	0.0000	0	0.00003535	4.20	2	1861.84416	-5.20	41.50	0
	mEDSmDmDMSPLRPQNYLFGcELK	7	3	N-Term(Acetyl); M5(Oxi	0.0000	0	0.00003608	4.14	3	2981.24466	-1.64	35.00	0
	MQASIEKGGSLPK	6	2		0.0000	0	0.0001726	3.66	2	1345.70952	-3.69	13.68	1
	TPKGPSSVEDIK	9	3		0.0000	0	0.0009346	3.63	2	1257.66802	-0.44	12.31	1
	mTDQEAIQDLWQWR	2	2	M1(Oxidation)	0.0000	0	0.00006225	3.44	2	1835.83342	-2.60	34.19	0
	mQASIEKGGSLPK	7	2	M1(Oxidation)	0.0000	0	0.0004197	3.36	2	1361.70708	-1.71	13.94	1
	GPSSVEDIKAK	5	3		0.0000	0	0.001558	3.33	3	1130.60401	-1.09	13.74	1
	FINYVKNcFR	4	2	C8(Carbamidomethyl)	0.0000	0	0.0007655	3.30	3	1360.68079	-1.69	22.43	1
	LAADEDDDDDDEEDDDEDDDDDDFDDEEAEEK	1	3		0.0000	0	0.00008274	2.96	3	3723.20542	0.77	26.12	0
Serine/arginine-rich splicing factor 5 OS=Homo sapiens GN=SRSF5	170.3095706	27.94	6	7	8	52	272	31.24528543	11.59033203				
	Sequence	# PSMs	# Proteins	Modifications	ΔCn	q-Value	PEP	XCorr	Charge	MH+ [Da]	ΔM [ppm]	RT [min]	# Missed Cleavages
	QAGEVTFADAHRPK	22	2		0.0000	0	0.0007068	4.87	4	1526.76735	-2.45	12.58	0
	lNEGVVEFASYGDLK	3	2	N-Term(Acetyl)	0.0000	0	0.000005841	4.56	2	1682.83061	1.99	33.04	0
	GFGFVEFEDPRDADDAVYELDGK	4	5		0.0000	0	0.00002552	4.00	3	2591.15183	-2.15	34.57	1
	LNEGVVEFASYGDLK	4	2		0.0000	0	0.00004575	3.69	2	1640.80339	-8.11	30.21	0
	GFGFVEFEDPR	4	5		0.0000	0	0.0003414	2.89	2	1299.59758	-2.25	31.54	0
	DADDAVYELDGK	2	6		0.0000	0	0.0003943	2.88	2	1310.57170	-2.31	24.82	0
	LIVENLSSR	8	8		0.0000	0	0.008403	2.38	2	1030.58867	-0.50	20.20	0
	lIVENLSSR	2	8	N-Term(Acetyl)	0.0000	0	0.005387	2.11	2	1072.59770	-1.90	32.37	0
60S ribosomal protein L6 OS=Homo sapiens GN=RPL6	2357.256814	68.4	7	33	33	988	288	32.70763034	10.57958984				
	Sequence	# PSMs	# Proteins	Modifications	ΔCn	q-Value	PEP	XCorr	Charge	MH+ [Da]	ΔM [ppm]	RT [min]	# Missed Cleavages
	HQEGEIFDTEKEKYEITEQR	33	1		0.0000	0	8.055E-10	7.31	3	2509.17826	-2.38	22.09	2
	hQEGEIFDTEKEKYEITEQR	14	1	N-Term(Acetyl)	0.0000	0	1.454E-07	6.70	3	2551.18760	-2.82	23.63	2
	QLASGLLLVTGPLVLNR	109	1		0.0000	0	0.000003401	5.87	2	1764.06841	-3.32	35.54	0
	QLASGLLLVTGPLVLNRVPLRR	3	1		0.0000	0	0.0001659	5.71	4	2385.47890	-1.20	32.47	2
	ASITPGTILIILTGRHR	2	4		0.0000	0	0.00001373	5.48	4	1819.08986	-0.80	32.87	1
	qLASGLLLVTGPLVLNR	17	1	N-Term(Acetyl)	0.0000	0	0.000001949	5.20	2	1806.08025	-2.54	38.49	0

VLATVTKPVGGDKNGGTR	3	6		0.0000	0	0.000005866	5.10	3	1769.98368	-1.83	10.62	1
HQEGEIFDTEKEK	27	1		0.0000	0	0.0006259	4.89	3	1589.74161	-1.67	13.44	1
HQEGEIFDTEK	17	1		0.0000	0	0.00001201	4.88	2	1332.60222	-3.35	15.69	0
eKVLATVTKPVGGDK	7	6	N-Term(Acetyl)	0.0000	0	0.00006081	4.76	3	1583.90128	0.56	18.14	1
EKVLATVTKPVGGDK	8	6		0.0000	0	0.00003067	4.41	3	1541.88810	-1.12	13.77	1
sVFALTNGIYPHK	3	1	N-Term(Acetyl)	0.0000	0	0.00001775	4.33	2	1488.78520	0.41	28.42	0
QLASGLLLVTGPLVLNRVPLR	1	1		0.0000	0	0.0005082	4.28	3	2229.37772	-1.31	34.26	1
VLATVTKPVGGDK	89	6		0.0000	0	0.0005606	4.20	2	1284.75066	-1.24	12.95	0
hQEGEIFDTEKEK	12	1	N-Term(Acetyl)	0.0000	0	0.0004806	4.18	3	1631.75571	0.54	16.63	1
SVFALTNGIYPHKLVF	3	1		0.0000	0	0.0000901	4.11	2	1805.99285	-1.15	32.59	1
SVFALTNGIYPHK	8	1		0.0000	0	0.00009149	3.98	2	1446.77104	-2.06	25.87	0
hQEGEIFDTEK	11	1	N-Term(Acetyl)	0.0000	0	0.00001705	3.96	2	1374.61809	0.61	19.61	0
ASITPGTILIILTGR	79	4		0.0000	0	0.0005484	3.94	3	1525.92890	-1.56	38.67	0
HLTDAYFKK	14	1		0.0000	0	0.0008581	3.86	2	1122.59184	-2.14	12.25	1
KIDQKAVDSQILPK	1	1		0.0000	0	0.0003542	3.83	2	1582.91313	-2.04	16.48	2
IDISNVKIPK	2	1		0.0000	0	0.0009151	3.73	2	1126.68169	-1.25	22.21	1
aSITPGTILIILTGR	15	4	N-Term(Acetyl)	0.0000	0	0.00004455	3.72	2	1567.93987	-1.26	41.64	0
IKAIPQLQGYLR	4	1		0.0000	0	0.001207	3.43	3	1399.84101	-0.75	25.26	1
hLTDAYFK	20	1	N-Term(Acetyl)	0.0000	0	0.0009781	3.40	2	1036.50847	-1.33	23.14	0
vLATVTKPVGGDK	4	6	N-Term(Acetyl)	0.0000	0	0.0001064	3.34	2	1326.76177	-0.79	20.91	0
THQKFVIATSTK	2	1		0.0000	0	0.0001925	3.32	2	1360.75517	-2.35	10.11	1
HLTDAYFK	72	1		0.0000	0	0.00203	3.25	2	994.49858	-0.70	16.01	0
IPKHLTDAYFK	1	1		0.0000	0	0.0004346	2.83	2	1332.72905	-1.53	18.58	1
hLTDAYFKK	4	1	N-Term(Acetyl)	0.0000	0	0.002547	2.81	2	1164.60491	0.08	18.34	1
KGKPHcSRNPVLVR	2	6	C6(Carbamidomethyl)	0.0000	0	0.004736	2.76	4	1647.92287	0.25	9.13	2
aVDSQILPK	43	1	N-Term(Acetyl)	0.0000	0	0.0007101	2.70	2	1012.56737	-0.02	21.14	0
EKYEITEQR	8	1		0.0000	0	0.01104	2.67	2	1195.59221	-2.65	12.08	1
YYPTEDVPRK	9	4		0.0000	0	0.0009458	2.51	3	1267.62979	-1.55	14.35	1
eKYEITEQR	5	1	N-Term(Acetyl)	0.0000	0	0.009544	2.47	2	1237.60344	-2.02	15.92	1

Supplementary File 2

siRNA sequences

siRNAs	Sequences
USP7-1	GACGUUUCGAAUAGAGGAA
USP7-2	GCACUAAUGCUUACAUGUU
USP7-3	GACUUUGAGAACAGGCGAA
USP7 5'UTR-1	CUCACCUCGUCAGCCACUA
ECT2-1	GCACUCACCUUGUAGUUGA
ECT2-2	CAGAGGAGAUUAAGACUAU
ECT2 5'UTR-1	GGUGGAACUCCUAGGGCUU
ECT2 5'UTR-2	CCGGCGAGGAAUGGCGGUA
USP11-1	AATGAGAATCAGATCGAGTCC
USP11-2	AAGGCAGCCTATGTCCTCTTC
UHRF1-1	GCCAUACCCUCUUCGACUATT
UHRF1-2	AGGUGGUCAUGCUCAACUACA
RAD18-1	GAGCAUGGAUUAUCUAUUCAA
RAD18-2	UUAUAAAUGCCCAAGGAAAUU
MDM2-1	GCCAGTATATTATGACTAA
MDM2-2	AAUGGUUGCAUUGUCCAUGGC
RNF168-1	GACACUUUCUCCACAGAUA
RNF168-2	GGCGAAGAGCGAUGGAAGA

Lentiviral shRNA sequences

shRNAs	Sequences
Control	CCGGGATATGGGCTGAATACAAACTCGAGTTTGTATTCAGCCCATATCTTTTTG
ECT2-1	CCGGGCCCGTTGTATTGTACAAGTACTCGAGTACTTGTACAATACAACGGGCTTTTTG
ECT2-2	CCGGCGGAATGAACAGGATTTCTATCTCGAGATAGAAATCCTGTTCATTCCGTTTTTG
ECT2-3	CCGGCCAGCAATGATAAGCATGTAACTCGAGTTACATGCTTATCATTGCTGGTTTTTG

Note: Red color indicates the targeting sequence against the corresponding genes.

qRT-PCR primers

Genes	Sequences
GAPDH	F: GAAGGTGAAGGTCGGAGTC
	R: GAAGATGGTGATGGGATTTC
USP7	F: ATTCCTAACATTGCCACCAG
	R: ATTTACACCATTTGCCATCC
USP7 (5'UTR)	F: TCCAAGCTGGTGTGTTCAAG
	R: CAGCGAATCCTCTTGCTGAA
ECT2	F: TGTAGTCACGGACTTTCAGGA
	R: GTACAATACAACGGGCGACAT
MDM2	F: GAATCATCGGACTCAGGTACATC
	R: TCTGTCTCACTAATTGCTCTCCT
PHF8	F: AGGACAAGGAAAGCGTCCCAA
	R: ACACAGGAGGGCTCACAGAA
RNF168	F: TCAACGTGGAACTGTGGACG
	R: CAGGTTTACTGAGCAGACGAAC
TP53	F: CAGCACATGACGGAGGTTGT
	R: TCATCCAAATACTCCACACGC
p21	F: CTTGTGGAGCCGGAGCT
	R: TGGTGTCTCGGTGACAAAGT
PUMA	F: GACCTCAACGCACAGTACGAG
	R: AGGAGTCCCATGATGAGATTGT
TP53INP1	F: TTCCTCCAACCAAGAACCAGA
	R: GCTCAGTAGGTGACTCTTCACT
TP53INP2	F: GCTGGTTTGTTACCCCTCCC
	R: GGTGACGTAAACGGACATGCT
GDF15	F: ACCTGCACCTGCGTATCTCT
	R: CGGACGAAGATTCTGCCAG
IGFBP3	F: AGACACACTGAATCACCTGAAGT
	R: AGGGCGACACTGCTTTTTCTT

Supplementary File 3

RNA-seq Analysis of Co-regulated Genes by ECT2 and USP7

Gene	log2Ratio(Control_siRNA-VS-ECT2_siRNA)	log2Ratio(Control_siRNA-VS-USP7_siRNA)	Symbol
9635	2.998578	2.783536	CLCA2
3486	2.918072	2.814632	IGFBP3
9518	2.895453	2.552165	GDF15
100506696	2.711829	2.228975	KDM5B-AS1
1026	2.70063	2.877388	CDKN1A
55065	2.663808	3.810116	SLC52A1
8537	2.590283	1.404705	BCAS1
3635	2.457357	2.937028	INPP5D
3488	2.35554	1.108654	IGFBP5
51566	2.315407	1.867743	ARMCX3
7832	2.310668	3.678548	BTG2
6662	2.306474	2.208475	SOX9
3909	2.294314	1.588172	LAMA3
5655	2.189877	1.890735	KLK10
8835	2.107414	3.834778	SOCS2
1056	2.070578	2.537098	CEL
2872	2.027905	1.927106	MKNK2
2934	2.007105	2.449209	GSN
638	1.998578	2.372467	BIK
90427	1.997231	2.209523	BMF
2066	1.945951	1.785485	ERBB4
624	1.921771	1.901916	BDKRB2
8000	1.887314	2.250477	PSCA
5337	1.875562	1.826604	PLD1
11259	1.867948	1.896994	FILIP1L
80352	1.866406	1.902747	RNF39
94241	1.86241	1.859026	TP53INP1
4322	1.827306	1.191601	MMP13
27113	1.827306	2.086785	BBC3
2329	1.827306	2.367173	FMO4
55384	1.791327	1.223437	MEG3
401320	1.772024	1.432128	LOC401320
58476	1.751271	1.645622	TP53INP2
1649	1.747426	1.230851	DDIT3
80726	1.698023	4.259564	KIAA1683
8609	1.686907	1.423026	KLF7
23105	1.686214	2.998429	FSTL4
55062	1.67678	1.324368	WIPI1
9901	1.669765	1.223437	SRGAP3
7139	1.66765	2.865424	TNNT2
55911	1.657381	2.079891	APOBR
360	1.650495	1.70821	AQP3
8228	1.638861	2.12206	PNPLA4
1647	1.604914	1.330647	GADD45A
79603	1.604914	2.037711	CERS4
144811	1.585549	1.182748	LACC1
59	1.579379	3.188024	ACTA2
1850	1.559373	1.620239	DUSP8
132671	1.556004	2.107741	SPATA18
57198	1.550789	2.058226	ATP8B2
27076	1.543747	1.28475	LYPD3
7045	1.522021	1.203528	TGFBI
151354	1.516325	1.38986	FAM84A
100505687	1.505378	2.039598	LOC100505687
2258	1.488505	1.253791	FGF13
80256	1.483899	3.018967	FAM214B
220	1.481085	-1.314651	ALDH1A3
54103	1.460524	1.392708	GSAP
730091	1.454047	1.602118	LOC730091
121643	1.444449	1.933026	FOXN4
253190	1.442185	1.648129	SERHL2
153222	1.434989	1.952135	CREBRF
7042	1.426577	-1.666775	TGFB2
400073	1.425944	1.537098	C12orf76
1942	1.423774	1.014599	EFNA1

64063	1.421314	1.350059	PRSS22
387264	1.42105	1.174528	KRTAP5-1
10133	1.406975	1.632517	OPTN
134111	1.401318	1.436677	UBE2QL1
5264	1.39192	1.522884	PHYH
1843	1.39032	1.388999	DUSP1
51136	1.373588	1.259024	RNFT1
10396	1.360027	1.023394	ATP8A1
9935	1.350597	2.935647	MAFB
84952	1.34188	1.551173	CGNL1
79720	1.34188	1.050437	VPS37B
9149	1.335453	2.515565	DYRK1B
54850	1.32928	1.405853	FBXL12
10457	1.315407	2.077666	GPNMB
51646	1.314237	1.667884	YPEL5
10608	1.302526	1.876753	MXD4
112483	1.296283	1.727069	SAT2
2627	1.29297	1.274063	GATA6
2034	1.284585	1.588193	EPAS1
150962	1.282986	1.21517	PUS10
148523	1.263065	1.741112	C1orf51
54800	1.259527	1.024739	KLHL24
9536	1.258111	1.041773	PTGES
129303	1.254417	1.429457	TMEM150A
7464	1.23568	1.319867	CORO2A
94121	1.234964	1.571045	SYTL4
6253	1.234964	2.344453	RTN2
10043	1.234395	1.236589	TOM1
84532	1.223485	2.004268	ACSS1
23542	1.217891	3.222224	MAPK8IP2
10628	1.212026	1.556884	TXNIP
6303	1.205964	1.455414	SAT1
51092	1.200524	1.754903	SIDT2
23135	1.195477	1.119863	KDM6B
8418	1.193719	1.047047	CMAHP
57037	1.189877	1.352066	ANKMY2
1408	1.180416	1.137691	CRY2
6560	1.177493	1.631074	SLC12A4
9764	1.176921	1.12206	KIAA0513
60401	1.159676	2.046772	EDA2R
1513	1.150348	1.415107	CTSK
8974	1.142808	1.070077	P4HA2
5652	1.131885	1.067821	PRSS8
4758	1.127592	1.101596	NEU1
25946	1.122665	1.620308	ZNF385A
57491	1.119487	1.612386	AHRR
284749	1.100441	1.278786	LINC00494
7008	1.075447	2.228077	TEF
1318	1.073063	1.51285	SLC31A2
901	1.058426	1.239868	CCNG2
355	1.056477	1.876186	FAS
3155	1.056293	1.195406	HMGCL
53349	1.050602	1.717149	ZFYVE1
55902	1.035511	1.162058	ACSS2
283219	1.031356	1.104138	KCTD21
81790	1.03084	1.037024	RNF170
1831	1.026662	1.442119	TSC22D3
154091	1.019952	2.138325	SLC2A12
83667	1.016496	1.666841	SESN2
80271	1.012496	1.197713	ITPKC
5376	1.009225	1.035191	PMP22
6004	1.001573	1.493845	RGS16
55260	1.001336	1.561551	TMEM143
4784	-1.00111	-2.249499	NFIX
1719	-1.003353	-1.151688	DHFR
55159	-1.007972	-1.061759	RFWD3
84930	-1.010422	-1.856723	MASTL
4796	-1.010866	-1.324767	TONSL
55573	-1.013733	-1.184171	CDV3
3397	-1.015799	-1.202111	ID1
55055	-1.019577	-1.244494	ZWILCH

79075	-1.020206	-1.632827	DSCC1
85439	-1.02069	-1.335147	STON2
81930	-1.023117	-1.434888	KIF18A
109	-1.023117	-1.238087	ADCY3
90411	-1.023483	-1.691035	MCFD2
7023	-1.025852	-1.103006	TFAP4
29127	-1.028121	-1.04149	RACGAP1
5984	-1.028547	-1.121238	RFC4
9908	-1.031032	-1.06196	G3BP2
24137	-1.035791	-1.330378	KIF4A
79723	-1.037499	-1.026364	SUV39H2
128239	-1.037534	-1.1775	IQGAP3
4751	-1.038263	-1.157918	NEK2
5985	-1.039605	-1.520618	RFC5
81624	-1.041449	-1.443003	DIAPH3
6790	-1.042924	-1.644172	AURKA
9055	-1.047728	-1.245605	PRC1
7019	-1.060968	-1.22796	TFAM
4605	-1.063177	-1.131768	MYBL2
9401	-1.063366	-1.349183	RECQL4
54821	-1.064113	-1.731391	ERCC6L
9129	-1.068478	-1.295792	PRPF3
81610	-1.068533	-1.665899	FAM83D
64105	-1.068706	-2.964341	CENPK
3227	-1.069053	-1.13687	HOXC11
2305	-1.069536	-1.341262	FOXM1
1058	-1.070814	-1.405417	CENPA
5347	-1.071298	-1.31556	PLK1
79915	-1.072698	-1.897243	ATAD5
79066	-1.074078	-1.303745	METTL16
90417	-1.077785	-1.132181	KNSTRN
55143	-1.078677	-1.223362	CDCA8
55013	-1.081135	-1.375227	CCDC109B
6891	-1.083999	-1.290978	TAP2
84515	-1.084637	-1.715883	MCM8
5983	-1.089538	-1.284904	RFC3
7112	-1.090444	-1.672704	TMPO
55165	-1.091839	-1.886401	CEP55
25939	-1.092523	-1.047865	SAMHD1
1033	-1.097315	-1.815546	CDKN3
10615	-1.103521	-1.333739	SPAG5
201725	-1.103623	-1.386033	C4orf46
5422	-1.10412	-1.144191	POLA1
9787	-1.106581	-1.321067	DLGAP5
78995	-1.106806	-1.174622	C17orf53
54556	-1.10716	-1.065352	ING3
167227	-1.107866	-1.379882	DCP2
147015	-1.111293	-1.134021	DHRS13
993	-1.122737	-1.600837	CDC25A
699	-1.123379	-1.54813	BUB1
3619	-1.12689	-1.102885	INCENP
2237	-1.128258	-1.800873	FEN1
332	-1.129386	-2.197203	BIRC5
79071	-1.141687	-1.878679	ELOVL6
3978	-1.144632	-1.081065	LIG1
26227	-1.144902	-1.497172	PHGDH
254102	-1.148836	-1.31181	EHBP1L1
90381	-1.150957	-1.923645	TICRR
4288	-1.15236	-1.893526	MKI67
991	-1.161245	-1.550989	CDC20
11113	-1.174525	-1.040198	CIT
63967	-1.178137	-1.578098	CLSPN
10024	-1.180393	-1.483837	TROAP
1111	-1.185597	-2.05178	CHEK1
54069	-1.191553	-1.185368	MIS18A
337873	-1.195438	-1.560934	HIST2H2BC
1848	-1.195777	-1.173396	DUSP6
8877	-1.198229	-1.116989	SPHK1
81704	-1.199362	1.683541	DOCK8
51537	-1.202441	-1.167164	MTFP1
10376	-1.212314	-1.213081	TUBA1B

55215	-1.213108	-1.746811	FANCI
11004	-1.213539	-1.735921	KIF2C
5557	-1.216707	-1.790164	PRIM1
145508	-1.217088	-1.391819	CEP128
259266	-1.222258	-2.125263	ASPM
91452	-1.224671	-1.302979	ACBD5
10535	-1.225525	-1.256582	RNASEH2A
9521	-1.227976	-1.447795	EEF1E1
4176	-1.229667	-1.65454	MCM7
22995	-1.230592	-1.111995	CEP152
6671	-1.239435	-1.397449	SP4
8318	-1.239916	-2.333267	CDC45
3835	-1.242544	-1.201367	KIF22
890	-1.253255	-3.359692	CCNA2
9212	-1.254456	-1.388235	AURKB
55635	-1.261036	-1.751384	DEPDC1
11169	-1.262331	-1.110021	WDHD1
7298	-1.264175	-1.428421	TYMS
11247	-1.271511	-1.276134	NXPH4
675	-1.272958	-1.600066	BRCA2
9194	-1.275504	-1.077859	SLC16A7
150468	-1.275939	-2.286269	CKAP2L
4173	-1.278143	-1.546779	MCM4
2139	-1.279609	-1.24051	EYA2
7272	-1.282825	-1.698565	TTK
9700	-1.285629	-1.415729	ESPL1
3833	-1.285969	-1.657177	KIFC1
3070	-1.292433	-1.564263	HELLS
701	-1.292537	-1.861547	BUB1B
6566	-1.292931	-2.60586	SLC16A1
10293	-1.293709	-1.446414	TRAIP
26271	-1.295736	-1.416404	FBXO5
3832	-1.295791	-1.314312	KIF11
2187	-1.301977	-1.822305	FANCB
55247	-1.301977	-1.78483	NEIL3
55723	-1.302794	-1.403682	ASF1B
4172	-1.304453	-1.593777	MCM3
10036	-1.304948	-1.668896	CHAF1A
8208	-1.310694	-1.030587	CHAF1B
23649	-1.311701	-1.680687	POLA2
3837	-1.317059	-1.067386	KPNB1
11065	-1.317586	-1.076816	UBE2C
113130	-1.319235	-2.052775	CDCA5
10721	-1.320592	-1.574822	POLQ
51512	-1.320808	-1.420567	GTSE1
116832	-1.322441	-1.469691	RPL39L
4174	-1.3231	-1.586604	MCM5
254263	-1.324003	-1.092259	CNIH2
29028	-1.3304	-1.765389	ATAD2
54619	-1.332034	-2.003921	CCNJ
1869	-1.332259	-1.46719	E2F1
57082	-1.33538	-1.84006	CASC5
284403	-1.33627	-1.506812	WDR62
51203	-1.336368	-1.621906	NUSAP1
161742	-1.336742	-1.440182	SPRED1
5424	-1.344963	-1.020796	POLD1
51514	-1.351559	-2.389422	DTL
1063	-1.351945	-1.488087	CENPF
5888	-1.352466	-1.727464	RAD51
11130	-1.355498	-1.59182	ZWINT
221150	-1.365642	-1.968297	SKA3
9088	-1.367072	-1.798293	PKMYT1
195828	-1.37116	-3.142583	ZNF367
7153	-1.377918	-1.267234	TOP2A
2491	-1.378598	-1.905846	CENPI
9833	-1.386674	-1.686983	MELK
10403	-1.393124	-1.680722	NDC80
672	-1.393607	-2.252609	BRCA1
1958	-1.408892	-2.592185	EGR1
983	-1.414731	-1.671208	CDK1
7083	-1.423051	-1.959958	TK1

146909	-1.424987	-1.610801	KIF18B
23279	-1.425573	-1.906012	NUP160
83540	-1.4355	-1.570431	NUF2
83990	-1.442962	-1.905194	BRIP1
83879	-1.44433	-2.38439	CDCA7
9770	-1.444717	-1.111995	RASSF2
2177	-1.450857	-1.748304	FANCD2
80150	-1.454651	-1.128553	ASRGL1
10733	-1.456951	-1.601118	PLK4
7516	-1.468709	-2.306599	XRCC2
56992	-1.474813	-1.505635	KIF15
54892	-1.474873	-1.648747	NCAPG2
3014	-1.48962	-1.001456	H2AFX
10592	-1.48996	-1.544541	SMC2
51361	-1.491122	-1.527506	HOOK1
5427	-1.492697	-1.397014	POLE2
55872	-1.49382	-2.246134	PBK
1870	-1.507678	-1.449963	E2F2
9493	-1.512781	-1.411385	KIF23
10460	-1.514448	-1.550671	TACC3
4171	-1.515289	-1.549462	MCM2
9156	-1.515716	-2.550365	EXO1
79019	-1.517705	-1.895862	CENPM
29893	-1.522576	-1.654522	PSMC3IP
55355	-1.536011	-2.156847	HJURP
374393	-1.54378	-3.190823	FAM111B
4085	-1.545329	-1.522236	MAD2L1
64946	-1.554285	-1.136674	CENPH
4603	-1.556906	-1.508313	MYBL1
5933	-1.559536	-1.532195	RBL1
9824	-1.56007	-1.657788	ARHGAP11A
6941	-1.567923	-1.946779	TCF19
990	-1.578686	-2.483003	CDC6
220042	-1.587911	-2.840152	C11orf82
9133	-1.5935	-1.264894	CCNB2
23397	-1.600318	-1.970697	NCAPH
51659	-1.608681	-1.844505	GINS2
29128	-1.611404	-2.094258	UHRF1
10635	-1.616541	-1.632827	RAD51AP1
79682	-1.620675	-2.375552	MLF1IP
22974	-1.621634	-1.43693	TPX2
157313	-1.623017	-1.484382	CDCA2
54478	-1.639558	-1.674953	FAM64A
100128191	-1.646311	-2.330799	TMPO-AS1
145773	-1.652474	-3.398362	FAM81A
157570	-1.665615	-2.055359	ESCO2
1138	-1.673387	-1.187268	CHRNA5
4940	-1.675194	-1.135328	OAS3
163786	-1.682505	-1.381933	SASS6
55388	-1.712261	-2.341504	MCM10
2118	-1.714758	-2.460646	ETV4
55789	-1.722157	-2.251554	DEPDC1B
115207	-1.723273	-2.992723	KCTD12
8438	-1.730476	-2.193431	RAD54L
79733	-1.732611	-2.323989	E2F8
83461	-1.763655	-2.078111	CDCA3
100131211	-1.787403	-1.340646	TMEM194B
147841	-1.787403	-1.78483	SPC24
4998	-1.806798	-1.985038	ORC1
9319	-1.810921	-1.590392	TRIP13
347240	-1.819112	-1.471891	KIF24
9837	-1.819584	-1.520618	GINS1
55270	-1.882122	-3.227472	NUDT15
145270	-1.886939	-2.177148	PRIMA1
203547	-1.891873	-1.876593	VMA21
162681	-1.908965	-2.429294	C18orf54
641	-1.924339	-2.059269	BLM
80178	-1.931139	-2.098491	C16orf59
83903	-1.967558	-2.369793	GSG2
9134	-1.973096	-2.419424	CCNE2
64151	-1.980048	-1.972314	NCAPG

79801	-1.994216	-1.651748	SHCBP1
2119	-2.024836	-1.799606	ETV5
55329	-2.099788	-1.65859	MNS1
79968	-2.114978	-2.15478	WDR76
26150	-2.149973	-2.310899	RIBC2
2583	-2.196366	-1.786861	B4GALNT1
4900	-2.198472	-2.6448	NRGN
6023	-2.243083	-2.825472	RMRP
3674	-2.395086	-2.462902	ITGA2B
7161	-2.43948	-2.507296	TP73
6241	-2.464507	-2.559674	RRM2
126567	-2.680488	-1.748304	C2CD4C
8626	-2.702515	-5.092259	TP63
9768	-3.354684	-1.325203	KIAA0101
3008	-3.838029	-2.905846	HIST1H1E
348738	-8.206569	-8.206569	C2orf48
